# Isopropylammonium as a Dual-Role Cation: Spacer and Perovskitizer in Ruddlesden–Popper Lead Bromides

**DOI:** 10.1021/acs.inorgchem.6c01079

**Published:** 2026-04-29

**Authors:** Mirosław Mączka, Anna Gągor, Barbara Popanda, Maciej Ptak, Dagmara Stefańska, Katarzyna Fedoruk-Piskorska, Jan K. Zaręba, Adam Sieradzki

**Affiliations:** † 215275 Institute of Low Temperature and Structure Research, Polish Academy of Sciences, Okólna 2, Wrocław 50-422, Poland; ‡ Department of Experimental Physics, 49567 Wrocław University of Science and Technology, Wybrzeże Wyspiańskiego 27, Wrocław 50-370, Poland; § August Chelkowski Institute of Physics, University of Silesia in Katowice, Chorzow 41-500, Poland; ∥ Institute of Advanced Materials, Faculty of Chemistry, Wrocław University of Science and Technology, Wybrzeże Wyspiańskiego 27, Wrocław 50-370, Poland

## Abstract

Layered hybrid perovskites are attractive optoelectronic materials for a wide range of applications. This study reports three layered Ruddlesden–Popper (RP) bromides comprising small isopropylammonium (IPA^+^) cations: IPA_2_MA_2_Pb_3_Br_10_, IPA_2_DMAPb_2_Br_7_, and IPA_2_(IPA_0.77_MHy_0.23_)­Pb_2_Br_7_ (MA^+^ = methylammonium, DMA^+^ = dimethylammonium, MHy^+^ = methylhydrazinium). Crucially, the IPA_2_(IPA_0.77_MHy_0.23_)­Pb_2_Br_7_ compound represents a unique case where IPA^+^ cations occupy the perovskite cavities, thus acting as a perovskitizer, a dual role previously unobserved for this cation in lead halide perovskites. All compounds undergo structural phase transitions, the mechanisms of which are analyzed based on X-ray diffraction, Raman spectroscopy, and dielectric data. Nonlinear optical (NLO) and dielectric studies demonstrate that IPA_2_MA_2_Pb_3_Br_10_ exhibits second-harmonic generation (SHG) at low temperatures and bistable dielectric switching. Photoluminescence (PL) studies reveal that IPA_2_MA_2_Pb_3_Br_10_ displays narrowband purplish-blue emission attributed to free excitons (FEs), whereas the other compounds exhibit broadband PL attributed to self-trapped excitons (STEs). The broadband emission shows remarkable thermochromism, transitioning from yellow (orange) at 80 K to blue (white) at high temperatures for IPA_2_DMAPb_2_Br_7_ and IPA_2_(IPA_0.77_MHy_0.23_)­Pb_2_Br_7_, respectively. These results exemplify the unusual ability of IPA^+^ to function simultaneously as a spacer and a perovskitizer, highlighting the critical role of A-site cations in modulating the structural, electric, and optical properties of multilayered perovskites.

## Graphical abstract

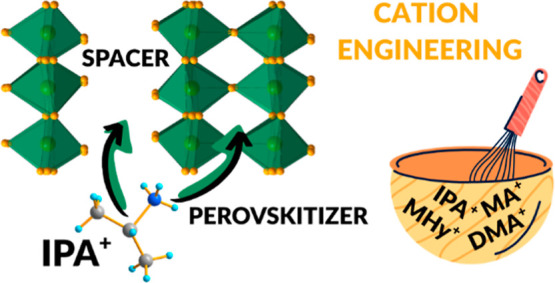

## Introduction

In the 21st century, interest in developing materials for optoelectronic devices has surged. Beyond rapid and low-cost production, these materials must satisfy several criteria, including tunable band gaps and PL, high absorption coefficients, high PL efficiency, and processing flexibility.^1–3^ Hybrid lead halide perovskites^2,3^ meet these requirements and crystallize in various dimensionalities. Three-dimensional (3D) lead halide perovskites with the general formula APbX_3_ (where A and X denote an organic cation and halogen ion Cl, Br, I, respectively) are renowned for their photovoltaic and light-emitting properties.^2–6^ Two-dimensional (2D) structures constitute another vital subgroup,^7–14^ primarily categorized into Ruddlesden–Popper (RP) and Dion-Jacobson (DJ) phases.^7–14^ Among (100)-oriented structures, RP phases are characterized by (1/2, 1/2) offsets between adjacent inorganic layers, whereas DJ phases exhibit direct overlap or (1/2,0)/(0,1/2) offsets.

RP phases typically form with large monovalent interlayer cations, following the general formula A’_2_A_*n*–1_Pb_*n*_X_3*n*+1_ (A’ = large monovalent interlayer cation, A = small cage cation, *n* = number of octahedral layers within each inorganic slab).^4,7–10,13,14^ Conversely, DJ phases generally incorporate diammonium cations with the formula A^”^A_*n*–1_B_*n*_X_3*n*+1_ (A” = large divalent interlayer cation).^7,8,12^ 2D perovskites offer several advantages, such as higher PL quantum yield (PLQY), increased exciton binding energy, and superior stability compared to 3D perovskites.^7,8^ These properties facilitate applications in LEDs,^9,10^ solar cells,^12,13^ lasers,^14,15^ photodetectors,^16,17^ nonlinear optics^18–20^ and ferroelectric devices.^19–21^ However, for solar cell applications narrow band gaps are necessary and this feature can be achieved for iodides only. Dimensionality can also be reduced to one-dimensional (1D) or zero-dimensional (0D)^22–25^ which are attractive as light-emitting^11,22,23^ and switchable-dielectric materials.^24,25^

The selection of an appropriate spacer cation is critical for synthesizing 2D perovskites with targeted properties. The most important factors for selecting a cation are its size, shape, charge, and functional groups influence, since these factors affect the crystal structure and, consequently, the optical (e.g., band gap, type, color and efficiency of PL),^7,8,11,19,20,26–31^ and electrical properties (e.g., ferroelectricity).^19–21,32^ For aliphatic amines, chain length significantly impacts optical properties of perovskite.^33,34^ In case of *n* ≥ 2 perovskites, the choice of the A-site cation, known as the “perovskitizer”, is equally important, as it modulates optoelectronic properties.^12,35^ Furthermore, proper choice of the A-site cations may induce polar order, as reported for many *n* ≥ 2 compounds comprising methylmmonium (MA^+^), formamidinium (FA^+^), ethylammonium (EA^+^) or methylhydrazinium (MHy^+^) cations.^16,21,36,37^ Apart of these cations, *n* ≥ 2 lead halide perovskites were also reported for dimethylammonium (DMA^+^), 1,2,4-triazolium (Tz^+^) and guanidinium (GA^+^) organic cations.^38,39^

Only a few 2D lead halide perovskites comprising IPA^+^ were reported so far. Important examples are 2D bromides with the formula IPA_2_Tz_*n*–1_Pb_*n*_Br_3*n*+1_ (*n* = 1–3) reported by Li et al.^38^ These compounds exhibit broad emission, with the energy band gap decreasing from 2.95 eV (*n* = 1) to 2.70 eV (*n* = 3) due to large intraoctahedral distortion caused by the accommodation of the large Tz^+^ cation in the perovskite cages.^38^ Recently, the IPA_2_PbBr_4_ (*n* = 1) bromide was reported to exhibit ferroelectric properties.^40^ Another example of 2D bromide is IPA_2_CsPb_2_Br_7_.^41^ This compound is an interesting example of an above-room-temperature (RT) antiferroelectric perovskite (*T*_*c*_ = 353 K). Among iodides, a 2D structure has been reported for IPA_2_MAPb_2_I_7_ only.^42^ Due to the small size of the interlayer IPA^+^ cation, this compound possesses the smallest interlayer distance (5.15 Å) among known *n* ≥ 2 lead iodide perovskites. However, it crystallizes in a centrosymmetric tetragonal structure, precluding polar order and second-order (NLO properties, and exhibits weak PL.^42^ It is worth noting that the incorporation of IPA^+^ into the cages of 2D lead halide perovskites has not been previously reported. While IPA^+^ has been shown to fit into the cages of 2D tin halide perovskites—such as in IPA_3_SnI_7_ (*n* = 2 RP phase), where IPA^+^ acts as both a spacer and a perovskitizer cation,^43^ this structure is only stable at high temperatures, converting to thermodynamically stable IPA_3_SnI_5_ upon cooling.^43^

Here, we report the synthesis of three novel perovskites with short interlayer distances comprising IPA^+^ cations: the *n* = 3 phase IPA_2_MA_2_Pb_3_Br_10_, and the *n* = 2 phases IPA_2_DMAPb_2_Br_7_ and IPA_2_(IPA_0.77_MHy_0.23_)­Pb_2_Br_7_. We demonstrate that the type of perovskitizer cation regulates the PL character, ranging from narrow emission for MA^+^ to broadband emission for DMA^+^ and the mixed IPA/MHy system. We also report that all compounds undergo structural phase transitions (PTs) associated with organic cation ordering. Most interestingly, IPA_2_MA_2_Pb_3_Br_10_ shows efficient SHG activity below 260 K and switchable dielectric properties. Furthermore, IPA_2_(IPA_0.77_MHy_0.23_)­Pb_2_Br_7_ constitutes the first example of a lead bromide perovskite where IPA^+^ is located within the perovskite cages. Our study highlights the impact of the A-site cation on the structural, optical, and electrical properties of RP phases comprising small IPA^+^ interlayer cations.

## Experimental Section

### Synthesis

All reagents were commercially purchased from Sigma-Aldrich and used without further purification (PbBr_2_ 99%, HBr 48% in H_2_O, isopropylamine 99.5%, methylhydrazine 98%, methylamine (2 M solution in methanol) and dimethylamine (2 M solution in methanol)).

All compounds were synthesized by cooling of the reaction mixtures from 60 °C to RT with a rate of 1 °C/h. The reaction mixtures contained 6 mmol of PbBr_2_, 8 mmol of isopropylamine and 3 mmol of methylamine, dimethylamine or methylhydrazine. Yellow, light-yellow and pale-yelow plate-like crystals of IPA_2_MA_2_Pb_3_Br_10_, IPA_2_DMAPb_2_Br_7_ and IPA-MHy lead bromide, respectively, which grew at the bottom of the glass vials, were separated from the mother liquids and dried at RT. Since Raman spectrum of IPA-MHy crystals showed rather small intensity of MHy^+^ related bands, we decided to establish their composition using NMR method. The NMR spectrum shows that the methyl groups of MHy^+^ and IPA^+^ cations give rise to the presence of a singlet at 2.56 ppm and a doublet at 1.13 ppm, respectively (Figure S1). The integration of these peaks and calculation of their ratios showed that the chemical composition corresponds to IPA_2_(IPA_0.77_MHy_0.23_)­Pb_2_Br_7_ formula. We have also tried to grow single crystals of IPA_2_MHyPb_2_Br_7_ by regulating IPA/MHy/Pb ratio and IPA_2_IPAPb_2_Br_7_ by using IPA/Pb ratio 1.5:1 and 1:1 but these attempts failed.

Photos of the obtained crystals are presented in Figure S2. The experimental powder diffraction patterns of the ground IPA_2_DMAPb_2_Br_7_ and crystals IPA_2_(IPA_0.77_MHy_0.23_)­Pb_2_Br_7_ show good agreement with the patterns simulated based on the single-crystal data, confirming their purity (Figure S3). For IPA_2_MA_2_Pb_3_Br_10_ determination of the single crystal structure failed and, therefore, this compound is not included in Figure 3.

### NMR Spectroscopy

Quantitative determination of the organic amine composition of the sample containing MHy^+^ and IPA^+^ cations was performed with the use of ^1^H NMR spectroscopy. ^1^H NMR spectra were recorded in a liquid state on the Jeol JNM-ECZ 400S Research FT NMR spectrometer (JEOL Ltd., Tokyo, Japan) operating at 400 MHz. The sample was prepared by dissolving ∼5 mg of single crystals in ca. 0.7 mL of DMSO-*d*_6_, followed by warming to 50 °C and thorough sonication. Next, clear solutions were transferred to 5 mm Wilmad NMR tubes.

### Powder X-ray Diffraction

Powder X-ray diffraction (PXRD) patterns of the ground crystals were measured in the reflection modes using an X’Pert PRO X-ray diffraction system equipped with a PIXcel ultrafast line detector and Soller slits for CuKα_1_ radiation (λ = 1.54056 Å). Low-temperature (LT) diffraction patterns were collected using a closed-cycle helium cryostat (Oxford Cryosystems PheniX). Finely ground powder samples were mixed with Apiezon grease, and the measurements were carried out under vacuum.

### Differential Scanning Calorimetry (DSC)

DSC curves were detected using a Mettler Toledo DSC-3 calorimeter in the nitrogen atmosphere. The heating/cooling rate was 5 K min^–1^ and the measured sample masses were 10.55, 15.10, and 7.07 mg for IPA_2_MA_2_Pb_3_Br_10_, IPA_2_DMAPb_2_Br_7_, and IPA_2_(IPA_0.77_MHy_0.23_)­Pb_2_Br_7_, respectively. Measurements were performed in the temperature range of 120–400 K. The excess heat capacity associated with the PTs was calculated by subtracting from the data the baseline representing the system variation in the absence of the PT.

### Single-Crystal X-ray Diffraction

Single-crystal X-ray diffraction measurements were performed on a four-circle Xcalibur diffractometer using Mo Kα radiation and an Atlas CCD detector. Data were collected with ω-scan mode employing a step size of Δω = 1°. Absorption corrections were applied using multiscan procedures implemented in CrysAlis PRO 1.171.42.93a (Rigaku Oxford Diffraction, 2023), including spherical harmonics through the SCALE3 ABSPACK scaling algorithm. Structure solution and refinement were carried out within the Olex2 environment using SHELXT and SHELXL.^44–47^ Hydrogen atoms were introduced at geometrically calculated positions only for ordered nitrogen and carbon atoms and refined using the riding model. A summary of crystallographic parameters and refinement statistics for RT phase of IPA_2_DMAPb_2_Br_7_ and RT and LT polymorphs of IPA_2_(IPA_0.77_MHy_0.23_)­Pb_2_Br_7_ is provided in Table S1. Due to the formation of polydomain structure in IPA_2_DMA Pb_2_Br_7_ below 240 K, resulting in splitting and smearing of the diffracted peak intensities, only the RT phase could be solved. The diffraction patterns (0kl and 1kl reciprocal layers) of the LT phase are provided in Figure S4. It was not possible to solve the structure of IPA_2_MA_2_Pb_3_Br_10_ as crystal selection was complicated by twinning and by the platelet morphology leading to inseparable stacked crystals.

### Raman Spectroscopy

RT FT-Raman (3500–50 cm^–1^ range) and FT-IR spectra (3500–400 cm^–1^ range) were measured for powdered crystals using a Bruker FT MultiRam spectrometer with YAG/Nd laser excitation (λ_exc_ = 1064 nm) and a Nicolet iS50 spectrometer, respectively. Temperature-dependent Raman spectra were measured using a Renishaw inVia Raman spectrometer equipped with a confocal DM2500 Leica optical microscope and a CCD detector. For IPA_2_(IPA_0.77_MHy_0.23_)­Pb_2_Br_7_, the excitation was done using an argon laser (λ_exc_ = 514 nm) and the spectral range was 3400–150 cm^–1^. Due to the luminescence background, Raman spectra of IPA_2_MA_2_Pb_3_Br_10_ and IPA_2_DMAPb_2_Br_7_ were measured using a diode laser (λ_exc_ = 830 nm), which allowed observation of Raman modes in the 1670–200 cm^–1^ range. Additional measurements in the low-wavenumber range (250–10 cm^–1^) were performed with the same Renishaw spectrometer using an Eclipse filter. Temperature of the samples was controlled using a THMS600 temperature control stage (Linkam). The spectral resolution of all Raman and IR spectra was 2 cm^–1^.

### Broadband Dielectric Spectroscopy (BDS)

Broadband dielectric spectroscopy was carried out using a Broadband Impedance Novocontrol Alpha-A analyzer. Measurements were performed on single crystal samples with dimensions of 1.3 mm^2^ × 0.1 mm and 3.0 mm^2^ × 0.2 mm for IPA_2_MA_2_Pb_3_Br_10_ and IPA_2_DMAPb_2_Br_7_, respectively. Silver paste was applied to the parallel surfaces of the single crystals to ensure good electrical contact. A sinusoidal voltage with an amplitude of 1 V and a frequency in the range of 1 Hz–1 MHz was applied across the samples. The temperature was stabilized using nitrogen gas using the Novocontrol Quattro system.

### Nonlinear Optical (NLO) Studies

Nonlinear optical (NLO) experiments were performed using a laser system employing a wavelength-tunable Topaz Prime Vis-NIR optical parametric amplifier (OPA) pumped by Coherent Astrella Ti/sapphire regenerative amplifier providing femtosecond laser pulses (800 nm, 75 fs) at 1 kHz repetition rate. The output of OPA was set to 1500 nm and the laser fluence at samples was equal 0.20 mJ/cm^2^. The single crystals of IPA_2_MA_2_Pb_3_Br_10_, IPA_2_DMAPb_2_Br_7_, and IPA_2_(IPA_0.77_MHy_0.23_)­Pb_2_Br_7_, and KDP were crushed with a spatula and sieved through an Aldrich mini-sieve set, collecting a microcrystal size fraction of 88–125 μm. Next, size-graded samples were fixed in-between microscope glass slides to form tightly packed layers, sealed, and mounted to the horizontally aligned sample holder. No refractive index matching oil was used. The employed measurement setup operates in the reflection mode. Specifically, the laser beam was directed onto the sample at 45 deg to its surface. Emission collecting optics consisted of a Ø25.0 mm plano-convex lens of focal length 25.4 mm mounted to the 400 μm 0.22 NA glass optical fiber and was placed along the normal to the sample surface. The distance between collection lens and the sample was equal to 30 mm. The spectra of the temperature-dependent NLO responses were recorded by an Ocean Optics Flame T XR fiber-coupled CCD spectrograph with 200 μm entrance slit. Scattered pumping radiation was suppressed with the use of a Thorlabs 750 nm hard-coated short-pass dielectric filter. The temperature control of the sample was performed using a Linkam LTS420 Heating/Freezing Stage. Temperature stability was equal to 0.1 K. TR-SHG study of IPA_2_MA_2_Pb_3_Br_10_ was conducted in a range of 143 to 303 K. Kurtz-Perry powder test was performed by comparing the SHG signal of IPA_2_MA_2_Pb_3_Br_10_ at 143 K with that of the KDP standard at 293 K. Signal integrations times were 2000 and 1000 ms for IPA_2_MA_2_Pb_3_Br_10_ and KDP, respectively. NLO screenings for IPA_2_DMAPb_2_Br_7_, and IPA_2_(IPA_0.77_MHy_0.23_)­Pb_2_Br_7_ were performed in 143 K–303 and 88 K–393 K ranges, respectively.

### Optical Absorption and PL Studies

The RT diffuse reflectance spectra of the powdered samples were obtained with a Cary 5E UV–Vis–NIR spectrophotometer (Varian, Palo Alto, CA, USA). Temperature-dependent PL measurements were carried out using 360 nm excitation from diode-laser source, and the emitted light was analyzed with a Hamamatsu PMA-12 multichannel detector equipped with a BT-CCD linear sensor (Hamamatsu Photonics, Iwata, Japan). The temperature of the samples during measurements was regulated with high accuracy using a Linkam THMS 600 Heating/Freezing Stage (Linkam Scientific Instruments, Tadworth, UK).

## Results and Discussion

### DSC

DSC traces of IPA_2_MA_2_Pb_3_Br_10_ reveal two thermal anomalies observed at *T*_1_ = 256 K (261 K) and *T*_2_ = 234 K (235 K) during cooling (heating), respectively (Figure S5). Significant thermal hysteresis, the symmetric shape of the anomaly, and the sharp change in entropy at *T*_1_ (Figures 1a and S5) indicate that this PT is of first-order character. In contrast, the absence of thermal hysteresis, the broad and asymmetric shape of the anomaly, and the continuous change in entropy over a temperature range of approximately 25 K suggest that the PT at *T*_2_ is of second-order character (Figure 1a). The associated changes in enthalpy Δ*H* and entropy Δ*S* were estimated to be 4.94 kJ mol^–1^ and ∼19.46 J mol^–1^ K^–1^ for the PT at *T*_1_, and ∼0.99 kJ mol^–1^ and ∼4.29 J mol^–1^ K^–1^ for the PT at *T*_2_. For an order–disorder PT, Δ*S* = *R*ln­(*N*), where *R* is the gas constant and *N* is the ratio of the number of configurations in the disordered and ordered phases. The estimated N values are 10.4 and 1.67 for the PT at *T*_1_ and *T*_2_, respectively. These results suggest that both PTs have order–disorder character, with the transition at *T*_1_ involving significantly greater structural rearrangements.

**Figure 1:**
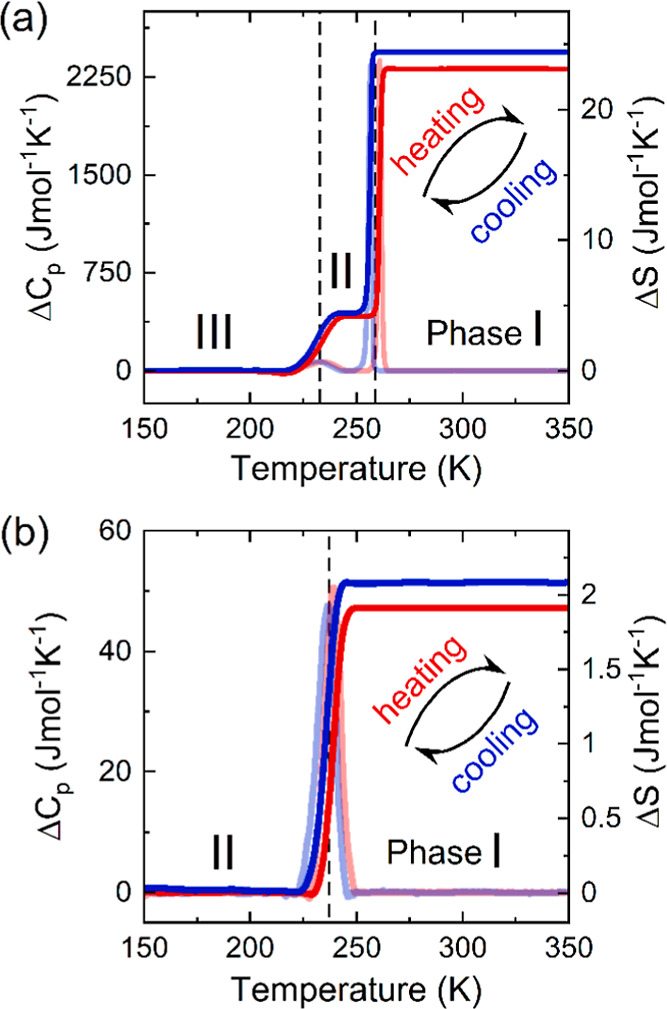
Temperature dependence of the excess heat capacity and entropy obtained in the heating (red) and cooling (blue) run for (a) IPA_2_MA_2_Pb_3_Br_10_ and (b) IPA_2_DMAPb_2_Br_7_.

In the case of IPA_2_DMAPb_2_Br_7_, only one anomaly is observed at 237 K (240 K) during cooling (heating), respectively (Figure S6). The symmetrical shape of this anomaly and step-like change of the entropy (Figures S6 and 1b) indicates a first-order character of this PT. The associated changes in enthalpy Δ*H* and entropy Δ*S* are ∼0.48 kJ mol^–1^ and ∼1.99 J mol^–1^ K^–1^, respectively. The small value of the estimated *N* (1.27) is consistent with a small change in disorder at the PT.

In contrast to IPA_2_MA_2_Pb_3_Br_10_ and IPA_2_DMAPb_2_Br_7_, DSC measurements of IPA_2_(IPA_0.77_MHy_0.23_)­Pb_2_Br_7_ show a very weak and broad anomaly near 250 K (Figure S7) that may result from a strongly suppressed PT.

### NLO Studies

To probe the symmetry of the crystal phases and characterize the PT behavior of IPA_2_MA_2_Pb_3_Br_10_, IPA_2_DMAPb_2_Br_7_, and IPA_2_(IPA_0.77_MHy_0.23_)­Pb_2_Br_7_, temperature-dependent screening of nonlinear optical (NLO) properties was performed. Obtained NLO emissions of these materials will be discussed in turn.

A powdered sample of IPA_2_MA_2_Pb_3_Br_10_ was irradiated with 1500 nm femtosecond laser pulses over a temperature range of 143 to 303 K. A characteristic feature of the resulting TR-SHG plot is its unique profile, reflecting small temperature separation between two closely lying PTs of different mechanisms (Figure 2). Experimental spectra are shown in Figures S8 and S9. Specifically, the compound exhibits a clear SHG signal below 253 K, unequivocally confirming the noncentrosymmetric nature of LT and intermediate-temperature (IT) phases **II** and **III**. Upon heating from 143 K, the SHG intensity progressively decreases before undergoing a steeper drop to zero at approximately 250 K. The disappearance of the SHG response indicates a PT to a centrosymmetric, HT phase **I**. The subsequent reappearance of the SHG signal upon cooling occurs with ca. 10 K-wide temperature hysteresis, demonstrating first-order character of **I** → **II** PT, in agreement with the DSC data. Note that upon further cooling the SHG intensity also features gradual enhancement and exactly matches heating run. Accordingly, an underlying continuous enhancement of SHG intensity below **II** → **III** transformation is typical of a second-order PT. Thus, the SHG data confirm second-order character of this PT. This combined behavior distinguishes IPA_2_MA_2_Pb_3_Br_10_ from other layered hybrid perovskites, which display a sharp, first-order PT SHG profile between noncentrosymmetric and centrosymmetric crystal phases.^48,49^

**Figure 2:**
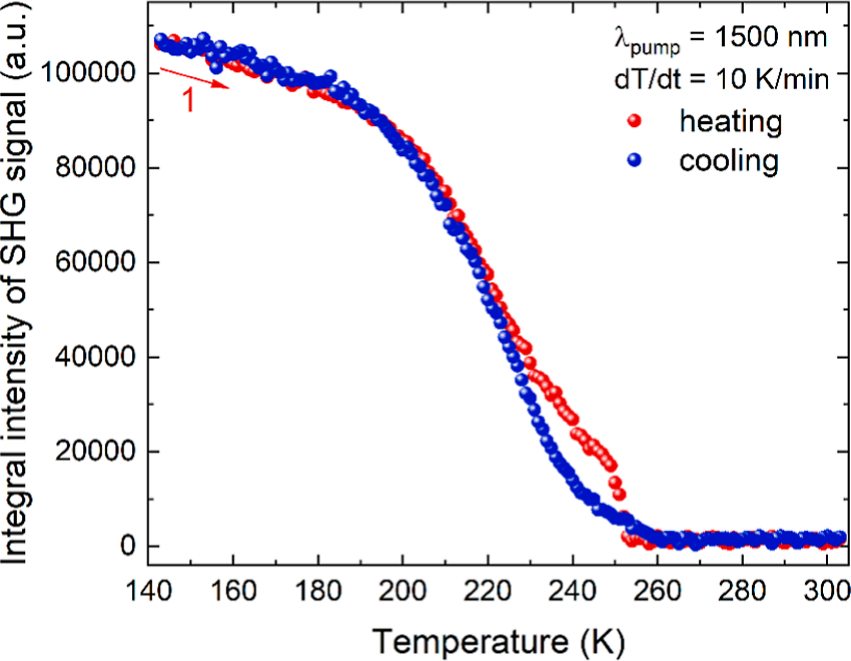
Integral SHG intensities plotted as a function of the temperature for IPA_2_MA_2_Pb_3_Br_10_.

To provide a quantitative measure of the SHG intensity, the Kurtz-Perry (Graja) powder test^50,51^ was performed by comparing the signal intensity of the IPA_2_MA_2_Pb_3_Br_10_ LT phase **III** (143 K) with that of potassium dihydrogen phosphate (KDP) at 293 K (Figure S10). The relative SHG efficiency of IPA_2_MA_2_Pb_3_Br_10_ was determined to be 0.036 times that of a KDP reference. This result indicates a modest, yet clearly measurable, second-order NLO activity in the noncentrosymmetric phase **III** of the compound.

Under identical excitation conditions temperature-dependent screenings of NLO properties were performed for IPA_2_DMAPb_2_Br_7_ (143 K–303 K) and IPA_2_(IPA_0.77_MHy_0.23_)­Pb2Br7 (88 K–393 K). In contrast to IPA_2_MA_2_Pb_3_Br_10_, these compounds have shown no measurable SHG activity in the studied temperature regimes. Only third-harmonic generation (THG) and relatively weak, broadband emissions of multiphoton absorption origin were detected, see Figures S11 and S12. Accordingly, all crystal phases of these compounds are deemed centrosymmetric.

### Single-Crystal X-ray Diffraction

Single-crystal and complementary powder X-ray diffraction studies were performed to investigate the crystal structures and structural changes occurring in these compounds during the PTs.

In the case of IPA_2_MA_2_Pb_3_Br_10_, the isolation of a monodomain single crystal was hindered by pronounced twinning and the material’s tendency to grow as thin, platelet-like crystallites stacked into inseparable aggregates. Consequently, we relied on temperature-dependent powder X-ray diffraction as the primary tool for tracking structural changes. Although a precise structural model for IPA_2_MA_2_Pb_3_Br_10_ remains unavailable, all observed diffraction peaks can be indexed within an orthorhombic unit cell with parameters *a* = 7.263(2) Å, *b* = 17.283(6) Å, and *c* = 23.280(7) Å. This cell can be derived as an orthorhombic 2b superstructure of the monoclinic IPA_2_Tz_2_Pb_3_Br_10_ phase, in which the [001] direction is normal to the perovskite layers.^38^

The relatively large unit-cell volume (2923 Å^3^) combined with only subtle temperature-induced changes in the diffraction patterns (Figures 3a,b and S13) indicates that the RT structure is already structurally relaxed, with no evidence of major symmetry breaking upon cooling. In particular, gradual shifts of the (004) Bragg reflection, which probes the interlayer (basal-plane) spacing, are observed (Figure 3b), suggesting the gradual freezing of the IPA^+^ dynamics upon cooling. A weak peak splitting emerging below 190 K may indicate a slight monoclinic distortion, plausibly associated with the **II** → **III** PT.

**Figure 3:**
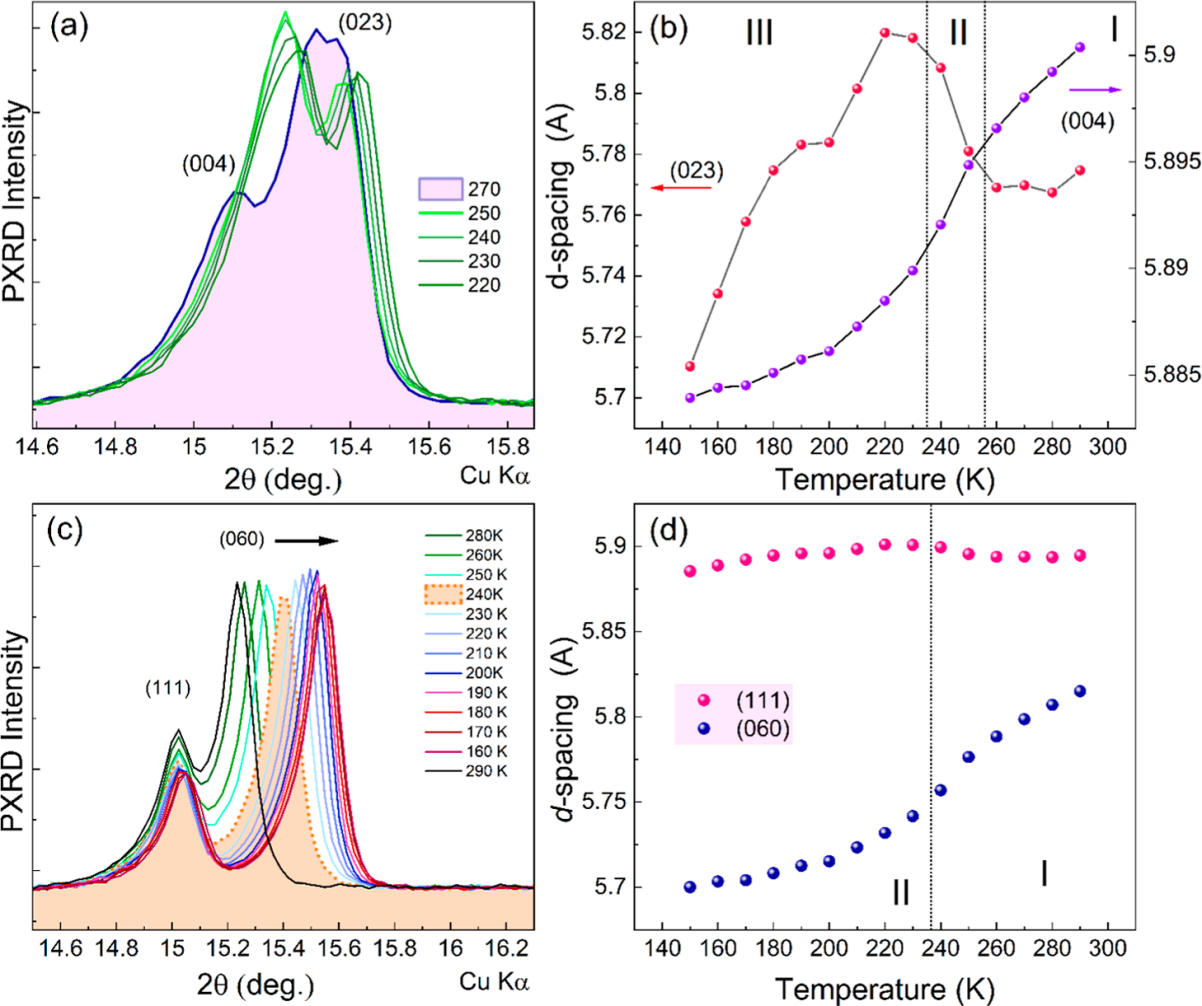
(a) Temperature evolution of the (004) and (320) diffraction peaks and (b) the corresponding changes in *d*-spacing in IPA_2_MA_2_Pb_3_Br_10_, indicating structural PTs. The *c*-axis is perpendicular to perovskite layers. (c) Temperature evolution of the (111) and (060) diffraction peaks and (d) the corresponding changes in *d*-spacing in IPA_2_DMAPb_2_Br_7_, indicating that PT predominantly affects the spacing between basal planes. The *b*-axis is perpendicular to the perovskite layers.

For IPA_2_DMAPb_2_Br_7_, the RT crystal structure was solved in the orthorhombic *Cmce* space group. Figure 4a,b presents both the overall packing and the detailed arrangement of the basic building units. At RT, both organic cations exhibit pronounced occupational disorder, which propagates into positional disorder of the apical and bridging bromine atoms within the inorganic framework. Due to N–H···Br hydrogen-bond (HB) interactions at the IPA–inorganic interface, the apical Br atoms are split over two crystallographic positions. Concurrently, the perovskite cages accommodate the large DMA^+^ cations through static displacements of Br atoms from their mean positions, as evidenced by enlarged atomic displacement parameters.

**Figure 4:**
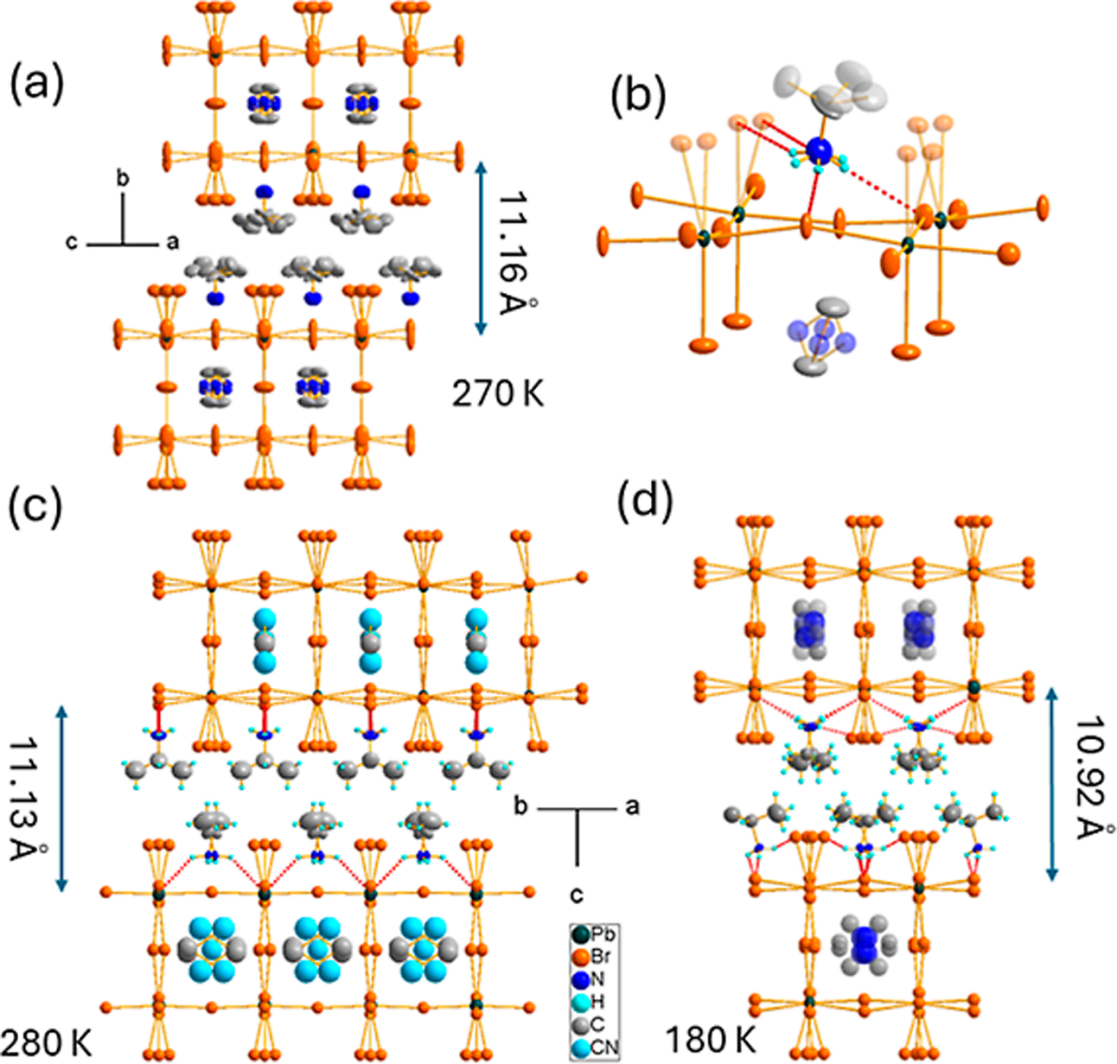
(a) Crystal structure of IPA_2_DMAPb_2_Br_7_ at 270 K; space group *Cmce*, *a* = 8.4750(5) Å, *b* = 34.933(3) Å, *c* = 8.5160(5) Å. (b) Disorder of the DMA^+^ and IPA^+^ moieties results in pronounced distortions of the PbBr_6_ octahedra. Sof of disordered Br:0.5, IPA:0.5, nitrogen from DMA:0.25. (c) Crystal structure of IPA_2_(IPA_0.77_MHy_0.23_)­Pb_2_Br_7_ at 280 K, tetragonal phase of *P*4_2_/*ncm* symmetry, *a* = 8.4763(2), *c* = 34.893(2) Å (b) LT phase of IPA_2_(IPA_0.77_MHy_0.23_)­Pb_2_Br_7_ at 180 K, orthorhombic *Pccn* space group with *a* = 8.4302(3), *b* = 8.4342(3), *c* = 34.418(2) Å.

The large interlayer separation of 11.16 Å is attributed to the rotational freedom of the IPA^+^ cations around the N–C bond, which expands the spacing between adjacent perovskite slabs. The temperature evolution of the (060) diffraction reflection (Figure 3c,d), together with the full set of powder diffractograms shown in Figure S14a, indicates a progressive slowing down of IPA^+^ dynamics. This is evidenced by pronounced shifts in the (060) reflection—which probes the distance between basal planes—in contrast to the (111) reflections, which exhibit only minor, nearly linear shifts consistent with ordinary thermal expansion. However, no discontinuity in the (060) reflection shift is observed at the PT temperature that could be attributed to a sudden arrest of IPA^+^ dynamics. Thermal evolution of the lattice parameters, determined from single-crystal data, is consistent with the PXRD results (see Figure S14). The largest relative changes are observed along the *b* direction, perpendicular to the perovskite layers. The continuous character of the observed anomalies indicates that the structural rearrangements in the phase transition region are subtle; however, they are accompanied by a lowering of the crystal symmetry, as evidenced by the splitting of diffraction peaks.

The mixed IPA_2_(IPA_0.77_MHy_0.23_)­Pb_2_Br_7_ crystals exhibit the highest degree of inorganic-framework disorder. This arises from the compositional mixing of large MHy^+^ and IPA^+^ cations within the perovskite cavities, highlighting the ability of IPA^+^ to act as a perovskitizer (A-site cation) in addition to its standard role as a spacer. At RT, the crystal structure is tetragonal (*P*4_2_/*ncm*), with the perovskite slabs oriented perpendicular to the direction. The IPA^+^ spacer cations are ordered; however, the large displacement ellipsoids associated with their methyl groups indicate pronounced librational motion about the N–C bond. The PT leads to a decrease in the interlayer separation from 11.30 Å at 280 K to 10.92 Å at 180 K, concomitant with a weak orthorhombic distortion leading to the *Pccn* structure (lattice parameters *a* = 8.4302(3) Å, *b* = 8.4342(3) Å, and *c* = 34.418(2) Å), where the N–C bonds of IPA^+^ spacers are tilted away from [001] direction. In the orthorhombic phase **II**, additional HBs form between the IPA^+^ spacer cations and the apical bromine atoms, resulting in increased separation between the split, disordered apical Br positions. While the mixed MHy/IPA perovskitizer cations remain disordered, the increase in both the number of split Br sites and their mutual separations is consistent with the strengthening and modification of HB interactions. The crystal structures of IPA_2_(IPA_0.77_MHy_0.23_)­Pb_2_Br_7_ at RT and in the LT phase are shown in Figure 4c,d.

### Temperature-Dependent Raman Study

To obtain deeper insight into the PT mechanisms and dynamics of the organic cations, we performed temperature-dependent Raman studies of the three compounds. To address the weak intensity of temperature-dependent Raman spectra in the internal modes region and challenges in measuring the spectra of IPA_2_MA_2_Pb_3_Br_10_ and IPA_2_DMAPb_2_Br_7_ in the stretching modes region (above 2900 cm^–1^), as well as to facilitate assignment of modes, we also measured RT FT-Raman and FT-IR spectra. The RT FT-Raman and FT-IR spectra of powdered samples are presented in Figures S15 and S16, respectively, while the temperature-dependent Raman spectra of single crystals are shown in Figures S17–S20. Plots of wavenumbers and full width at half-maximum (fwhm) values vs temperature for selected Raman modes of IPA_2_MA_2_Pb_3_Br_10_, IPA_2_DMAPb_2_Br_7_ and IPA_2_(IPA_0.77_MHy_0.23_)­Pb_2_Br_7_ are presented in Figure 5. The Wavenumbers of the vibrational modes are listed in Tables S2–S4 together with assignments based on Raman and IR data reported in the literature for hybrid perovskites comprising IPA^+^, DMA^+^, MA^+^ and MHy^+^ cations as well as DFT calculations for IPA^+^ and DMA^+^ cations.^48,52–56^

**Figure 5:**
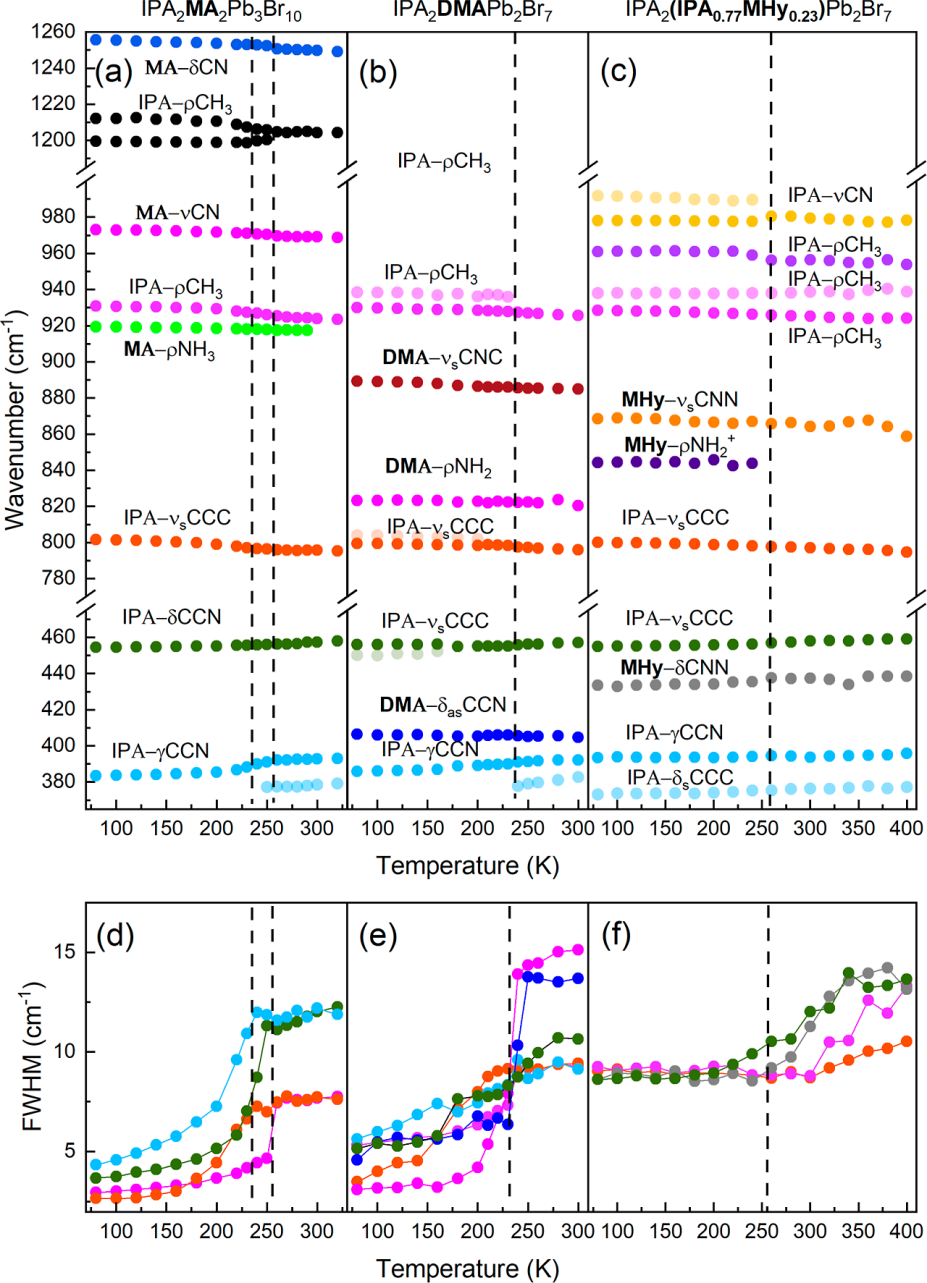
Temperature dependence of Raman wavenumbers for (a) IPA_2_MA_2_Pb_3_Br_10_, (b) IPA_2_DMAPb_2_Br_7_ and (c) IPA_2_(IPA_0.77_MHy_0.23_)­Pb_2_Br_7_, respectively. The corresponding fwhm are presented in panels (d–f), respectively. The color codes in panels (d–f) is the same as in panels (a–c). Vertical lines denote the PT temperatures.

We begin by discussing the temperature-dependent changes for IPA_2_MA_2_Pb_3_Br_10_. At 320 K, only two very strong bands at 22 and 31 cm^–1^, along with two shoulders at 55 and 137 cm^–1^ are observed in the lattice modes region (Figure S17a, Table S2). The substantial width of these bands indicates disorder in phase **I**. Upon cooling, sudden changes are observed between 260 and 250 K. First, the bands at 31 and 55 cm^–1^ shift to higher wavenumbers (36 and 61 cm^–1^, respectively, Figure S17a and Table S2). Second, the number of Raman bands doubles. Third, the bands exhibit significant narrowing. All these changes are consistent with a sudden decrease in disorder as well as an increase in the tilts and distortion of the PbBr_6_ octahedra, confirming the presence of a first-order PT associated with a symmetry reduction. No clear changes in the Raman spectra are observed near *T*_2_ but the Raman bands exhibit gradual narrowing and shifts upon further cooling to 80 K (Figure S17a). At 80 K, the bands are very narrow, and the number of discernible components increases to 15 (Table S2). These changes are consistent with the second-order character of the **II** → **III** PT and a well-ordered structure at very low temperatures.

Raman spectra in the internal modes region show that the PT at *T*_1_ leads to a narrowing of bands related to the MA^+^ cation. As a result, the δ­(CN) and ρ­(NH_3_) bands—barely visible at 1248 and 916 cm^–1^ (values at 260 K), respectively, become more distinct as the temperature decreases to 250 K (Figure S18). Due to the weak intensity of these bands, we plot the fwhm vs temperature for the much stronger ν­(CN) band observed at 968 cm^–1^. This plot shows a sudden drop in fwhm at *T*_1_ from 7.7 cm^–1^ at 260 K to 4.6 cm^–1^ at 250 K (Figure 5d). The observed narrowing of the MA-related bands indicates that the PT at *T*_1_ is associated with the ordering of MA^+^ cations. However, the wavenumbers of these bands are only weakly affected by the PT (see δ­(CN), ν_s_(CN) and ρ­(NH_3_) modes in Figure 5a), indicating minimal change in MA–framework interactions. Upon further cooling, the MA-related bands exhibit no anomalies at *T*_2_ (Figure 5a), but they gradually narrow, reaching very small fwhm values at 80 K (∼3 cm^–1^, see Figures S18 and 5d). This behavior indicates that these small cations possess a large degree of vibrational freedom just below *T*_2_ but these motions slow down upon cooling.

Distinct behavior is observed for the IPA-related bands. These bands exhibit a change in slope in the wavenumber vs temperature plots at *T*_1_ and *T*_2_ (Figure 5a), but they do not show any change in fwhm at *T*_1_ (in phase **II**, Figure 5d). Instead, they show a gradual decrease in fwhm below *T*_2_, as clearly observed for the γ­(CCN), δ­(CCN) and ν_s_(CCC) modes at 390, 456, and 795 cm^–1^, respectively (Figure 5d). The Raman data therefore indicate that the PT at *T*_2_ is most likely triggered by the gradual ordering of IPA^+^ cations. The Raman spectra also show that the MA-related bands, as well as the γ­(CCN), δ­(CCN) and ν_s_(CCC) modes of IPA^+^ do not exhibit any splitting down to 80 K (Figures S18 and 5a, Table S2). This suggests that phase **III** still comprises only one crystallographically unique MA^+^ and IPA^+^ cation.

We now turn to the Raman spectra of IPA_2_DMAPb_2_Br_7_. Figure S16b reveals the presence of very broad lattice bands at RT, consistent with significant disorder. These bands narrow upon cooling, although changes near *T*_1_ are hardly visible. Nevertheless, the PT leads to the splitting of the most intense band near 49 cm^–1^ into two components (54 + 47 cm^–1^ at 80 K), which is clearly observed at lower temperatures (Figure S17b and Table S3). However, the overall changes in the wavenumbers of the lattice bands are much less pronounced than those observed for IPA_2_MA_2_Pb_3_Br_10_, indicating that the PT in this compound induces much weaker changes in the tilts and distortion of the PbBr_6_ octahedra compared to the IPA_2_MA_2_Pb_3_Br_10_ analogue.

In the internal modes region, the PT does not lead to any distinct shifts in the Raman bands (Figures S19 and 5b). However, pronounced changes can be noticed for the fwhm of certain DMA-related bands (Figure 5e). For instance, the fwhm of the ρ­(NH_2_) and δ_as_(CCN) modes observed at 820 and 405 cm^–1^, respectively, decreases from 14.4 and 13.8 cm^–1^ at 250 K to 7.9 and 7.9 and 6.3 cm^–1^ at 230 K (Figure 5e). Such sudden drop in fwhm is consistent with a first-order character of the PT. Conversely, IPA-related modes do not exhibit a sudden drop in fwhm at *T*_1_, but instead show a gradual decrease upon cooling (Figure 5e). The Raman spectra suggest, therefore, that the PT in IPA_2_DMAPb_2_Br_7_ is associated with significant ordering of the DMA^+^ cations. Note that while no splitting is observed for the DMA-related modes, distinct splitting is evident for many IPA^+^ modes in phase **II** (Figures S19 and 5b, Table S3). Consequently, the Raman spectra indicate that the PT does not result in an increase in the number of crystallographically unique DMA^+^ cations; however, the number of unique IPA^+^ cations increases from one in phase **I** to two in phase **II**.

The RT Raman spectrum of IPA_2_(IPA_0.77_MHy_0.23_)­Pb_2_Br_7_ in the lattice modes region is quite similar to that observed for IPA_2_DMAPb_2_Br_7_, indicating similar octahedral tilts and distortion of the double inorganic layers (Figure S17c). Similar to IPA_2_DMAPb_2_Br_7_ splitting of the most intense band is also observed for IPA_2_(IPA_0.77_MHy_0.23_)­Pb_2_Br_7_ (appearing as two components at 53 and 46 cm^–1^ at 80 K; see Table S4 and Figure S20). However, this splitting is less distinct due to the broadening of these Raman bands; indeed, all bands corresponding to the Pb–Br stretch (151–99 cm^–1^), remain very broad even at 80 K (Figure 5c). Figure 5f shows that the internal bands of the IPA^+^ cations are also much broader at 80 K (fwhm near 8 cm^–1^) compared to the corresponding bands in IPA_2_DMAPb_2_Br_7_ (fwhm near 3–5 cm^–1^, Figure 5e). Therefore, the Raman spectra indicate the presence of static disorder in this compound, consistent with the mixing of IPA^+^ and MHy^+^ in the perovskite cages and in line with the substantial disorder revealed by X-ray diffraction. The temperature evolution of the Raman wavenumbers reveals that most modes do not exhibit changes attributable to the tetragonal-to-orthorhombic PT. However, the IPA-related ν­(CN) mode near 980 cm^–1^ and ρ­(CH_3_) mode near 956 cm^–1^ show clear shifts to lower and higher wavenumbers, respectively, near 250–260 K (Figure 5c). It is also noteworthy that the ρ­(NH_2_^+^) of MHy^+^ appears below 250 K, and the fwhm of all bands—which decreases noticeably down to 250 K—shows no further changes below this temperature (Figure 5f). All these observations suggest the occurrence of a PT near 250–260 K, associated with the arrest of the dynamic disorder of the organic cations.

### Dielectric Properties

To further elucidate the mechanisms associated with the PTs in IPA_2_MA_2_Pb_3_Br_10_ and IPA_2_DMAPb_2_Br_7_, and to investigate the dielectric properties of these perovskites, broadband dielectric spectroscopy measurements were performed. The temperature dependence of the dielectric permittivity (ε′) for IPA_2_MA_2_Pb_3_Br_10_ is presented in Figure 6a. The dielectric response reveals clear signatures of sequential PTs. At approximately 256 K, ε′ displays a pronounced, step-like discontinuity, confirming a first-order PT from phase **I** to phase **II**. Previous studies of hybrid perovskites have shownn that dielectric anomalies in these compounds arise primarily from changes in organic cation dynamics, which strongly affect the local polarizability and often lead to step-like variations in ε′.^25,57–59^ The observed step-like decrease of ε′ at 256 K therefore indicates that this PT is associated with a partial reduction in organic cation dynamics, in agreement with Raman data revealing ordering of MA^+^ cations. Notably, due to the presence of this sharp and reversible jump between low- and high-permittivity states, IPA_2_MA_2_Pb_3_Br_10_ exhibits robust dielectric switching (Figure 6b). Multiple cooling–heating cycles confirm the stability and reproducibility of this effect, demonstrating that the dielectric switching is intrinsic and arises from the structural PT between phases **I** and **II**. Switchable dielectrics are attractive materials for many applications, including switching, sensing, and memory devices, and have been reported for several halide and cyanide perovskites.^25,57–59^ Upon further cooling, a much subtler and continuous variation in ε′ emerges near 235 K (inset of Figure 6a), consistent with a second-order PT and further ordering of the organic cations. At elevated temperatures and low frequencies, a pronounced rise in ε′ is attributed to ionic conductivity.

**Figure 6:**
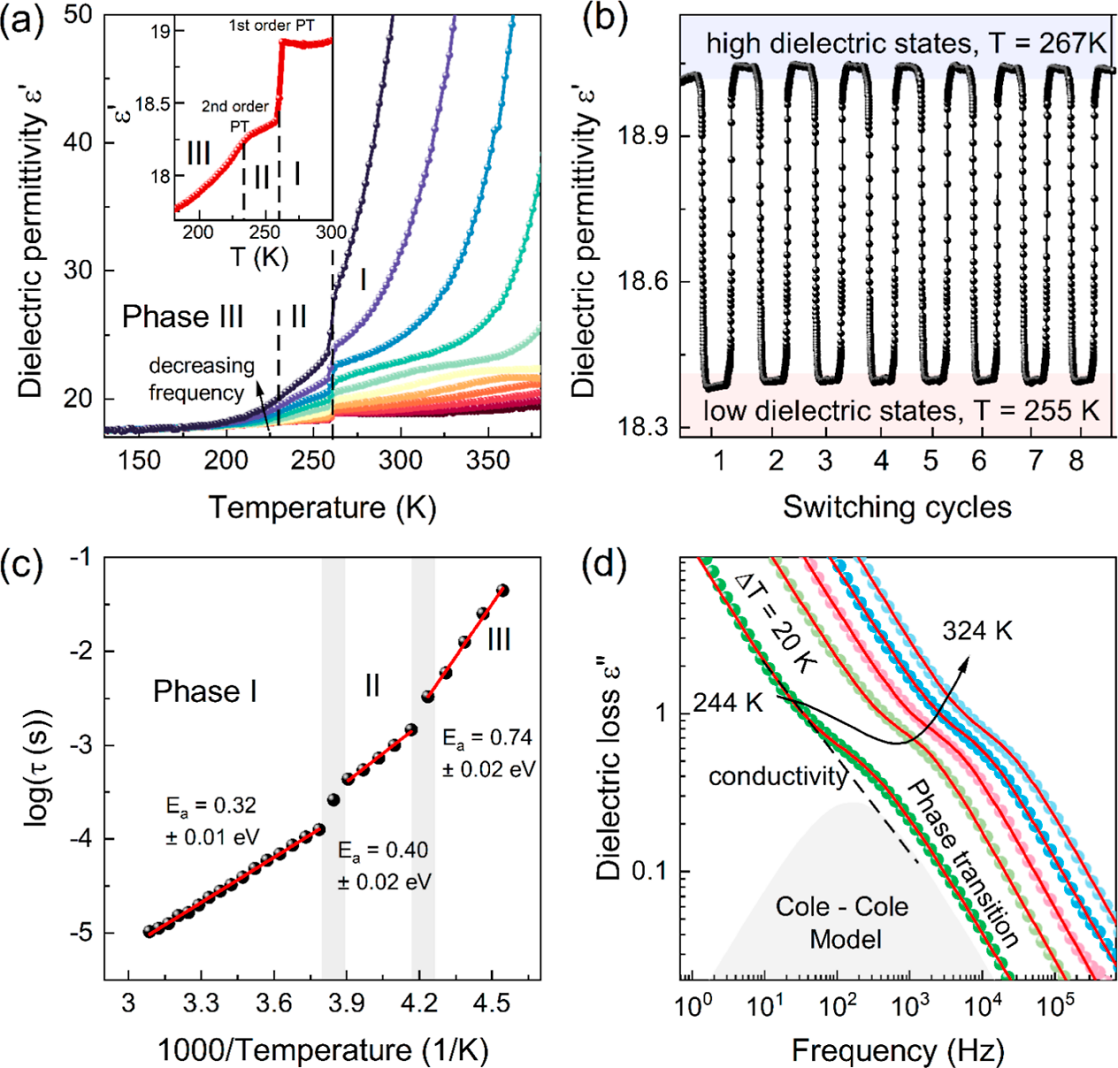
(a) Temperature dependence of the dielectric permittivity ε′ for IPA_2_MA_2_Pb_3_Br_10_ single crystals measured upon cooling. Representative curves are shown for selected frequency decades ranging from 1 Hz to 1 MHz. Dashed lines indicate the PT temperatures. (b) Several cycles of temperature-induced dielectric switching of IPA_2_MA_2_Pb_3_Br_10_ measured at a frequency of 5 kHz. (c) Inverse-temperature dependence of the relaxation time for IPA_2_MA_2_Pb_3_Br_10_. (d) Examples of fitted dielectric loss ε″ curves using the Cole–Cole function.

The dielectric loss (ε″) spectra as a function of frequency reveal a well-defined relaxation process in all three structural phases (Figure S21a). The ε″ curves were fitted using the Cole–Cole relaxation function augmented with a conductive term to account for the low-frequency tail arising from ionic transport (Figure 6d)

$$\varepsilon^{*}=\varepsilon_{\infty }+\frac{\Delta \varepsilon }{1+{\left(i\omega \tau \right)}^{1-\alpha }}+\frac{\sigma }{i\varepsilon_{0}\omega }$$

ε_∞_ is the permittivity at the high frequency limit, Δε = ε_s_–ε_∞_, where ε_*s*_ is the static, low frequency permittivity, τ the characteristic relaxation time, α the relaxation broadening parameter (0 ≤ α ≤ 1), and σ the dc conductivity.

It was noticed that in the studied temperature range, the relaxation times (τ) exhibit linear dependence on inverse temperature (1000/T) (Figure 6c). Therefore, the relaxation times can be modeled using the Arrhenius relation

$$\tau =\tau_{0}+\exp\frac{E_{a}}{k_{B}T}$$

where τ_0_, *k*_B_, and *E*_a_ are relaxation times at the high temperature limit, Boltzmann constant, and activation energy, respectively.

The activation energies obtained from the linear fits amount to 0.32 eV in phase **I**, 0.40 eV in phase **II**, and 0.74 eV in phase **III** (Figure 6c). The systematic increase in *E*_a_ upon cooling reflects the progressively higher energetic barrier for dipolar reorientation. Complementary Raman measurements reveal pronounced orientational disorder of the organic cations at RT and their ordering upon cooling. This structural ordering constrains cation motion, directly accounting for the observed rise in the activation energy of the relaxation process. The activation energies extracted for cation dynamics for IPA_2_MA_2_Pb_3_Br_10_ sample are consistent with values reported in the literature for other lead halide hybrid perovskites comprising MA^+^ cations.^59–61^

As shown in Figure 7a, the dielectric permittivity ε′ of IPA_2_DMAPb_2_Br_7_ exhibits nearly temperature-independent behavior at low temperatures, followed by a pronounced increase upon heating. This rise becomes most pronounced at low measurement frequencies, where ionic mobility contributes significantly to the dielectric response. A small anomaly appears near ∼240 K, indicating a PT from phase **I** to phase **II**. The frequency-dependent dielectric loss ε″ presented in Figures 7b and S21b reveals a relaxation process. With increasing temperature, the relaxation peak shifts toward higher frequencies, confirming thermally activated dipolar dynamics. At low frequencies, an additional conductivity-related contribution is observed. To accurately describe the measured spectra, the dielectric response was modeled using the Havriliak–Negami (HN) function, given by

$$\varepsilon^{*}=\varepsilon_{\infty }+\frac{\Delta \varepsilon }{{\left[1+{\left(i\omega \tau \right)}^{1-\alpha }\right]}^{\beta }}+\frac{\sigma }{i\varepsilon_{0}\omega }$$

where α (0 ≤ α < 1) describes symmetric broadening, β (0 < β ≤ 1) accounts for asymmetric broadening of the relaxation-time distribution.

**Figure 7:**
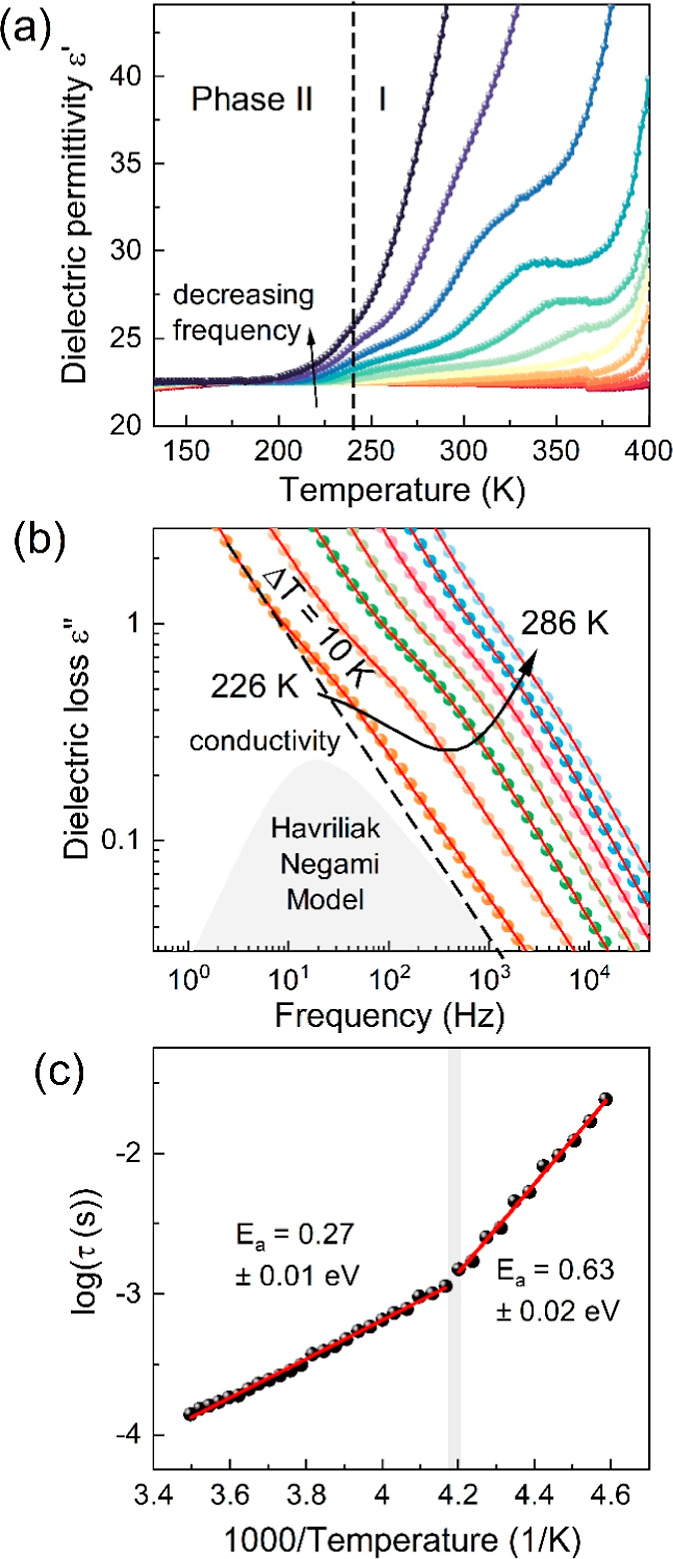
(a) Temperature dependence of the real part of the dielectric permittivity ε′ measured for IPA_2_DMAPb_2_Br_7_ at several frequencies ranging from 1 MHz (red) to 1 Hz (blue). (b) Dielectric loss spectra ε″ as a function of frequency recorded at different temperatures, with representative fitting curves obtained using the Havriliak–Negami function. (c) Inverse-temperature dependence of the relaxation time for IPA_2_DMAPb_2_Br_7_.

Figure 7c shows the Arrhenius plot of the relaxation time τ obtained for IPA_2_DMAPb_2_Br_7_ from the HN fits. Two linear regimes are identified, corresponding to distinct relaxation mechanisms. The extracted activation energies are *E*_a_ = 0.27 eV in phase **I** and *E*_a_ = 0.63 eV in phase **II**. Similar to IPA_2_MA_2_Pb_3_Br_10_, the increase in activation energy with decreasing temperature reflects a transition from a low-barrier, dynamically disordered phase to a state with increasingly restricted molecular reorientation.

### Optical Properties

To elucidate how compositional variations influence the electronic structure and, in turn, the optical response, diffuse reflectance spectra were collected at RT for all IPA-based lead bromide compounds (Figure S22). The spectra reveal two characteristic absorption bands associated with the absorption of individual host elements and excitonic bands located at approximately 463 nm (2.68 eV) for IPA_2_MA_2_Pb_3_Br_10,_ 425 nm (2.92 eV) for IPA_2_DMAPb_2_Br_7,_ and 412 nm (3.01 eV) for IPA_2_(IPA_0.77_MHy_0.23_)­Pb_2_Br_7._ The band gap energies (*E*_g_) estimated via the Kubelka–Munk function with the Tauc modification, are 2.7, 2.98, and 3.03 eV for IPA_2_MA_2_Pb_3_Br_10_, IPA_2_DMAPb_2_Br_7_, and IPA_2_(IPA_0.77_MHy_0.23_)­Pb_2_Br_7_, respectively (Figures S23–S26).^62–64^ Studies of lead halide perovskites have revealed that the band gap decreases with an increasing number of layers in the perovskite slabs (*n* value), decreasing distortion of the inorganic layers, and decreasing Pb–X bond length.^65–67^ The significantly smaller band gap of IPA_2_MA_2_Pb_3_Br_10_ (*n* = 3 compound) compared to those of the remaining perovskites (*n* = 2 compounds) is consistent with its larger *n*. Note that a similar band gap (*E*_g_ = 2.73 eV) was reported for (BDA)­(MA)_2_Pb_3_Br_10_ DJ analogue.^59^ Among the IPA-based *n* = 2 compounds, IPA_2_(IPA_0.77_MHy_0.23_)­Pb_2_Br_7_ exhibits a significantly larger band gap than IPA_2_DMAPb_2_Br_7_. Studies of (BA)_2_(A)­Pb_2_I_7_ (A = MA, FA, GA, DMA) showed the following experimental trend: *E*_*g*_,DMA > *E*_*g*_,GA > *E*_*g*_,FA > *E*_g_,MA.^39^ This trend was attributed to increased Pb–I bond length with the increasing size of the A-site cation and slightly larger octahedral distortion of the DMA analogue compared to its GA counterpart. By inspecting the crystal structures, we observe that both compounds exhibit similar Pb–Br–Pb bond angles (octahedral tilts) and Pb–Br bond lengths, however, IPA_2_(IPA_0.77_MHy_0.23_)­Pb_2_Br_7_ shows larger octahedral distortion (spread of Pb–Br bond lengths) compared to IPA_2_DMAPb_2_Br_7_, leading to the observed blue shift of the band gap. Interestingly, the band gap of IPA_2_(IPA_0.77_MHy_0.23_)­Pb_2_Br_7_ is larger than that of IPA_2_TzPb_2_Br_7_ (2.90 eV), which comprises the largest A-site cation used to date.^38^ A comparison of the crystal data shows that this behavior can be attributed to larger Pb–Br–Pb angles in IPA_2_(IPA_0.77_MHy_0.23_)­Pb_2_Br_7_ (166.6–176.3°) than IPA_2_TzPb_2_Br_7_ (175.8–176.54°).^38^

The PL of IPA_2_DMAPb_2_Br_7_ and IPA_2_(IPA_0.77_MHy_0.23_)­Pb_2_Br_7_ at 80 K is dominated by a broad band with a maximum at 611 nm (2.03 eV) and 616 nm (2.01 eV), respectively (Figures 8a and S27b,c). The corresponding fwhm values are 0.71 and 0.56 eV. Such broad PL bands have been reported for many *n* = 1 hybrid lead bromides as well as *n* = 2 IPA_2_TzPb_2_Br_7_, and they are usually attributed to self-trapped excitons (STEs) in multiple defect states of similar depth.^19,38,68–72^ However, in the case of the IPA_2_DMAPb_2_Br_7_ sample, two very narrow and barely visible bands were also observed at 416 and 423 nm, which were assigned as free (FE) and bound (BE) exciton emission, respectively. In hybrid lead halides, broadband STE-related emission occurs when a strong exciton–phonon coupling exists, which is often related to large out-of-plane tilting.^35,73^ The observation of the broadband emission for IPA_2_(IPA_0.77_MHy_0.23_)­Pb_2_Br_7_ is consistent with large octahedral distortion of this compound. As discussed above, IPA_2_DMAPb_2_Br_7_ exhibits weaker octahedral distortion than IPA_2_(IPA_0.77_MHy_0.23_)­Pb_2_Br_7_. Therefore, this compound shows both broadband and narrowband emission. A different type of emission was observed for IPA_2_MA_2_Pb_3_Br_10_. As shown in Figures 8a and S27a, the PL consists of four very narrow bands at 435, 445, 453, and 458 nm. The splitting of the excitonic band into several components has been reported for 2D lead iodides (e.g., (PEA)_2_PbI_4_ and BA_2_PbI_4_) as a result of the coupling of excited states with vibrational modes.^73–75^ Since structural data are not available for this compound, we cannot correlate its optical properties with details of the crystal structure. However, former studies of trilayered (*n* = 3) perovskites such as (BDA)­(MA)_2_Pb_3_Br_10_ and (BDA)­(MA)_2_Pb_3_Br_10_ (BDA = 1,4-butanediammonium) also showed presence of narrowband emission only. We suppose, therefore, that different behavior of IPA_2_MA_2_Pb_3_Br_10_ can be related to weaker exciton–phonon coupling in this perovskites due to decreased exciton binding energy, which is known to decrease with increasing *n*.^76^ At 80 K, IPA_2_MA_2_Pb_3_Br_10_ generates purplish-blue emission, while IPA_2_DMAPb_2_Br_7_ and IPA_2_(IPA_0.77_MHy_0.23_)­Pb_2_Br_7_ exhibit yellow and orange PL, respectively (Figure 8b).

**Figure 8:**
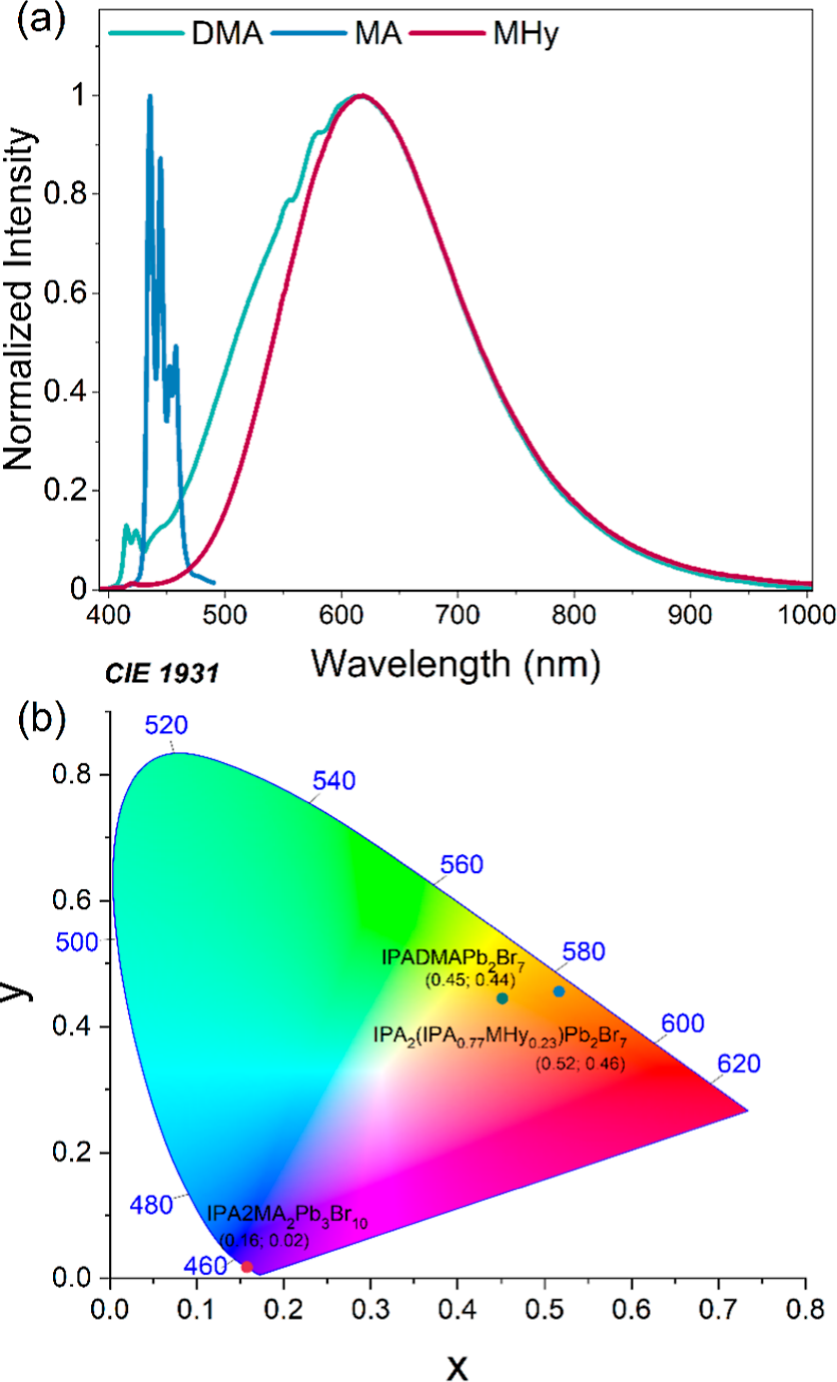
(a) Comparison of normalized emission spectra of investigated at 80 K under excitation with a beam of wavelength 360 nm and (b) CIE diagram with marked chromaticity coordinates *x*, *y*.

The PL spectra recorded as a function of temperature (from 80 to 300 K in 5 K increments) show regular thermal quenching of the PL (Figures S27 and S28) and a red shift of the FE and BE bands (Figures S29 and S30) with increasing temperature. Note that the intensity of the higher-wavelength narrow bands quickly decreases with heating; when the temperature reaches 170 K, only single bands are observed for both compounds (Figures S29 and S30). In lead halide perovskites, an increase in Pb-X bond lengths (increase of the Pb-X-Pb bond angles) leads to blue (red) shift of the narrowband PL due to reduced (enhanced) Pb s- and X p-orbital overlap.^38,39,68^ The observed redshift of the narrow PL bands therefore indicates that the temperature-induced decrease in tilting and octahedral distortion prevails over the elongation of Pb–Br bonds. It is worth noting that the FE band of IPA_2_MA_2_Pb_3_Br_10_ exhibits a sudden red shift of 9.1 nm when the temperature changes from 250 to 255 K (Figure S29), i.e., where the first-order PT takes place. Such a pronounced red shift confirms that the PT is associated with a significant decrease in octahedral tilting in the HT phase. In contrast, the STE band of IPA_2_DMAPb_2_Br_7_ shifts toward the blue with increasing temperature (Figure S30). This may result from different quenching dynamics of multiple trap states and the low thermal activation energy for quenching (*E*_a_) (60 meV) (Figure S31). A larger *E*_a_ of 80 meV is observed for the STE emission quenching of IPA_2_(IPA_0.77_MHy_0.23_)­Pb_2_Br_7_ (Figure S32), but in this case, the position of the PL band maximum remains almost unchanged in the studied temperature range (Figure S27c). Similar analysis revealed that IPA_2_MA_2_Pb_3_Br_10_ exhibits the largest *E*_a_ of 95 meV (Figure S33).

For all samples, the chromaticity of the emission evolves along a specific trajectory in the CIE 1931 color space. For IPA_2_DMAPb_2_Br_7_, the emission color shifts from yellow (*x* = 0.45, *y* = 0.44) at 80 K, through shades of white, to blue when the temperature exceeds 170 K (Figure S27b). On the other hand, although the position of the PL band maximum remains largely unchanged for IPA_2_(IPA_0.77_MHy_0.23_)­Pb_2_Br_7_ at temperatures up to nearly 200 K, the emission color changes due to the variability of the bandwidth with temperature (Figure S27c). The smallest change in chromatic coordinates is noted for IPA_2_MA_2_Pb_3_Br_10_, for which the emission color remains unchanged over the entire temperature range (Figure S27a).

## Conclusions

In summary, we report the synthesis and characterization of three new multilayered RP lead bromide perovskites IPA_2_MA_2_Pb_3_Br_10,_ IPA_2_DMAPb_2_Br_7_ and IPA_2_(IPA_0.77_MHy_0.23_)­Pb_2_Br_7_. Through a study of their crystal structures, as well as their electric, vibrational, and optical properties, we have established several key findings.

Most notably, we demonstrate that the isopropylammonium cation (IPA^+^) can play a dual role in hybrid lead halides. The synthesis of IPA_2_(IPA_0.77_MHy_0.23_)­Pb_2_Br_7_ provides the first example of a lead halide perovskite in which IPA^+^ cations reside both in the interlayer space (as a spacer) and within the perovskite cages (as a perovskitizer). This discovery opens a new avenue for the design and synthesis of other lead halides utilizing IPA^+^ as an A-site cage cation.

Furthermore, IPA_2_MA_2_Pb_3_Br_10_ represents the first example of an IPA-based lead halide perovskite exhibiting a structural PT from a centrosymmetric to a noncentrosymmetric space group, as evidenced by the emergence of strong second harmonic generation (SHG) activity below 260 K. While the fragility and small thickness of the crystals precluded the preparation of samples for ferroelectric hysteresis loop measurements with in-plane spontaneous polarization, which could confirm ferroelectric properties of this perovskite, our dielectric data unequivocally show that IPA_2_MA_2_Pb_3_Br_10_ functions as a highly stable dielectric switch.

We also analyzed the mechanisms driving the PTs in these compounds, highlighting the necessity of a multitechnique experimental approach. DSC data show that IPA_2_MA_2_Pb_3_Br_10_ undergoes two PTs, a first-order one from phase **I** to phase **II** near 260 K and a second-order PT from phase **II** to phase **III** near 235 K. Despite the difficulties in obtaining single-crystal data for IPA_2_MA_2_Pb_3_Br_10_, spectroscopic studies provided critical insights into the PT mechanism. Dielectric spectroscopy revealed that the **I** to **II** PT at 260 K is associated with a step-like anomaly, proving that this PT is associated with ordering of an organic cation. Raman spectroscopy confirmed this assignment, identifying MA^+^ as the ordering species via the sudden drop in fwhm values for MA-related bands in phase **II**. Concurrently, the blue shift of the FE emission at **I** to **II** PT and changes in the lattice modes observed in the Raman spectra proved that this transition is also coupled to significant tilting of the PbBr_6_ octahedra. IPA_2_DMAPb_2_Br_7_ undergoes only one PT near 240 K. The step-like change of the entropy and fwhm of several Raman bands related to DMA^+^ cations indicates that this PT has a first-order character and is associated with significant ordering of the DMA^+^ cations. DSC measurements of IPA_2_(IPA_0.77_MHy_0.23_)­Pb_2_Br_7_ show a very weak and broad anomaly near 250 K (Figure S6) that may result from a strongly suppressed PT. Single-crystal X-ray diffraction studies revealed that this PT leads to lowering of crystal symmetry from tetragonal (space group *P*4_2_/*ncm*) to orthorhombic (space group *Pccn*) while Raman data provide evidence that it is associated with the arrest of the dynamic disorder of the organic cations.

Finally, we demonstrate that the band gap and emission properties of these compounds are strongly modulated by the type of perovskitizer cation. By correlating structural data with optical measurements, we attribute the distinct optical properties of these compounds and previously reported IPA analogues comprising Tz^+^ cations, to variations in inorganic layer thickness (*n* value), Pb–Br bond lengths and Pb–Br–Pb bond angles.

## Supplementary Material



## Data Availability

Data for this article, including BDS data (txt); DSC data (txt); PL data (xlsx); Raman and IR data (opju); PXRD (ASC); SHG (opju) are available at https://zenodo.org/at DOI 10.5281/zenodo.18715544

## Notes

Additional experimental details including crystallographic data, Raman and IR wavenumbers, NMR data, photos of the grown crystals, X-ray powder diffraction patterns, DSC traces, NLO spectra, Raman, IR, dielectric and diffuse reflectance spectra, Tauc plots, temperature-dependence of PL band positions and intensities, and energy activations (PDF)

## References

[1] Nallusamy S., Vasanthi V., Kavinkumar T., Bhaviripudi V. R., Mangalaraja R. V., Srinivasan R., Chidhambaram N., Dhanabalan S. S., Asaithambi P., Thirumurugan A. (2025). Advances in
Optoelectronics for Environmental and Energy Sustainability. Next Energy.

[2] Yang X., Ma L., Yu M., Chen H.-H., Ji Y., Hu A., Zhong Q., Jia X., Wang Y., Zhang Y., Zhu R., Wang X., Lu C. (2023). Focus on Perovskite Emitters in Blue
Light-Emitting Diodes. Light Sci. Appl..

[3] Wang K., Park J. Y., Akriti, Dou L. (2021). Two-dimensional Halide
Perovskite
Quantum-Well Emitters: A critical Review. EcoMat.

[4] Saparov B., Mitzi D. B. (2016). Organic-Inorganic Perovskites: Structural Versatility
for Functional Materials Design. Chem. Rev..

[5] Yang T., Gao L., Lu J., Ma C., Du Y., Wang P., Ding Z., Wang S., Xu P., Liu D., Li H., Chang X., Fang J., Tian W., Yang Y., Liu S. F., Zhao K. (2023). One-Stone-for-Two-Birds
Strategy
to Attain Beyond 25% Perovskite Solar Cells. Nat. Commun..

[6] Maczka M., Ptak M., Gagor A., Zareba J. K., Liang X., Balciunas S., Semenikhin O. A., Kucheriv O. I., Gural’skiy I. A., Shova S., Walsh A., Banys J., Šimėnas M. (2023). Phase Transitions,
Dielectric Respone, and Nonlinear Optical Properties of Aziridinium
Lead Halide Perovskites. Chem. Mater..

[7] Vasileiadou E. S., Kanatzidis M. G. (2021). Structure-Property
Relationships and Idiosyncrasies
of Bulk, 2D Hybrid Lead Bromide Perovskites. Isr. J. Chem..

[8] Mao L., Stoumpos C. C., Kanatzidis M. G. (2019). Two-Dimensional
Hybrid Halide Perovskites:
Principles and Promises. J. Am. Chem. Soc..

[9] Tsai H., Nie W., Blancon J.-C., Stoumpos C. C., Soe C. M. M., Yoo J., Crochet J., Tretiak S., Even J., Sadhanala A., Azzellino G., Brenes R., Ajayan P. M., Bulović V., Stranks S. D., Friend R. H., Kanatzidis M. G., Mohite A. D. (2018). Stable Light-Emitting Diodes Using Phase-Pure Ruddlesden–Popper
Layered Perovskites. Adv. Mater..

[10] Kim J., Chu Y. H., Park J., Bang K., Yoon S., Park S., Park K., Kwon J., Kim N., Yoon K. T., Kim Y., Lee Y. S., Shin B. (2024). Spectrally
Stable Deep-Blue Light-Emitting Diodes Based on Layer Transferred
Single-Crystalline Ruddlesden–Popper Halide Perovskites. ACS Appl. Mater. Interfaces.

[11] Mączka M., Gagor A., Stefanska A., Hanuza J., Kucharska E., Zareba J. K. (2025). Divalent Methylhydrazinium_An Ultrasmall Organic Cation
for Construction of Hybrid Perovskites. Chem.
Mater..

[12] Niu T., Xue Q., Yip H. L. (2021). Advances
in Dion-Jacobson Phase Two-Dimensional Metal
Halide Perovskite Solar Cells. Nanophotonics.

[13] Liang C., Zhao D., Li Y., Li X., Peng S., Shao G., Xing G. (2018). Ruddlesden–Popper
Perovskite
for Stable Solar Cells. Energy Environ. Mater..

[14] Liang Y., Shang Q., Wei Q., Zhao L., Liu Z., Shi J., Zhong Y., Chen J., Gao Y., Li M., Liu X., Xing G., Zhang Q. (2019). Lasing from Mechanically Exfoliated
2D Homologous Ruddlesden–Popper Perovskite Engineered by Inorganic
Layer Thickness. Adv. Mater..

[15] Zhang H., Wu Y., Liao Q., Zhang Z., Liu Y., Gao Q., Liu P., Li M., Yao J., Fu H. (2018). A Two-Dimensional Ruddlesden–Popper
Perovskite Nanowire Laser Array based on Ultrafast Light-Harvesting
Quantum Wells. Angew. Chem..

[16] Li R., Wang Z., Zhu T., Ye H., Wu J., Liu X., Luo J. (2023). Stereochemically Active Lone Pair Induced Polar Tri-layered
Perovskite for Record-Performance Polarized Photodetection. Angew. Chem., Int. Ed..

[17] Min L., Tian W., Cao F., Guo J., Li L. (2021). 2D Ruddlesden–Popper
Perovskite with Ordered Phase Distribution for High-Performance Self-Powered
Photodetectors. Adv. Mater..

[18] Han X., Zheng Y., Chai S., Chen S., Xu J. (2020). 2D Organic-Inorganic
Hybrid Perovskite Materials for Nonlinear Optics. Nanophotonics.

[19] Mączka M., Zaręba J. K., Gągor A., Stefańska D., Ptak M., Roleder K., Kajewski D., Soszyński A., Fedoruk K., Sieradzki A. (2021). [Methylhydrazinium]_2_PbBr_4_, a Ferroelectric Hybrid Organic-Inorganic
Perovskite with
Multiple Nonlinear Optical Outputs. Chem. Mater..

[20] Mączka M., Zaręba J. K., Gągor A., Fedoruk-Piskorska K., Stefańska D., Drozdowski D., Ptak M., Sieradzki A. (2024). Multi-Noncentrosymmetric
Polar Order in 2D Hybrid Lead Chloride with Broadband Emission and
High-Temperature Second-Harmonic Generation Switching. ACS Appl. Mater. Interfaces.

[21] Muddam R. S., Jagadamma L. K. (2025). Ferroelectric Polarization in 2D Halide Hybrid Perovskites:
Influence on Bulk Crystals, Thin Films, and Applications. J. Mater. Chem. C.

[22] Han Y., Yue S., Cui B. B. (2021). Low-Dimensional
Metal Halide Perovskite Crystal Materials:
Structure Strategies and Luminescence Applications. Adv. Sci..

[23] Mączka M., Drozdowski D., Stefańska D., Gągor A. (2023). Zero-Dimensional
Mixed-Cation Hybrid Lead Halides with Broadband Emissions. Inorg. Chem. Front..

[24] Lun M. M., Zhou F. L., Fu D. W., Ye Q. (2022). Multi-Functional Hybrid
Perovskites with Triple-Channel Switches and Optical Properties. J. Mater. Chem. C.

[25] Fedoruk-Piskorska K., Zaręba J. K., Zelewski S. J., Gągor A., Mączka M., Drobczyński S., Sieradzki A. (2024). Three-State
Dielectric Switching within a Narrow Temperature Range in Isopropylammonium
Lead Iodide, a One-Dimensional Perovskite with Polar Phase. ACS Appl. Mater. Interfaces.

[26] Li X., Hoffman J. M., Kanatzidis M. G. (2021). The 2D Halide Perovskite Rulebook:
How the Spacer Influences Everything from the Structure to Optoelectronic
Device Efficiency. Chem. Rev..

[27] Laxmi, Srinivasan P., Kabra D. (2025). Optical and optoelectronic
properties of 2D, quasi-2D and 3D metal
halide perovskites. J. Mater. Chem. C.

[28] Gan Z., Cheng Y., Chen W., Loh K. P., Jia B., Wen X. (2021). Photophysics of 2D Organic–Inorganic Hybrid Lead Halide Perovskites:
Progress, Debates, and Challenges. Adv. Sci..

[29] Cuthriell S. A., Malliakas C. D., Kanatzidis M. G., Schaller R. D. (2023). Cyclic versus Linear
Alkylammonium Cations: Preventing Phase Transitions at Operational
Temperatures in 2D Perovskites. J. Am. Chem.
Soc..

[30] Dybała F., Kudrawiec R., Polak M. P., Herman A. P., Sieradzki A., Mączka M. (2025). Near-Bandgap Emission in [HOC_2_H_4_NH_3_]_2_PbI_4_ Perovskite Under Hydrostatic
Pressure: Emission of a Free Exciton and a Polaronic Exciton. Mater. Adv..

[31] Mączka M., Kudrawiec J., Fedoruk-Piskorska K., Stefańska D., Gągor A., Drozd M., Smółka S., Sieradzki A. (2025). Effect of
Halide Tuning on the Structural, Dielectric,
and Optical Properties of Two-Dimensional 2**-**Chloroethylammonium
Lead Halides. Inorg. Chem..

[32] Zheng H., Loh K. P. (2024). Ferroics in Hybrid Organic–Inorganic
Perovskites:
Fundamentals, Design Strategies, and Implementation. Adv. Mater..

[33] Vasileiadou E. S., Hadar I., Kepenekian M., Even J., Tu Q., Malliakas C. D., Friedrich D., Spanopoulos I., Hoffman J. M., Dravid V. P., Kanatzidis M. G. (2021). Shedding
Light on the Stability and Structure–Property Relationships
of Two-Dimensional Hybrid Lead Bromide Perovskites. Chem. Mater..

[34] Choghaei M., Schiffer M., Tyagi V., Righetto M., Du J., Buchmüller M., Brinkmann K. O., Brocks G., Görrn P., Herz L. M., Tao S., Riedl T., Olthof S. (2025). Odd-even effects
in lead-iodide-based Ruddlesden–Popper 2D perovskites. J. Mater. Chem. A.

[35] Mao L., Guo P., Kepenekian M., Spanopoulos I., He Y., Katan C., Even J., Schaller R. D., Seshadri R., Stoumpos C. C., Kanatzidis M. G. (2020). Organic
Cation Alloying on Intralayer A and Interlayer
A’ sites in 2D Hybrid Dion–Jacobson Lead Bromide Perovskites
(A’)­(A)­Pb_2_Br_7_. J. Am. Chem. Soc..

[36] Li R., Zhu T., Zhu Z.-K., Wu J., Geng Y., Luo J. (2024). Unique Perovskitizer
N-Pb Bond Switching Induced Polar Photovoltaic Effect in Trilayered
Hybrid Perovskite. Small.

[37] Simenas M., Gagor A., Banys J., Maczka M. (2024). Phase Transitions and
Dynamics in Mixed Three- and Low-Dimensional Lead Halide Perovskites. Chem. Rev..

[38] Li X., Dong H., Volonakis G., Stoumpos C. C., Even J., Katan C., Guo P., Kanatzidis M. G. (2022). Ordered
Mixed-Spacer 2D Bromide Perovskites and the Dual Role of 1,2,4-Triazolium
Cation. Chem. Mater..

[39] Li X., Fu Y., Pedesseau L., Guo P., Cuthriell S., Hadar I., Even J., Katan C., Stoumpos C. C., Schaller R. D., Harel E., Kanatzidis M. G. (2020). Negative
Pressure Engineering with Large Cage Cations in 2D Halide Perovskites
Causes Lattice Softening. J. Am. Chem. Soc..

[40] He Y., Chen Z., Fu D.-W., Hou Z., Zhang X.-M., Fu D. (2024). Room-Temperature Photoferroelectrics Semiconductor Driven by the
Interlayer Confinement Effect. Chem. Mater..

[41] Wu Z., Liu X., Ji C., Li L., Wang S., Peng Y., Tao K., Sun Z., Hong M., Luo J. (2019). Discovery of an Above-Room-Temperature
Antiferroelectric in Two-Dimensional Hybrid Perovskite. J. Am. Chem. Soc..

[42] Mao L., Morgan E. E., Li A., Kennard R. M., Hong M. J., Liu Y., Dahlman C. J., Labram J. G., Chabinyc M. L., Seshadri R. (2022). Layered Hybrid
Lead Iodide Perovskites with Short Interlayer Distances. ACS Energy Lett..

[43] Stoumpos C. C., Mao L., Malliakas C. D., Kanatzidis M. G. (2017). Structure–Band Gap Relationships
in Hexagonal Polytypes and Low-Dimensional Structures of Hybrid Tin
Iodide Perovskites. Inorg. Chem..

[44] Dolomanov O. V., Bourhis L. J., Gildea R. J., Howard J. A. K., Puschmann H. (2009). OLEX2: A Complete
Structure Solution, Refinement and Analysis Program. J. Appl. Crystallogr..

[45] Sheldrick G. M. (2015). SHELXT
– Integrated Space-Group and Crystal-Structure Determination. Acta Crystallogr. A.

[46] Sheldrick G. M. (2015). Crystal
Structure Refinement with SHELXL. Acta Crystallogr.
C.

[47] Momma K., Izumi F. (2011). VESTA 3 for Three-Dimensional
Visualization of Crystal, Volumetric
and Morphology Data. J. Appl. Crystallogr..

[48] Mączka M., Smółka S., Stefańska D., Gągor A., Zaręba J. K., Fedoruk-Piskorska K., Ptak M., Drozdowski D., Sieradzki A. (2024). Multinoncentrosymmetric Two-Dimensional Trilayered
Lead Bromide Perovskites with Methylhydrazinium Cations: Lattice Dynamics,
Phase Transitions, Dielectric Response, and Optical Properties. Chem. Mater..

[49] Fedoruk K., Drozdowski D., Mączka M., Zaręba J. K., Stefańska D., Gągor A., Sieradzki A. (2022). [Methylhydrazinium]_2_PbCl_4_, a Two-Dimensional Perovskite with Polar
and Modulated Phases. Inorg. Chem..

[50] Kurtz S. K., Perry T. T. (1968). A Powder Technique
for the Evaluation of Nonlinear
Optical Materials. J. Appl. Phys..

[51] Graja A. (1968). Production
of the Second Harmonic of Light in Ammonium Pentaborate and other
Powdered Piezoelectric Crystals. Phys. Phys.
Status Solidi B.

[52] Mączka M., Zienkiewicz J. A., Ptak M. (2022). Comparative Studies of Phonon Properties
of Three-Dimensional Hybrid Organic–Inorganic Perovskites Comprising
Methylhydrazinium, Methylammonium, and Formamidinium Cations. J. Phys. Chem. C.

[53] Mączka M., Ptak M. (2022). Temperature-Dependent Raman Studies of FAPbBr_3_ and MAPbBr_3_ Perovskites: Effect of Phase Transitions on Molecular Dynamics
and Lattice Distortion. Solids.

[54] Mączka M., Zierkiewicz W., Michalska D., Hanuza J. (2014). Vibrational Properties
and DFT Calculations of the Perovskite Metal Formate Framework of
[(CH_3_)_2_NH_2_]­(Ni­(HCOO)_3_]
System. Spectrochim. Acta, Part A.

[55] During J. R., Klaassen J. J., Darkhalil I. D., Herrebout W. A., Dom J. J. J., Van der Veken B. J. (2012). Conformational
and Structural Studies
of Isopropylamine from Temperature Dependent Raman Spectra of Xenon
Solutions and Ab Initio Calculations. J. Mol.
Struct..

[56] Staśkiewicz B., Baran J., Czapla Z. (2013). Detailed Infrared
Spectroscopic Study
of Thermal Transitions in New Hybrid [(CH_2_)_2_CHNH_3_]_4_Cd_3_Cl_10_ Crystal. J. Phys. Chem. Solids.

[57] Li H. J., Liu Y. L., Chen X. G., Gao J. X., Wang Z. X., Liao W. Q. (2019). High-Temperature
Dielectric Switching and Photoluminescence
in a Corrugated Lead Bromide Layer Hybrid Perovskite Semiconductor. Inorg. Chem..

[58] Rok M., Moskwa M., Hetmańczyk J., Hetmańczyk L., Bator G. (2022). Switchable Dielectric Constant, Structural
and Vibrational Studies
of Double Perovskite Organic–Inorganic Hybrids: (Azetidinium)_2_[KCr­(CN)_6_] and (azetidinium)_2_[KFe­(CN)_6_]. CrystEngComm.

[59] Maczka M., Stefanska D., Ptak M., Gagor A., Fedoruk-Piskorska K., Smolka S., Zareba J. K., Sieradzki A. (2025). Dion-Jacobson
Lead Bromide Perovskites With 1,4-Butanediammonium Cations: Lattice
Dynamics, Phase Transitions, Dielectric Switching and Optical Properties. Inorg. Chem..

[60] Simenas M., Balciunas S., Svirskas S., Kinka M., Ptak M., Kalendra V., Gagor A., Szewczyk D., Sieradzki A., Grigalaitis R., Walsh A., Maczka M., Banys J. (2021). Phase Diagram
and Cation Dynamics of Mixed MA_1–*x*
_FA*x*PbBr_3_ Hybrid Perovskites. Chem. Mater..

[61] Simenas M., Balciunas S., Gagor A., Pieniazek A., Tolborg K., Kinka M., Klimavicius V., Svirskas S., Kalendra V., Ptak M., Szewczyk D., Herman A. P., Kudrawiec R., Sieradzki A., Grigalaitis R., Walsh A., Maczka M., Banys J. (2022). Mixology of
MA_1–x_EA_x_PbI_3_ Hybrid Perovskites:
Phase Transitions, Cation Dynamics, and Photoluminescence. Chem. Mater..

[62] Kubelka P., Munk F. A. (1931). Contribution to
the Optics of Pigments. Z. Technol. Phys..

[63] Tauc J., Grigorovici R., Vancu A. (1966). Optical Properties And Electronic
Structure of Amorphous Germanium. Phys. Status
Solidi B.

[64] Makuła P., Pacia M., Macyk W. (2018). How to Correctly
Determine Band Gap
Energy of Modified Semiconductor Photocatalysts Based on UV-Vis Spectra. J. Phys. Chem. Lett..

[65] Spanopoulos I., Hadar I., Ke W., Tu Q., Chen M., Tsai H., He Y., Shekhawat G., Dravid V. P., Wasielewski M. R., Mohite A. D., Stoumpos C. C., Kanatzidis M. G. (2019). Uniaxial Expansion of the 2D Ruddlesden-Popper Perovskite
Family for Improved Environmental Stability. J. Am. Chem. Soc..

[66] Zhang Y., Abdi-Jalebi M., Larson B. W., Zhang F. (2024). What Matters for the
Charge Transport of 2D Perovskites?. Adv. Mater..

[67] Song Y., Zhou Y., Chen C., Fan K., Wang Z., Guo Y., Chen Z., Mao L., Yin J., Chow P. C. Y. (2025). Structure-Emission
Property Relationship of Bilayer 2D Hybrid Perovskites. J. Am. Chem. Soc..

[68] Smith M. D., Jaffe A., Dohner E. R., Lindenberg A. M., Karunadasa H. I. (2017). Structural Origin of Broadband Emission from Layered
Pb-Br Hybrid Perovskites. Chem. Sci..

[69] Lekina Y., Shen Z. H. (2019). Excitonic States and Structural Stability in Two-Dimensional
Hybrid Organic-Inorganic Perovskites. J. Sci:
Adv. Mater. Devices.

[70] Du K. Z., Tu Q., Zhang X., Han Q., Liu J., Zauscher S., Mitzi D. B. (2017). Two-Dimensional
Lead­(II) Halide-Based Hybrid Perovskites
Templated by Acene Alkylamines: Crystal Structures, Optical Properties,
and Piezoelectricity. Inorg. Chem..

[71] Koegel A. A., Mozur E. M., Oswald I. W. H., Jalarvo N. H., Prisk T. R., Tyagi M., Neilson J. R. (2022). Correlating
Broadband Photoluminescence
with Structural Dynamics in Layered Hybrid Halide Perovskites. J. Am. Chem. Soc..

[72] Maczka M., Sobczak S., Roszak K., Vasconcelos D. L. M., Dybała F., Herman A. P., Kudrawiec R., Katrusiak A., Freire P. T. C. (2025). Pressure-Induced Detrapping from
Self-Trapped Excitons to Free Excitons toward Enhanced Emission and
Piezochromism in Ruddlesden–Popper (110)-Oriented Perovskites. ACS Appl. Mater. Interfaces.

[73] Smith M. D., Connor B. A., Karunadasa H. I. (2019). Tuning the Luminescence of Layered
Halide Perovskites. Chem. Rev..

[74] Straus D. B., Hurtado Parra S., Iotov N., Gebhardt J., Rappe A. M., Subotnik J. E., Kikkawa J. M., Kagan C. R. (2016). Direct Observation
of Electron–Phonon Coupling and Slow Vibrational Relaxation
in Organic–Inorganic Hybrid Perovskites. J. Am. Chem. Soc..

[75] Ni L., Huynh U., Cheminal A., Thomas T. H., Shivanna R., Hinrichsen T. F., Ahmad S., Sadhanala A., Rao A. (2017). Real-Time Observation
of Exciton–Phonon Coupling Dynamics
in Self-Assembled Hybrid Perovskite Quantum Wells. ACS Nano.

[76] Gelvez-Rueda M. C., Hutter E. M., Cao D. H., Renaud N., Stoumpos C. C., Hupp J. T., Savenije T. J., Kanatzidis M. G., Grozema F. C. (2017). Interconversion between Free Charges
and Bound Excitons
in 2D Hybrid Lead Halide Perovskites. J. Phys.
Chem. C.

