# Supplementary material for: Isopropylammonium as a Dual-Role Cation: Spacer and Perovskitizer in Ruddlesden–Popper Lead Bromides
Source: Inorg Chem. 2026 Apr 29;65(18):10262–75. doi: 10.1021/acs.inorgchem.6c01079 (PMC13308882; doi:10.1021/acs.inorgchem.6c01079)
Supplement: Supplementary file 1 [file ic6c01079_si_001.pdf]

Supporting Information for

Isopropylammonium as a Dual-Role Cation: Spacer and Perovskitizer in Ruddlesden-Popper Lead Bromides

Mirosław Mączka,*[a] Anna Gągor,[a] Barbara Popanda,[a] Maciej Ptak,[a] Dagmara Stefańska,[a] Katarzyna Fedoruk-Piskorska,[b,c] Jan K. Zaręba,[d] Adam Sieradzki[b]

[a]*Institute of Low Temperature and Structural Research, Polish Academy of Sciences, Okólna 2, 50-422 Wrocław, Poland*

[b]*Department of Experimental Physics, Wrocław University of Science and Technology, Wybrzeże Wyspiańskiego 27, 50-370 Wrocław, Poland*

[c]*August Chelkowski Institute of Physics, University of Silesia in Katowice, 41-500 Chorzow, Poland*

[d]*Institute of Advanced Materials, Faculty of Chemistry, Wrocław University of Science and Technology, Wybrzeże Wyspiańskiego 27, 50-370 Wrocław, Poland*

e-mail: m.maczka@intibs.pl

Table S1. Crystal data, data collection and refinement results for $IPA_2DMAPb_2Br_7$ and $IPA_2(MHy_{0.23}IPA_{0.77})Pb_2Br_7$.

	$IPA_2DMAPb_2Br_7$	$IPA_2(MHy_{0.23}IPA_{0.77})Pb_2Br_7$	
		**(I)**	**(II)**
Chemical formula	$Br_7Pb_2\cdot 2(C_3H_{10}N)\cdot C_2NH_8$	$Br_7Pb_2\cdot 2(C_3H_{10}N)\cdot 0.23(C_2NH_8)$	$0.77(C_3H_{10}N)$
M_r	1140.08	1150.80	1150.46
Crystal system	Orthorhombic	Tetragonal	Orthorhombic
space group	*Cmce*	*P*4$_2$/*ncm*	*Pccn*
Temperature (K)	270	280	180
a, *b*, *c* (Å)	8.4750 (5), 34.933 (3), 8.5160 (5)	8.4763 (2), 34.893 (2)	8.4302 (3), 8.4342 (3), 34.418 (2)
V (Å^3)	2521.2 (3)	2506.93 (19)	2447.22 (19)
Z	4	4	4
μ (mm^{-1})	24.43	24.57	25.17
Crystal size (mm)	0.15 × 0.12 × 0.03	0.18 × 0.14 × 0.04	0.18 × 0.14 × 0.04
T_{min}, T_{max}	0.075, 1.000	0.431, 1.000	0.380, 1.000
No. of measured, ind. and observed [$I > 2\sigma(I)$] ref.	10194, 1724, 893	22502, 1319, 825	27217, 3182, 1627
R_{int}	0.081	0.063	0.065
$(\sin \theta/\lambda)_{max}$ (Å^{-1})	0.693	0.610	0.692
$R[F^2 > 2\sigma(F^2)]$, $wR(F^2)$, S	0.061, 0.199, 1.02	0.073, 0.149, 1.12	0.094, 0.211, 1.03
No. of reflections	1724	1319	3182
No. of parameters	76	76	100
No. of restraints	21	16	35
$\Delta\rho_{max}$, $\Delta\rho_{min}$ (e Å^{-3})	1.62, -1.28	1.28, -0.92	2.44, -2.30

Table S2. RT FT-IR and FT-Raman wavenumbers (in cm^{-1}) of powdered $IPA_2MA_2Pb_3Br_{10}$ sample as well as Raman wavenumbers of a single crystal of $IPA_2MA_2Pb_3Br_{10}$ measured at 360 and 80 K together with the proposed assignment.[a] Modes corresponding to MA^+ are in bold.

FT-IR 300 K	FT-Raman 300 K	Raman 320 K	Raman 250 K	Raman 80 K	assignment
3178s	3167vw,b				$\nu_{as}(NH_3)$+**$\nu_{as}(NH_3)$**
3126vs	3095vw,b				$\nu_s(NH_3)$+**$\nu_s(NH_3)$**
3036sh	3034vw				$\nu_{as}(CH_3)$+**$\nu_{as}(CH_3)$**
2980m	2983s				$\nu_s(CH_3)$
	**2966s**				**$\nu_s(CH_3)$**
2934w	2941s				$\nu_s(CH_3)$
2922w	2916w				combination
	2900w				combination
	2881w				combination
**1594sh**				**1596w**	**$\delta_{as}(NH_3)$**
1579s	1579m	1578w	1581w	1588w	$\delta_{as}(NH_3)$
			1570w	1573w	$\delta_{as}(NH_3)$
**1476s**	**1479m**	**1476w**	**1482w**	**1494w**+1487w	**$\delta_s(NH_3)$**+$\delta_s(NH_3)$
1465sh	1463s	1462vw	1466w	1474vw+1470w	$\delta_{as}(CH_3)$
				**1459w**	**$\delta_{as}(CH_3)$**
	1447w	1449sh	1445w	1443w	$\delta_{as}(CH_3)$
**1424vw**	**1425vw**		**1425vw**	**1422w**	**$\delta_s(CH_3)$**
1398m	1399w	1397vw	1397vw	1398vw+1393vw	$\delta_s(CH_3)$
1382m	1384w	1382vw	1383vw	1382vw	$\delta_s(CH_3)$
1371sh	1371w		1372vw	1364vw	$\delta_s(CH_3)$
1347w	1348w	1348vw	1348vw	1347vw	$\delta(CH)$
**1252w**	**1249w**	**1248vw**	**1252vw**	**1255w**	**$\delta(CN)$**
1203m	1206m	1204w	1204w	1214w+1199m	$\rho(CH_3)$
1156m	1158w		1160vw	1161vw	$\rho(CH_3)$
976m	977sh			989w+979w	$\nu(CN)$

**969sh**	**970m**	**968w**	**971m**	**973s**	**ν(CN)**
955vw	956w			963vw	ρ(CH_3)
934sh	925m		925w	930m	ρ(CH_3)
**918s**	**917sh**	**916sh**	**917sh**	**919s**	**ρ(NH_3)**
794w	795s	795vw	795w	801w	ν_s(CCC)
455vw	460w	456w	455m	454s	δ(CCN)
	394w	390w	390m	383s	γ(CCN)
	379w	377w			δ_s(CCC)
	**313sh**	**308m**	**304m**	**298m**	MA cage mode
	273sh		273sh	276w	τ(CH_3)
				238w	τ(CH_3)
	153m	137w,b	135w,b	142w+127vw	L+ T'+ Pb-Br stretch
			112w,b	114w	L+ T'+ Pb-Br stretch
	92sh		93w,b	98w	L+ T'+ Pb-Br stretch
				86vw+74w	Pb-Br bend
		55sh	61sh	64w+59w	Pb-Br bend
				52vs+47vs	Pb-Br bend
			45vs	43m	Pb-Br bend
		31vs	36vs	37vs	L($PbBr_6$)
				31w	L($PbBr_6$)
		22vs	22sh	27w	L($PbBr_6$)
			17w	15w	L($PbBr_6$)

[a] key: vs, very strong; s, strong; m, medium; w, weak; vw, very weak; sh, shoulder; ν, stretching; δ, in-plane bending; γ, out-of-plane bending; ρ, rocking; τ, twist; L, librational mode; T', translational mode of organic cation.

Table S3. RT FT-IR and FT-Raman wavenumbers (in cm^{-1}) of powdered $IPA_2DMAPb_2Br_7$ sample as well as Raman wavenumbers of a single crystal of $IPA_2DMAPb_2Br_7$ measured at 360 and 80 K together with the proposed assignment.[a] Modes corresponding to DMA^+ are in bold.

FT-IR 300 K	FT-Raman 300 K	Raman 300 K	Raman 80 K	assignment
3164sh	3162sh			$\nu_{as}(NH_3)$+**$\nu_{as}(NH_2)$**
3120s+3077s				$\nu_s(NH_3)$+**$\nu_s(NH_2)$**
	**3039w**			**$\nu_{as}(CH_3)$**
3032sh	3025w			$\nu_{as}(CH_3)$
2978vs	2982s			$\nu_s(CH_3)$
**2961vw**	**2961s**			**$\nu_s(CH_3)$**
2938m	2940s			$\nu_s(CH_3)$
2912m	2916w			combination
	2902w			combination
2882sh	2880w			combination
2841m				combination
**1630w**				**$\delta(NH_2)$**
1575s	1582m	1580w	1596vw+1583s	$\delta_{as}(NH_3)$
	1574m	1571sh	1569vw+1558m	$\delta_{as}(NH_3)$
1474s+1463s	1478sh	1473sh	1475vw	$\delta_s(NH_3)$+**$\delta_{as}(CH_3)$**
	1461s	1459w	1469s+1461s	$\delta_{as}(CH_3)$+**$\delta_{as}(CH_3)$**
1447sh	1446w	1446w	1445vw+1432w	$\delta_{as}(CH_3)$+**$\delta_s(CH_3)$**
	1401w		1406w	$\delta_s(CH_3)$
1396s		1397vw	1400vw+1392w	$\delta_s(CH_3)$
1380s	1384w		1382w	$\delta_s(CH_3)$
1366sh	1369w		1368vw	$\delta_s(CH_3)$
1343w	1348w	1350vw	1358w+1347w	$\delta(CH)$
			**1251w**	**$\rho(CH_3)$**
	1233vw		1235vw	$\rho(CH_3)$
1202s	1205m	1204w	1211w+1201s	$\rho(CH_3)$

1157m	1159w		1160vw	$\rho(CH_3)$
**1073w**	**1079w**	**1074w**	**1078w**	**$\rho(CH_3)$**
			1025w	$\nu_s(CN)$
**1011m**	**1014w**	**1011w**	**1015m**	**$\nu_{as}(CNC)$**
984sh	987sh		996w+989w	$\nu(CN)$
976m	978m	978vw	981m	$\nu(CN)$
956w	958w	957w	963w+966w	$\rho(CH_3$
932w				$\rho(CH_3)$
925w	925m	925m	938w+929m	$\rho(CH_3)$
**884w**	**885m**	**885m**	**889s**	**$\nu_s(CNC)$**
**816m**			**822m**	**$\rho(NH_2)$**
795m	796s	796vw	803m+799m	$\nu_s(CCC)$
455m	459w	456w	456s+450sh	$\delta(CCN)$
	**407sh**	**406vw**	**406w**	**$\delta_{as}(CCN)$**
	393w	392w	385s+390sh	$\gamma(CCN)$
	380w	379vw		$\delta_s(CCC)$
			302m+274w	$\tau(CH_3)$
	260w	259w	261m+249m	$\tau(CH_3)$
			222s	L+ T’+$\tau(CH_3)$
	155m	154sh		L+ T’+ Pb-Br stretch
		122m,b	132s	L+ T’+ Pb-Br stretch
	93sh	92w,b	99w	L+ T’+ Pb-Br stretch
			78w+84w	Pb-Br bend
		49s	54s+47s	Pb-Br bend
		34s	35s	$L(PbBr_6)$
			27sh	$L(PbBr_6)$
			22sh	$L(PbBr_6)$

[a] key: vs, very strong; s, strong; m, medium; w, weak; vw, very weak; sh, shoulder; ν, stretching; δ, in-plane bending; γ, out-of-plane bending; ρ, rocking; τ, twist; L, librational mode; T’, translational mode of organic cation.

Table S4. RT FT-IR and FT-Raman wavenumbers (in cm^{-1}) of powdered $IPA_2(IPA_{0.77}MHy_{0.23})Pb_2Br_7$ sample as well as Raman wavenumbers of a single crystal of $IPA_2(IPA_{0.77}MHy_{0.23})Pb_2Br_7$ measured at 300 and 80 K together with the proposed assignment.[a] Modes corresponding to MHy^+ are in bold.

FT-IR 300 K	FT-Raman 300 K	Raman 380 K	Raman 80 K	assignment
		**3255w**	**3242w**	$\boldsymbol{\nu_{as}(NH_2)}$
3170m		3173m	3177m	$\nu_{as}(NH_3)$+$\boldsymbol{\nu_s(NH_2)}$
3113s			3124m	$\nu_s(NH_3)$+$\boldsymbol{\nu_{as}(NH_2^+)}$
3078s		3080m	3077m	$\nu_s(NH_3)$+$\boldsymbol{\nu_s(NH_2^+)}$
3033s	3026vw	3030m	3032m	$\nu_{as}(CH_3)$+$\boldsymbol{\nu_{as}(CH_3)}$
2979s	2985s	2982s	2986s	$\nu_s(CH_3)$
	**2951sh**	**2953m**	**2947s**	$\boldsymbol{\nu_s(CH_3)}$
	2939s	2938s	2941sh	$\nu_s(CH_3)$
2926m	2918w	2918sh	2921w	combination
	2902w	2902sh	2901sh	combination
	2879w	2880w	2879w	combination
**1590sh**			**1598sh**	$\boldsymbol{\delta(NH_2)}$
1576s	1574m	1576s	1582s+1567s	$\delta_{as}(NH_3)$+$\boldsymbol{\delta(NH_2^+)}$
1475s		1476m	1473sh+1467s	$\delta_s(NH_3)$
1467s	1461s	1453m	1445m	$\delta_{as}(CH_3)$+$\boldsymbol{\delta_{as}(CH_3)}$
1447sh	1446w			$\delta_{as}(CH_3)$+$\boldsymbol{\delta_{as}(CH_3)}$
			**1409vw**	$\boldsymbol{\delta_s(CH_3)}$
1396s	1400w	1398w	1394w	$\delta_s(CH_3)$
1382m	1385w	1382vw	1382w	$\delta_s(CH_3)$
1367sh	1372w	1371w	1365w	$\delta_s(CH_3)$
1345w	1349w	1348vw	1347w	$\delta(CH)$
			**1297w,b**	$\boldsymbol{\tau(NH_2^+)}$
1203s	1203m	1202w	1214sh+1204m	$\rho(CH_3)$
1156m	1156w	1153w	1152w	$\rho(CH_3)$

**1081w**	**1080sh**		**1103w**	**$\rho(CH_3)+\tau(NH_2)$**
**1071w**	**1074w,b**		**1076w**	**$\rho(CH_3)+\tau(NH_2)$**
985sh+975m	978m	977m	992m+979m	$\nu(CN)$
956w	957w	960vw	961vw	$\rho(CH_3)$
935w	938vw	937vw	937sh	$\rho(CH_3)$
925w	925m	924w	928m	$\rho(CH_3)$
**868w**	**879w+862w**		**891vw+867w**	**$\nu_s(CNN)$**
**859w+845w**			**844w**	**$\rho(NH_2^+)$**
794w	796s	795m	800m	$\nu_s(CCC)$
456m	459w	458w	455m	$\delta(CCN)$
	**436w**		**433vw**	**$\delta(CNN)$**
	393w	391w	397sh+391w	$\gamma(CCN)$
	378w	377w	374w	$\delta_s(CCC)$
		296m	290m	MHy-cage mode
	256sh	256sh	261sh	$\tau(CH_3)$
			228w	$\tau(CH_3)$
	153m	150sh	151sh	L+ T'+ Pb-Br stretch
			135w	L+ T'+ Pb-Br stretch
	94sh		99vw	L+ T'+ Pb-Br stretch
		47vs	53vs+46sh	Pb-Br bend
		31vs	29sh	$L(PbBr_6)$

[a] key: vs, very strong; s, strong; m, medium; w, weak; vw, very weak; sh, shoulder; ν, stretching; δ, in-plane bending; γ, out-of-plane bending; ρ, rocking; τ, twist; L, librational mode; T', translational mode of organic cation.

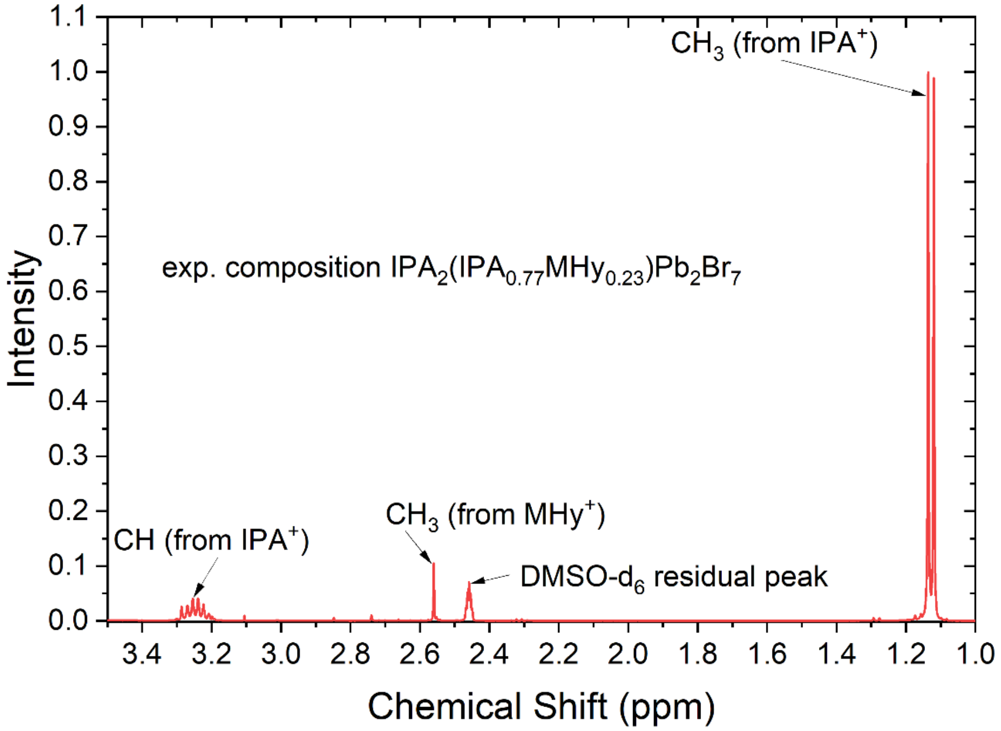

Figure S1. Room temperature ^{1}H NMR spectra of $IPA_2(IPA_{0.77}MHy_{0.23})Pb_2Br_7$ in DMSO-d_6.

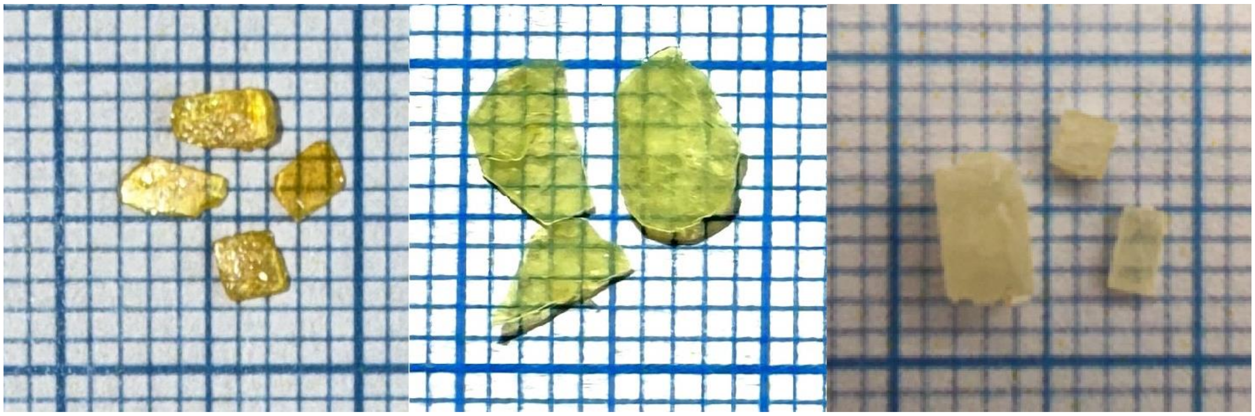

Figure S2. Photos of the $IPA_2MA_2Pb_3Br_{10}$, $IPA_2DMAPb_2Br_7$ and $IPA_2(IPA_{0.77}MHy_{0.23})Pb_2Br_7$ crystals (from left to right).

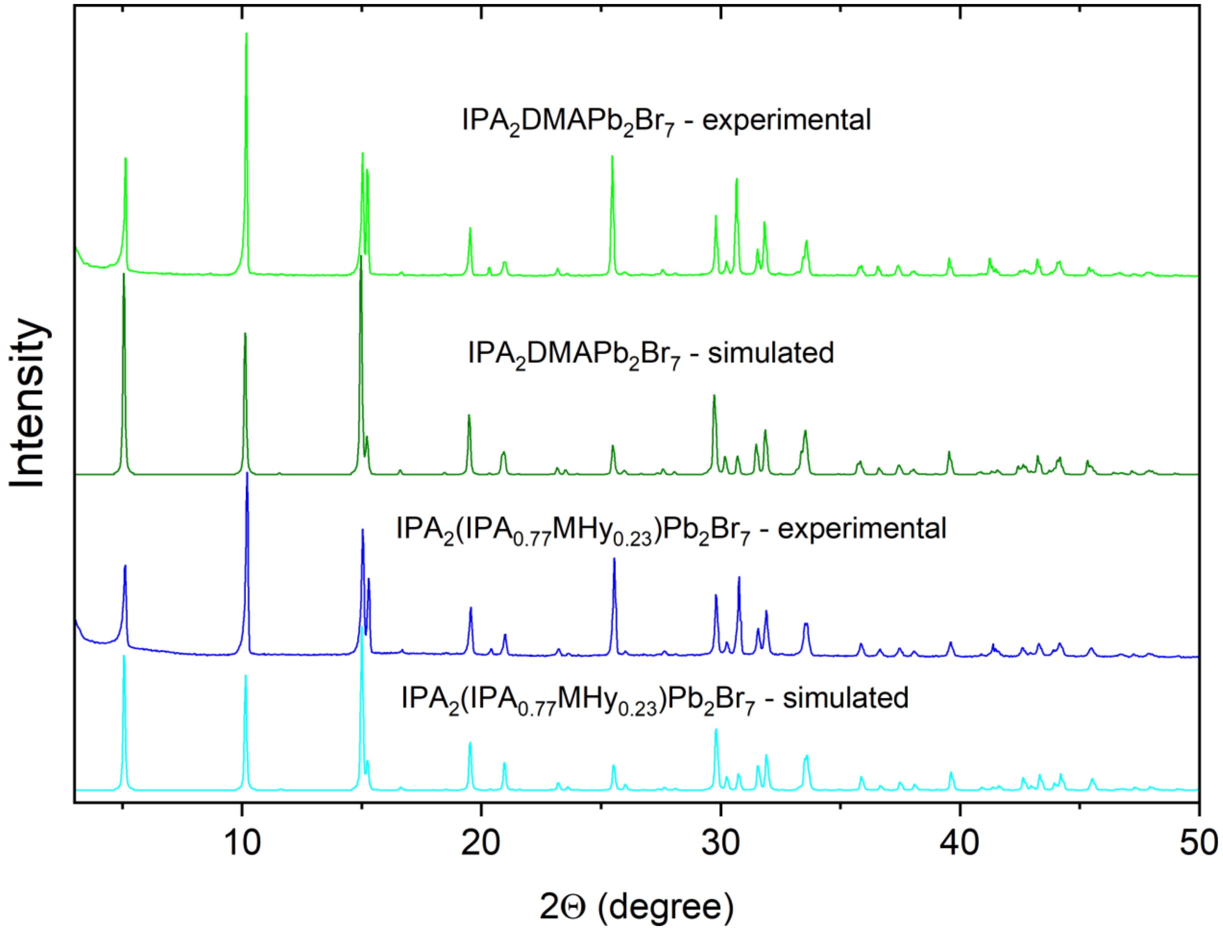

Figure S3. Experimental and simulated powder diffraction patterns of $IPA_2DMAPb_2Br_7$ and $IPA_2(IPA_{0.77}MHy_{0.23})Pb_2Br_7$.

(b)

0kl, 120 K

1kl , 120 K

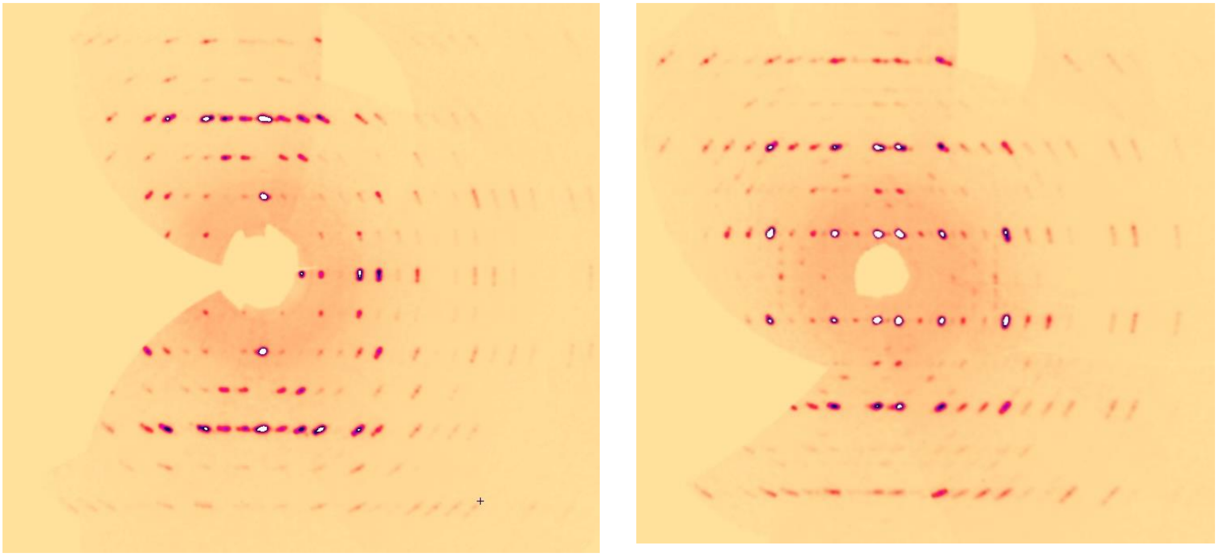

Figure S4. Reciprocal space reconstructions of $IPA_2DMAPb_2Br_7$ at 120 K from single-crystal diffraction.

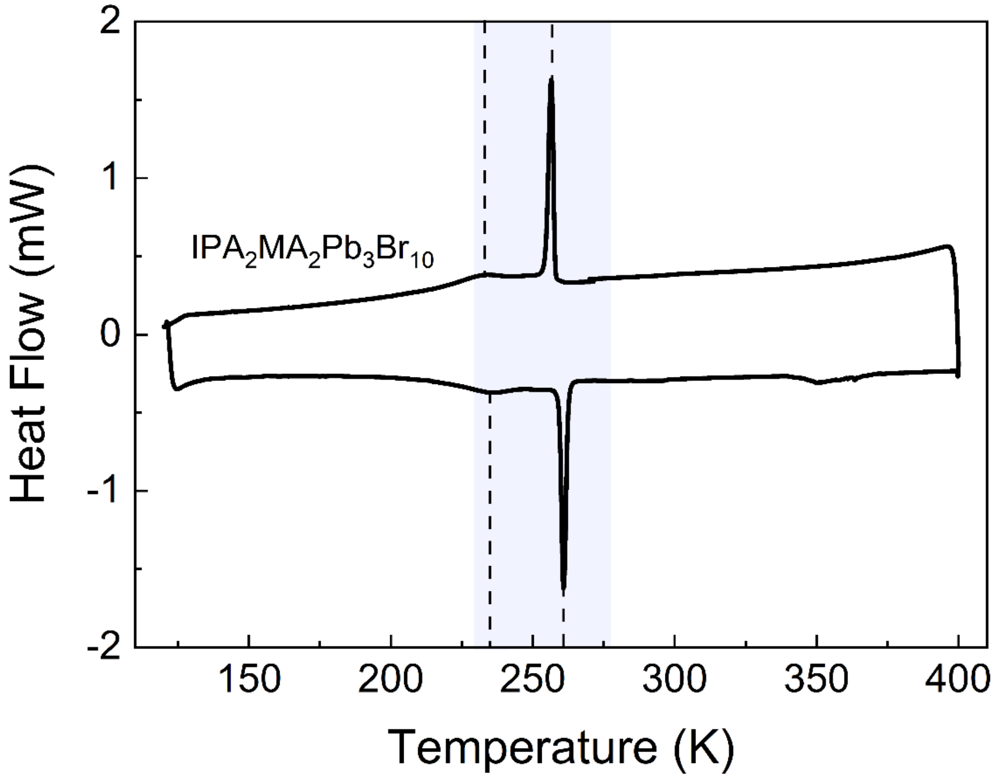

Figure S5. DSC traces for $IPA_2MA_2Pb_3Br_{10}$.

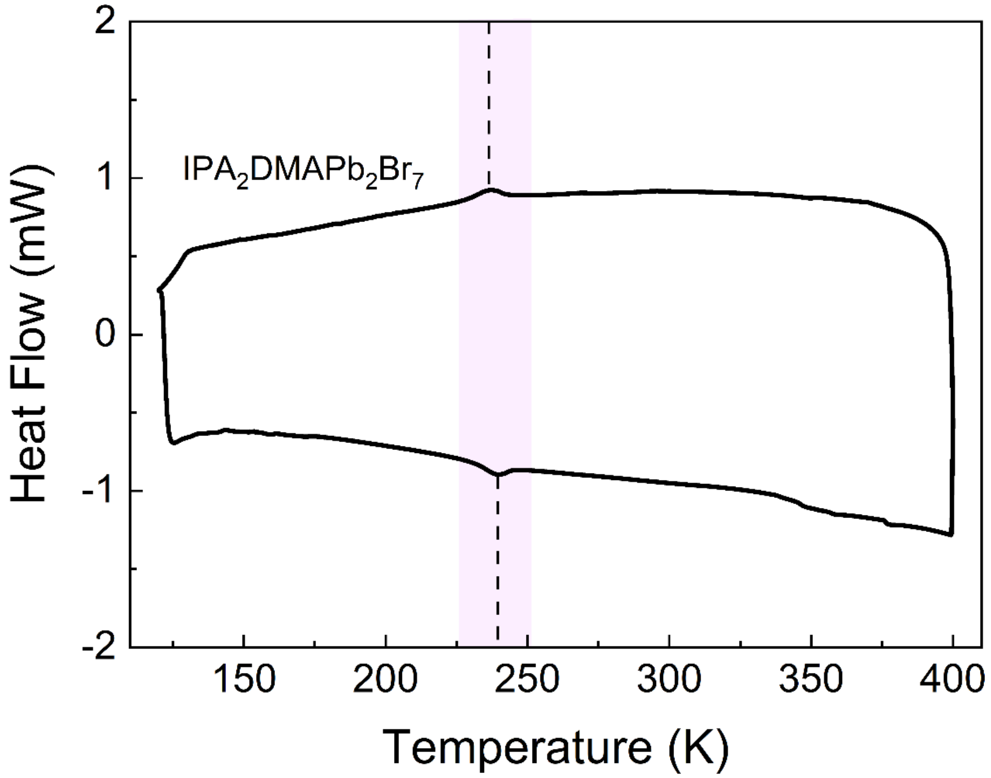

Figure S6. DSC traces for $IPA_2DMAPb_2Br_7$.

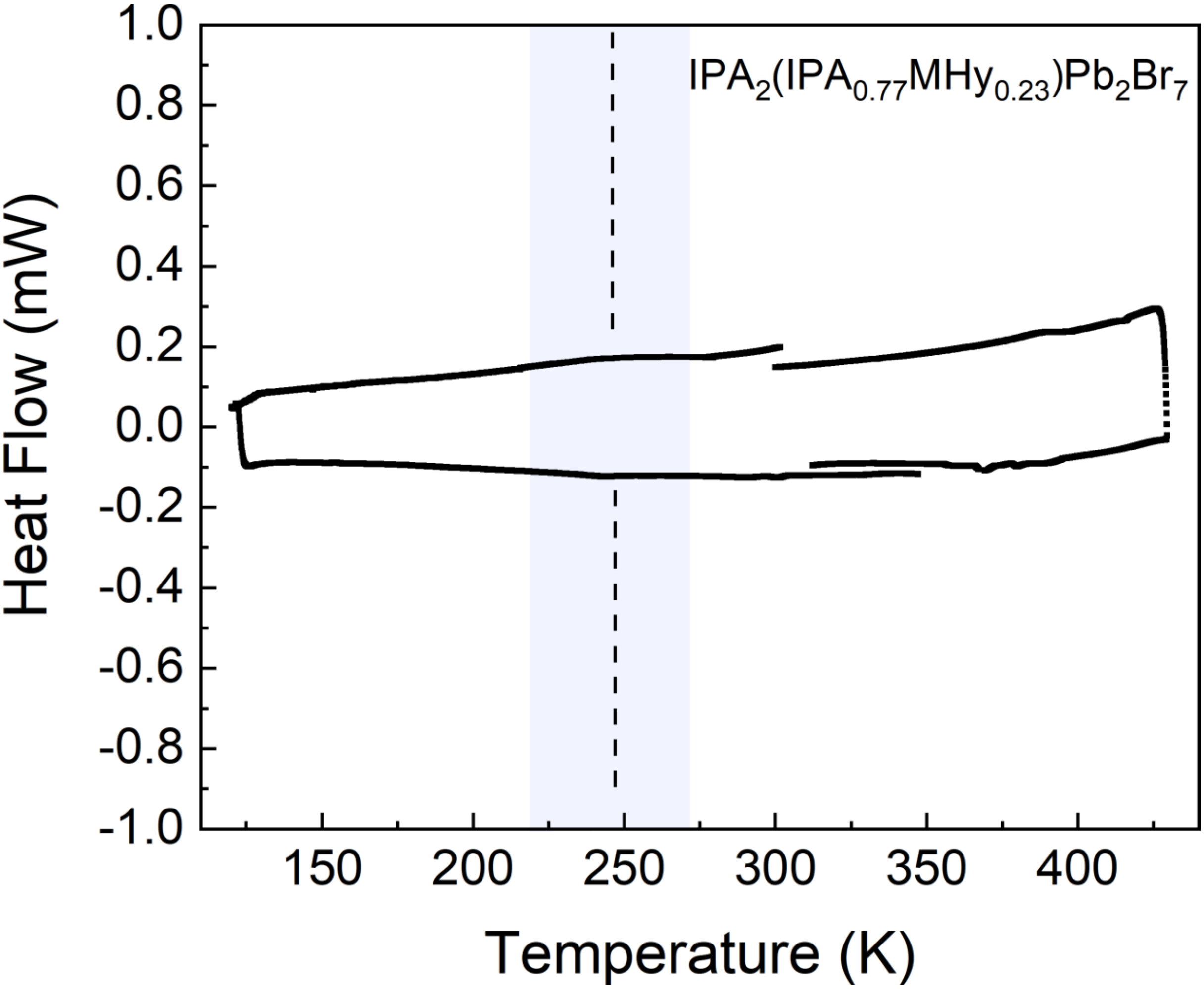

Figure S7. DSC traces for $IPA_2(IPA_{0.77}MHy_{0.23})Pb_2Br_7$.

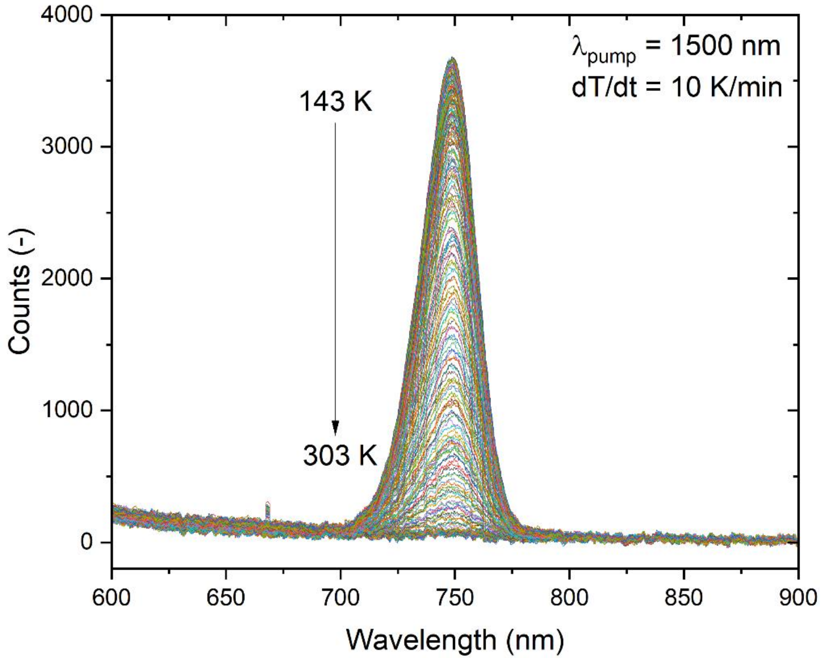

Figure S8. Experimental spectra of SHG response for $IPA_2MA_2Pb_3Br_{10}$ registered during heating run (143 K to 303 K).

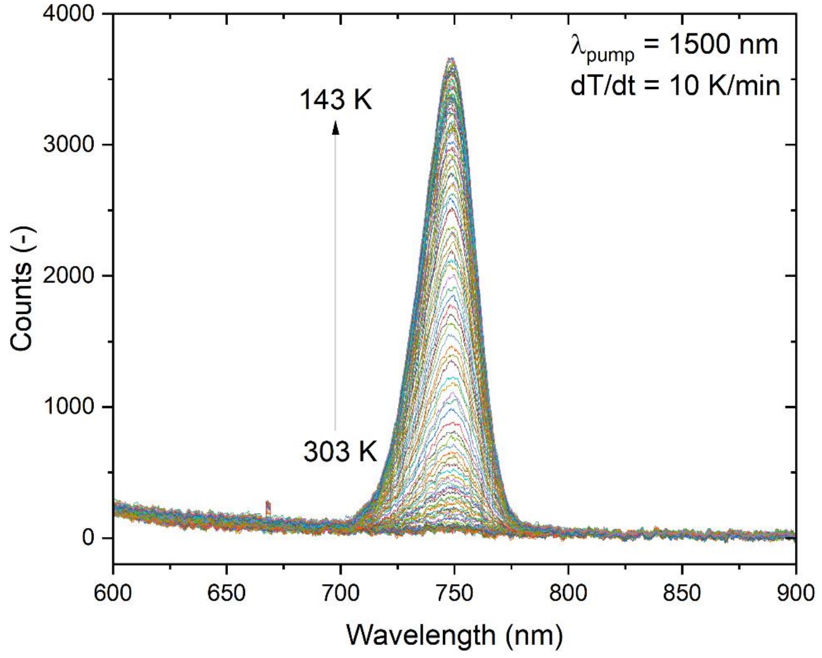

Figure S9. Experimental spectra of SHG response for $IPA_2MA_2Pb_3Br_{10}$ registered during cooling run (303 K to 143 K).

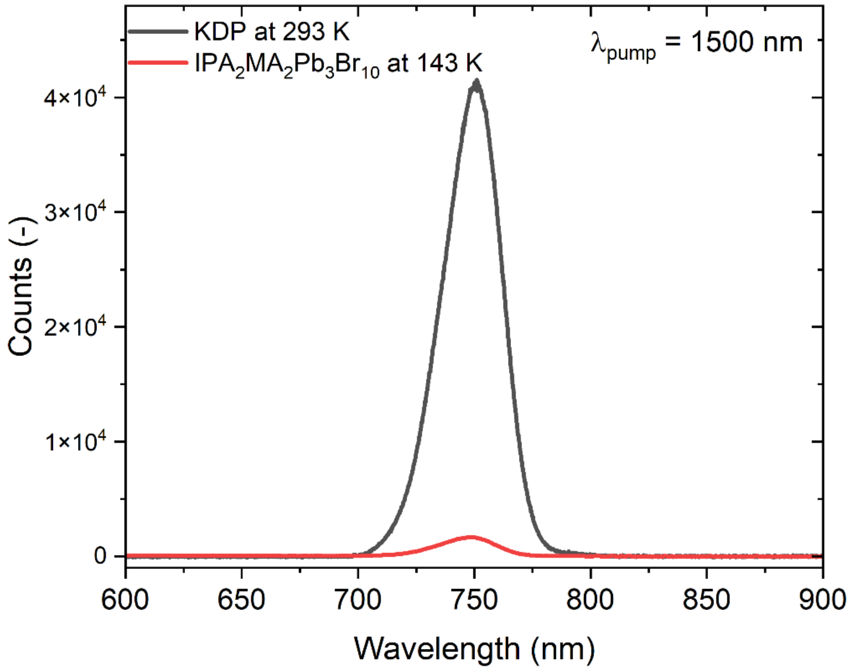

Figure S10. The SHG signal of $IPA_2MA_2Pb_3Br_{10}$ (collected at 253K) overlaid with that of KDP (293K) of the same particle size. Data are normalized to the same signal collection time.

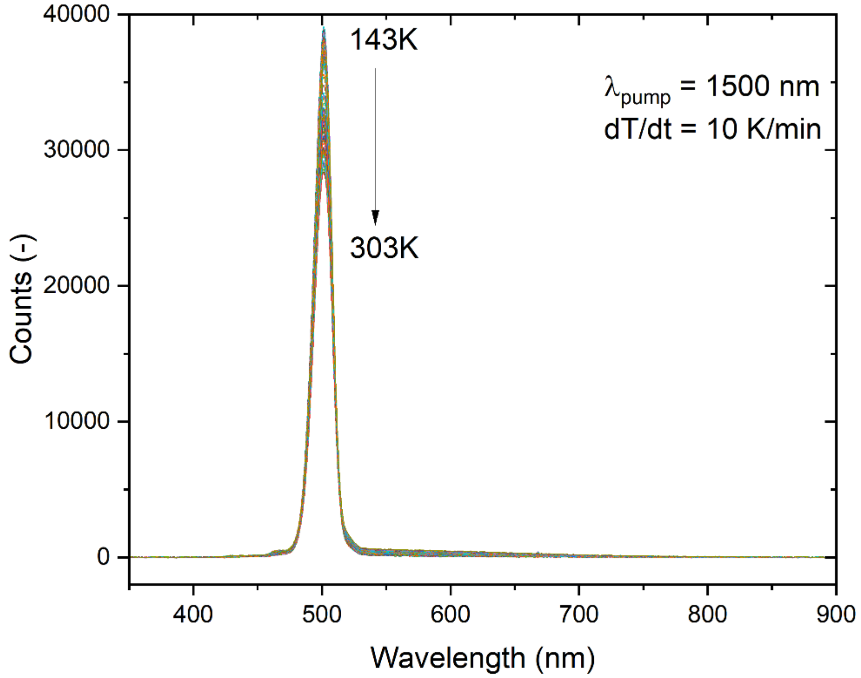

Figure S11. Experimental spectra of NLO responses for $IPA_2DMAPb_2Br_7$ registered during heating run (143 K to 303 K). Note there is no SHG signal at 750 nm.

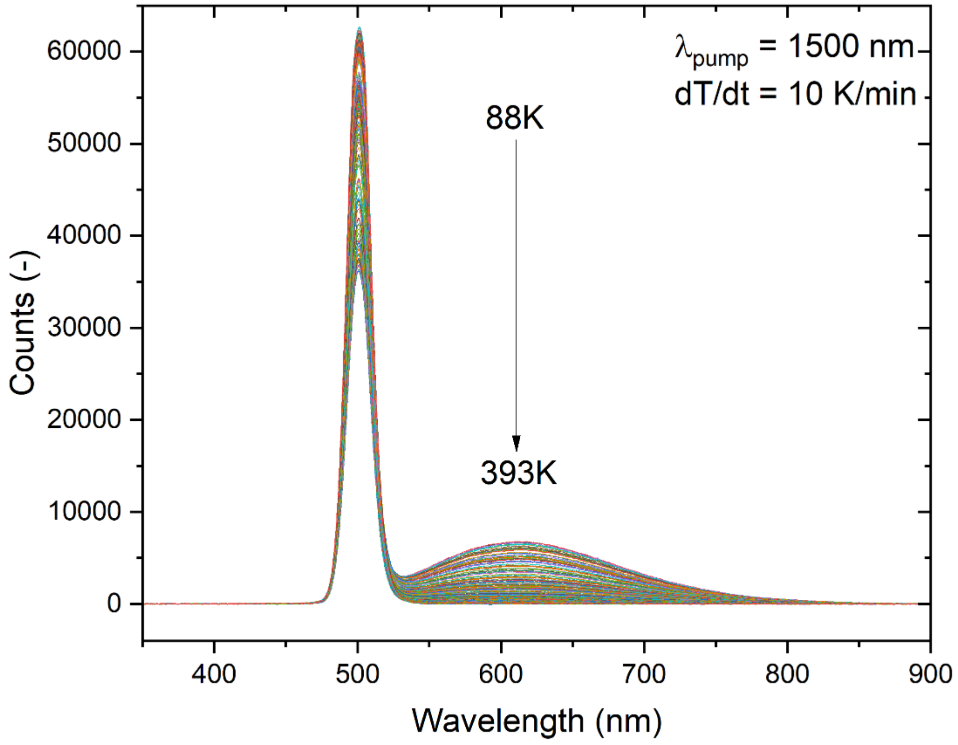

Figure S12. Experimental spectra of NLO responses for $IPA_2(IPA_{0.77}MHy_{0.23})Pb_2Br_7$ registered during heating run (88 K to 393 K). Note there is no SHG signal at 750 nm.

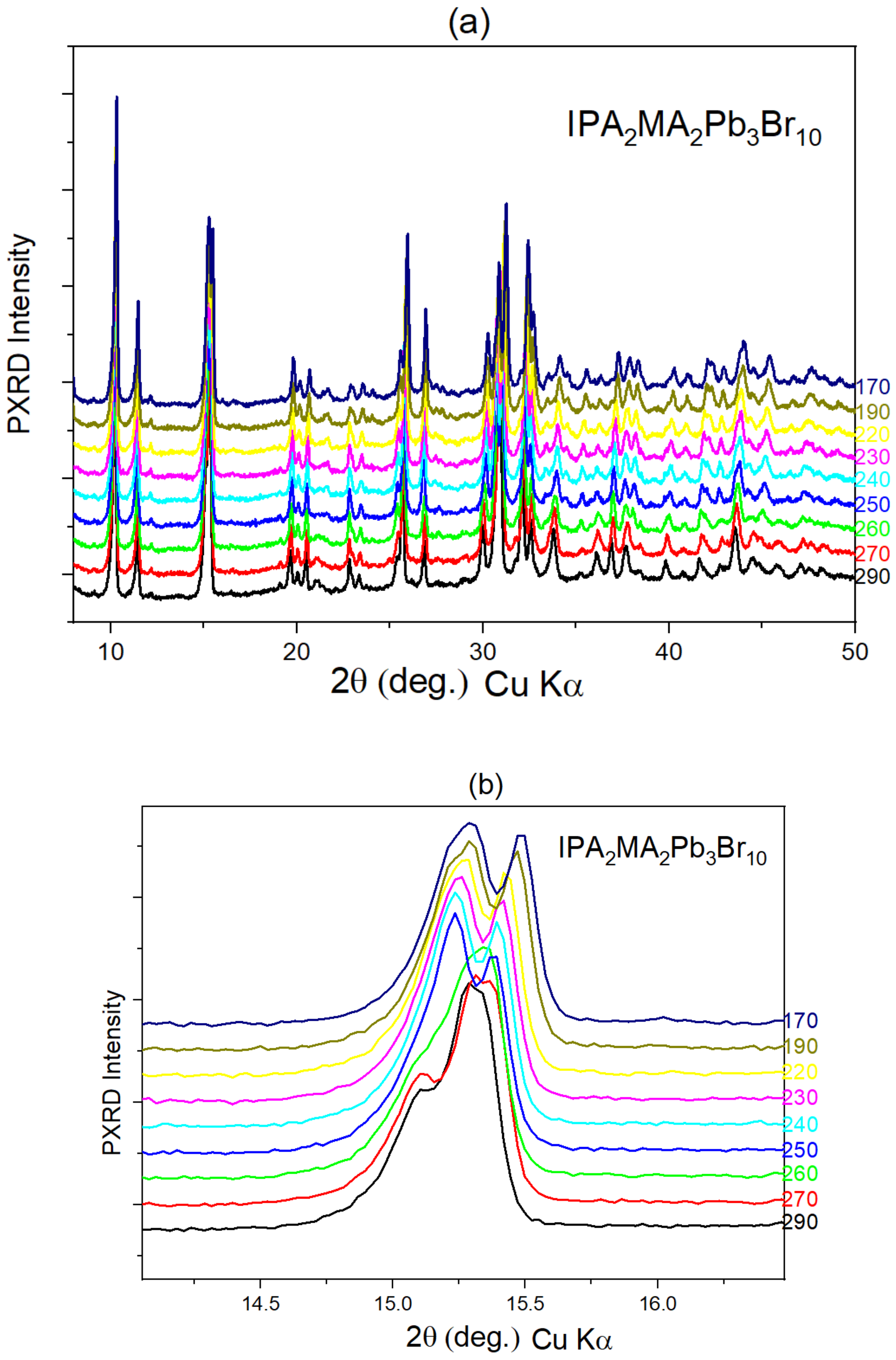

Figure S13. (a) Temperature evolution of PXRD diffraction data in $IPA_2MAPb_3Br_{10}$; (b) temperature changes of the selected (400) and (320) Bragg peaks.

(a)

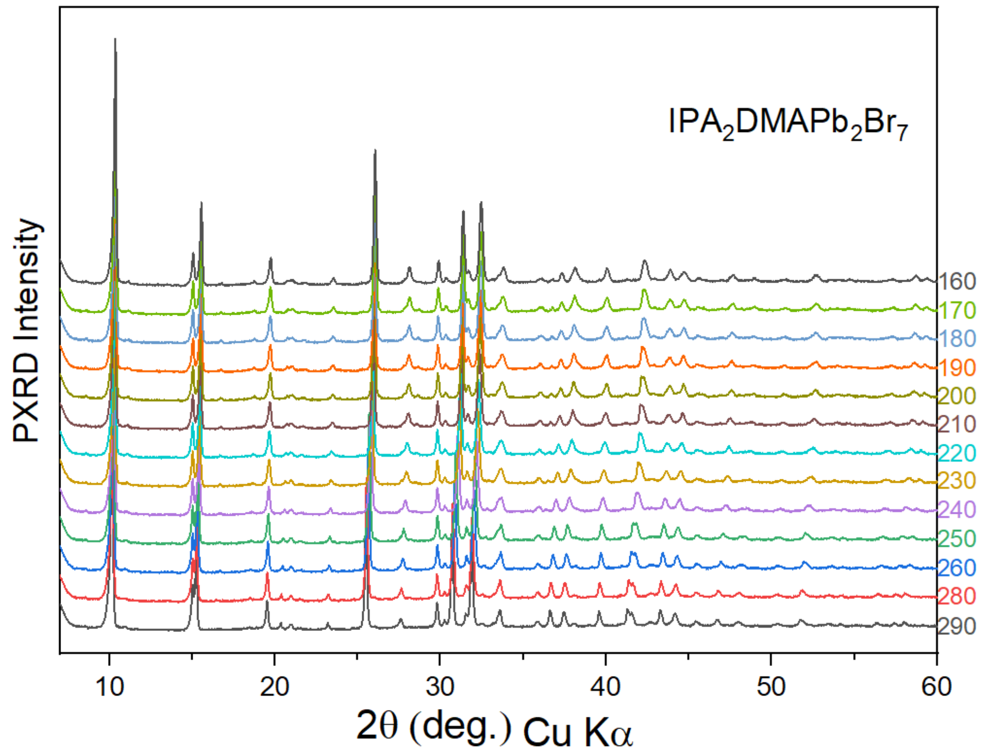
IPA2DMAPb2Br7
PXRD Intensity
160
170
180
190
200
210
220
230
240
250
260
280
290
10
20
30
40
50
60
2θ (deg.) Cu Kα

(b)

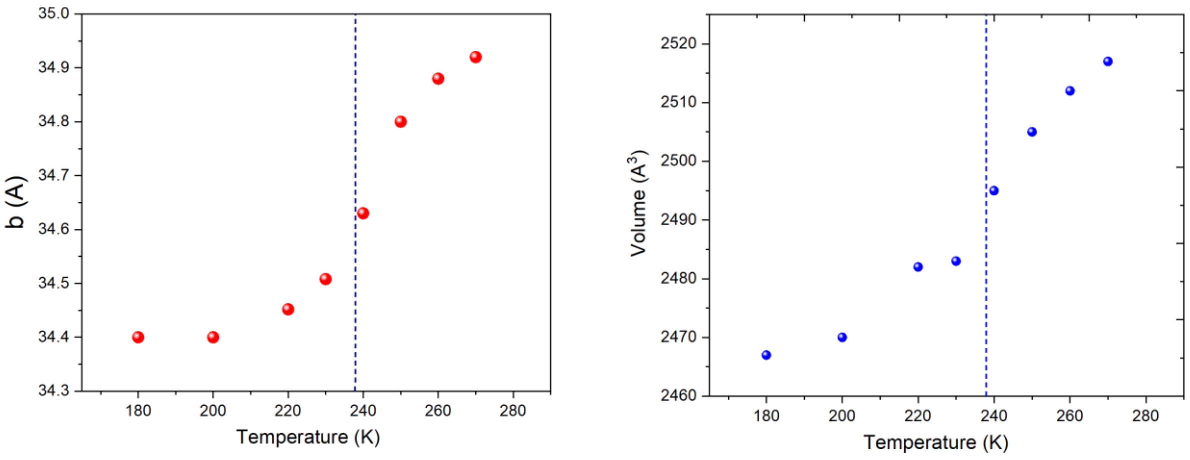
b (A)
Temperature (K)
Volume (A3)
Temperature (K)

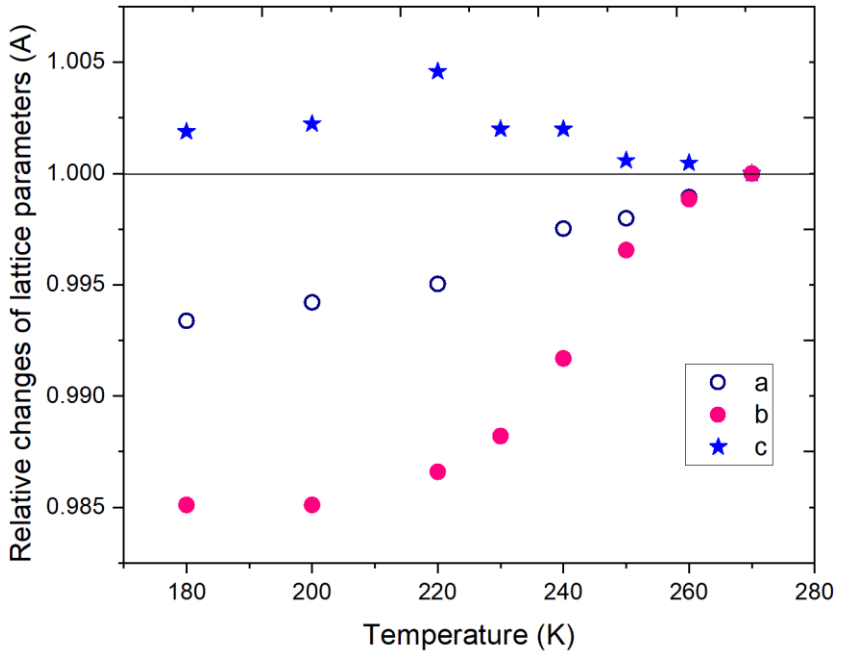

Figure S14. (a) Temperature evolution of PXRD diffraction data in $IPA_2DMAPb_2Br_7$, (b) lattice parameters from single crystal-x-ray diffraction *vs* temperature; *b*-parameter which is perpendicular to the perovskite layers, volume and relative orthorhombic lattice parameters measured while cooling.

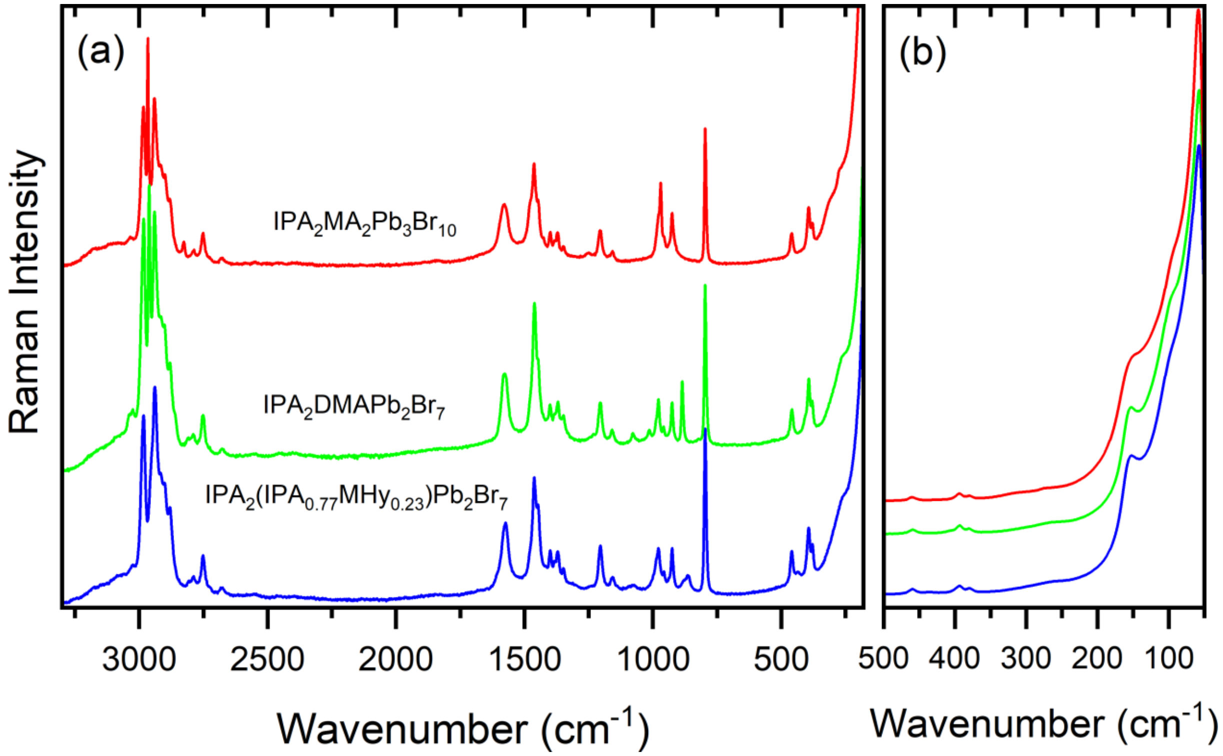

Figure S15. RT FT-Raman spectra of powdered perovskite samples in the (a) 3200-180 cm^{-1} and (b) 500-50 cm^{-1} range.

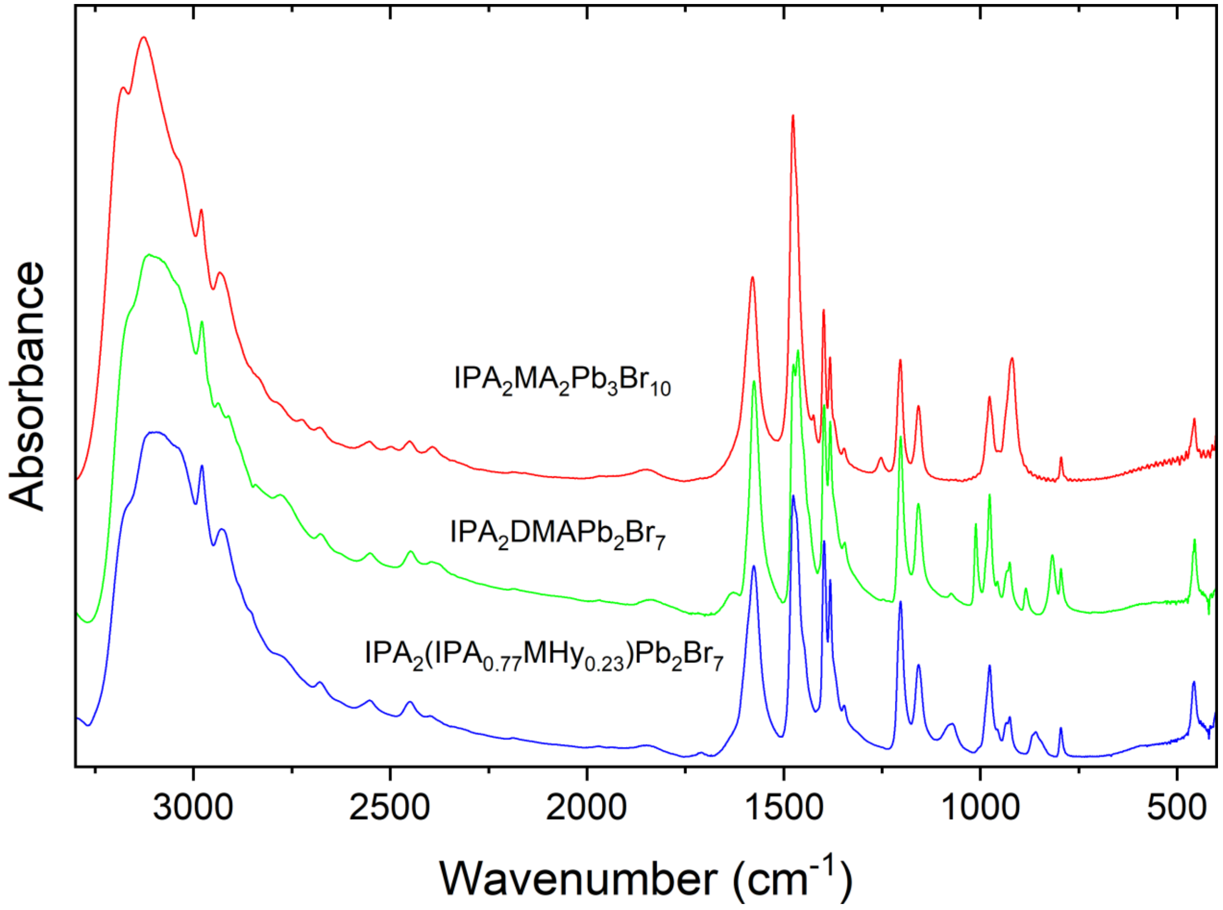

Figure S16. RT FT-IR spectra of powdered perovskite samples in the 3300-400 cm^{-1} range.

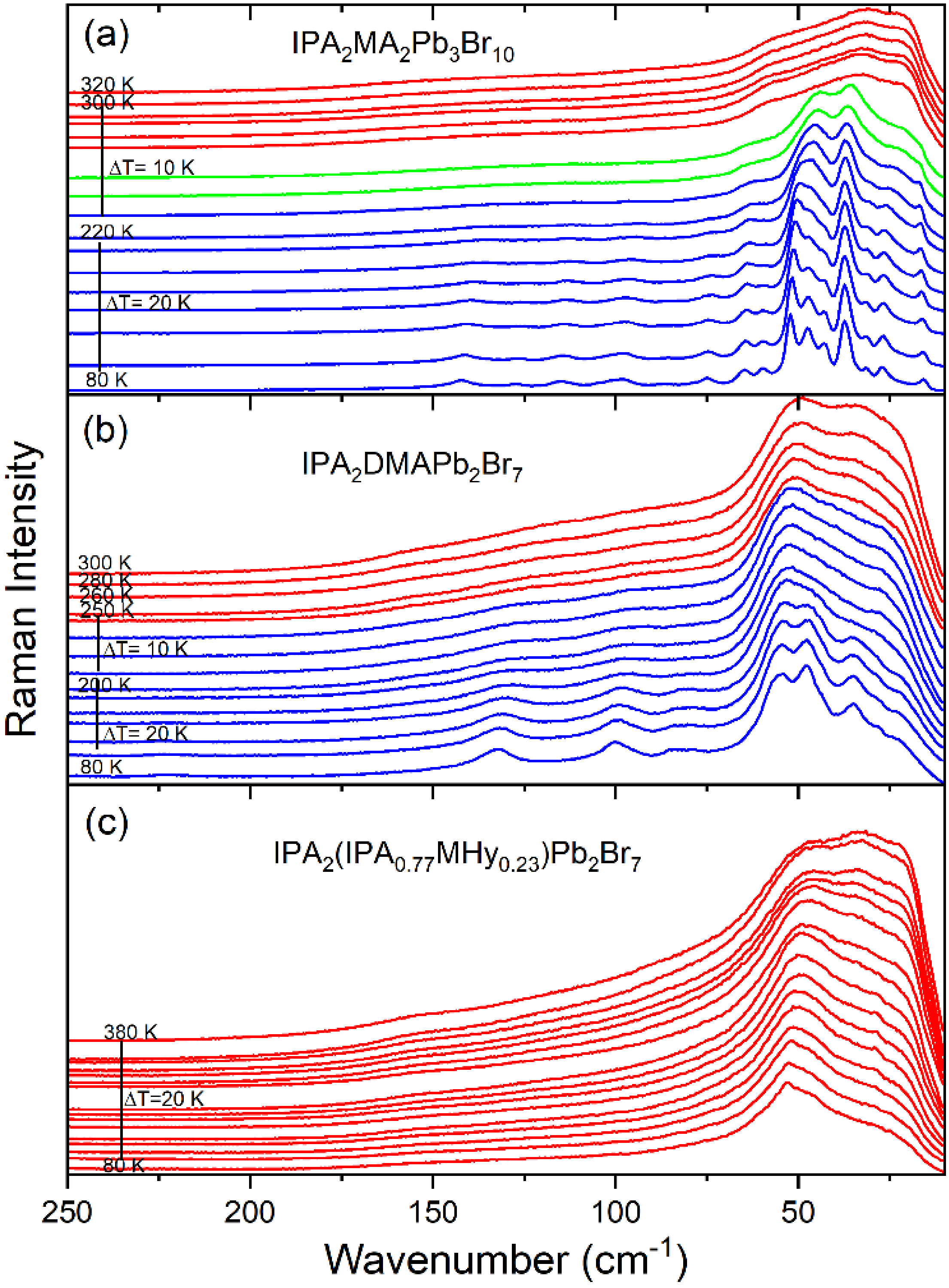

Figure S17. Raman spectra of (a) $IPA_2MA_2Pb_3Br_{10}$, (b) $IPA_2DMAPb_2Br_7$ and (c) $IPA_2(IPA_{0.77}MHy_{0.23})Pb_2Br_7$ in the 250-10 cm^{-1} range recorded on heating. Red, green and blue colors correspond to the HT, IT and LT phase, respectively.

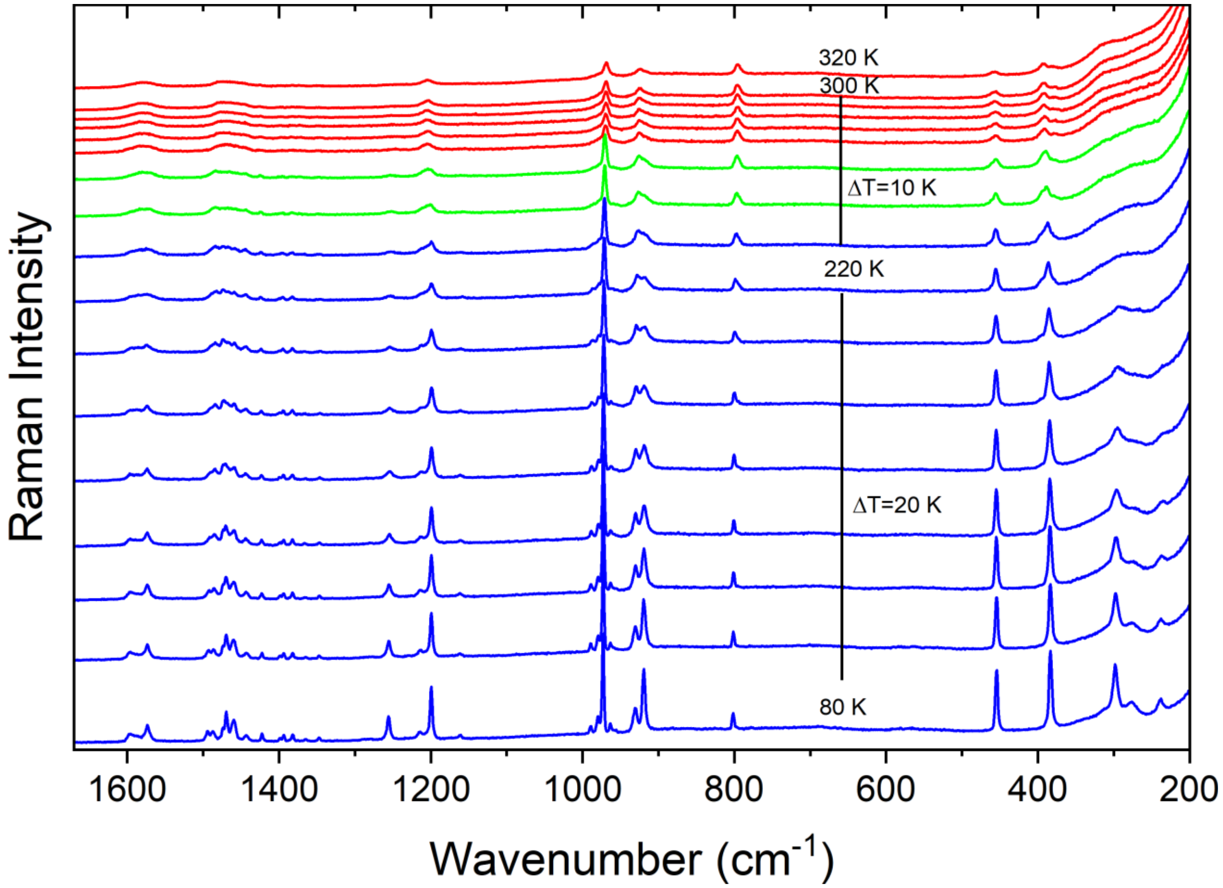

Figure S18. Temperature-dependent Raman spectra of $IPA_2MA_2Pb_3Br_{10}$ in the 1670-200 cm^{-1} range.

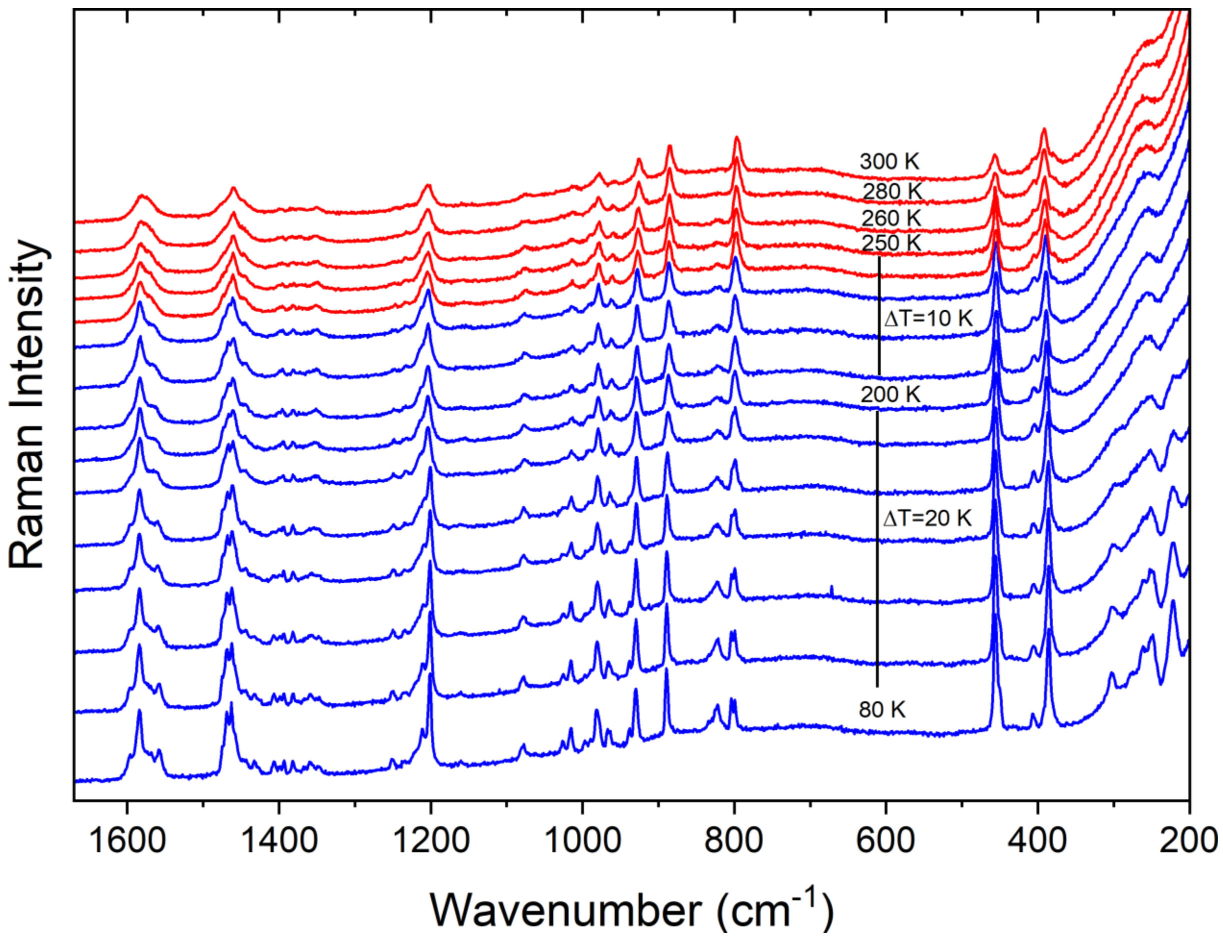

Figure S19. Temperature-dependent Raman spectra of $IPA_2DMAPb_2Br_7$ in the 1670-200 cm^{-1} range.

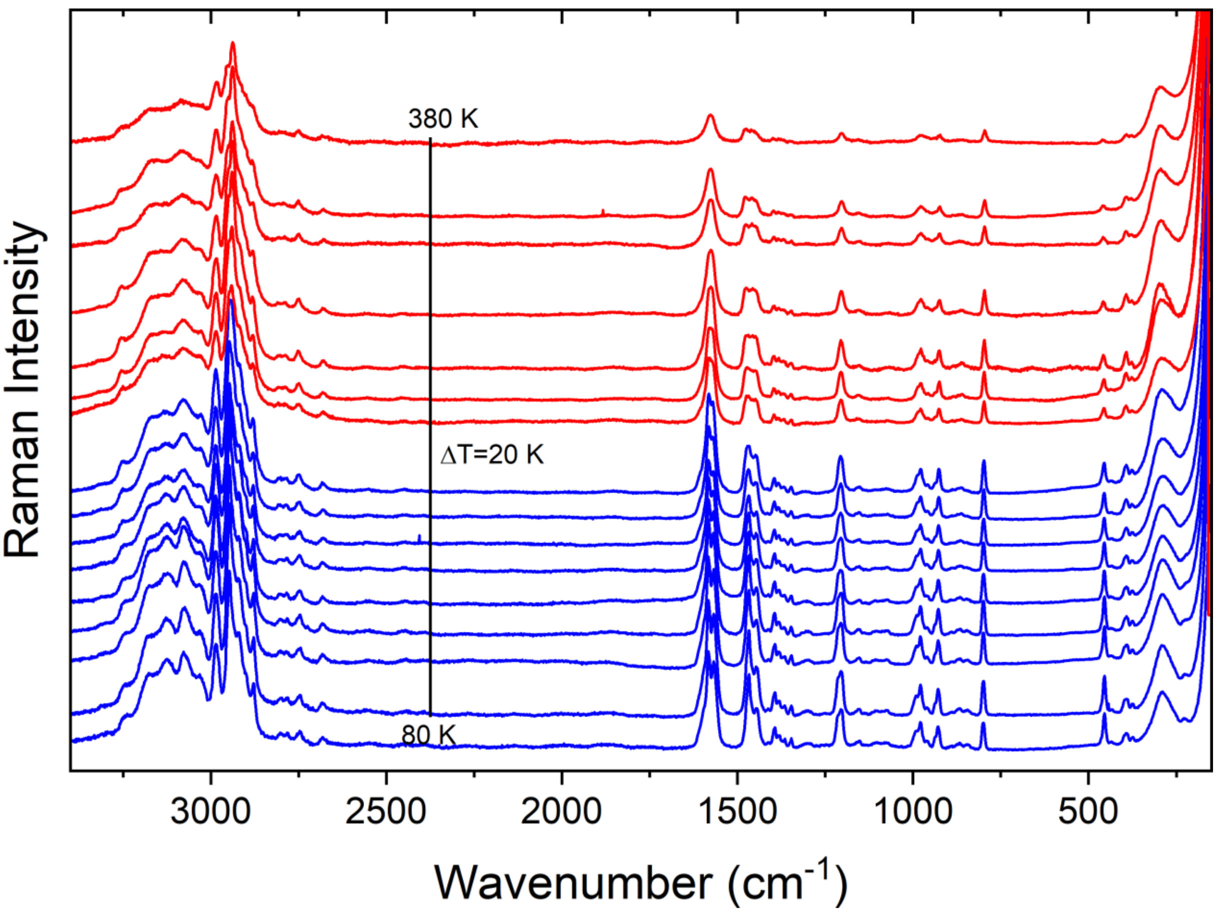

Figure S20. Temperature-dependent Raman spectra of $IPA_2(IPA_{0.77}MHy_{0.23})Pb_2Br_7$ in the 3400-150 cm^{-1} range.

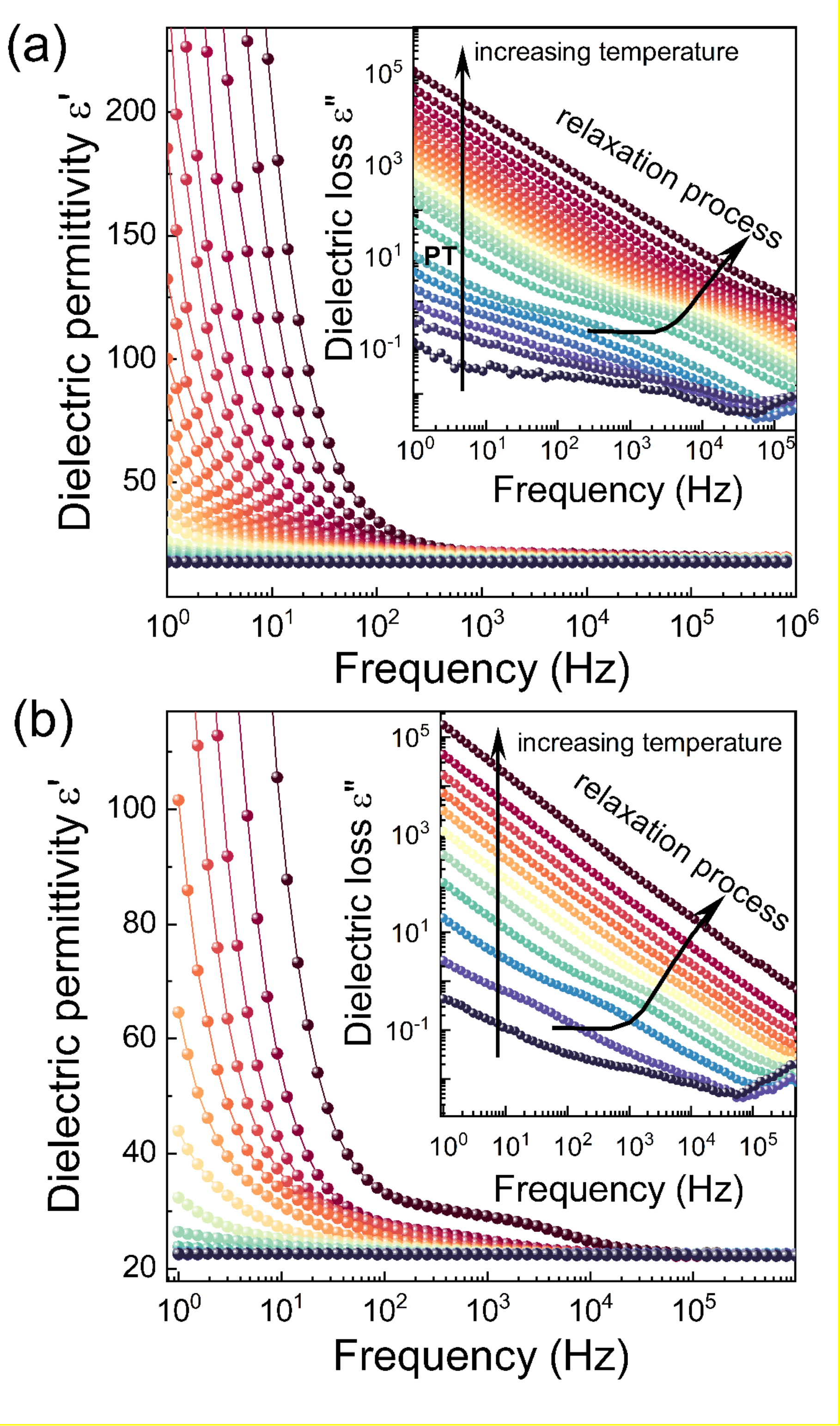

Figure S21. Frequency dependence of the complex dielectric permittivity for (a) $IPA_2MA_2Pb_3Br_{10}$ and (b) $IPA_2DMAPb_2Br_7$.

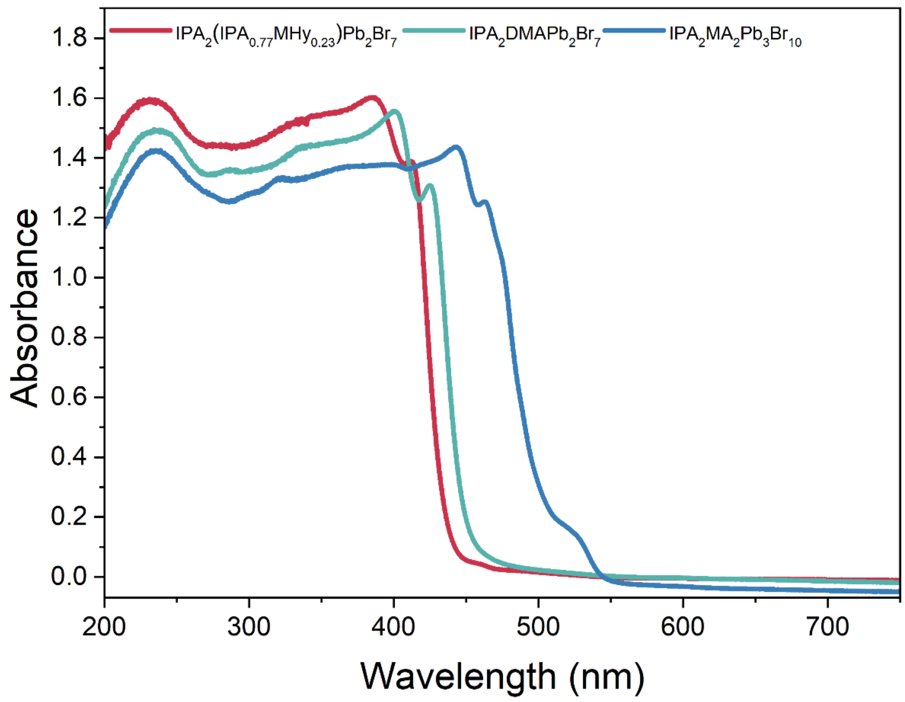

Figure S22. Diffuse-reflectance spectra of $IPA_2MA_2Pb_3Br_{10}$, $IPA_2DMAPb_2Br_7$, and $IPA_2(IPA_{0.77}MHy_{0.33})Pb_2Br_7$ registered at RT.

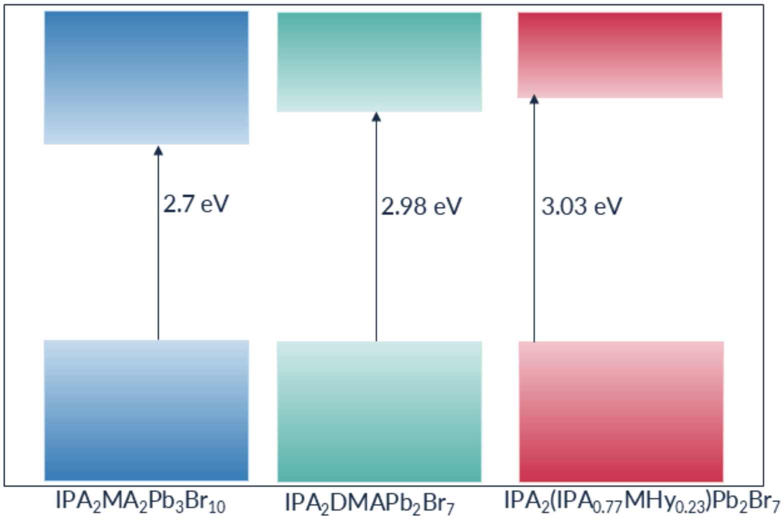

Figure S23. A simple diagram showing the comparison of the energy gap size for the three investigated IPA analogues.

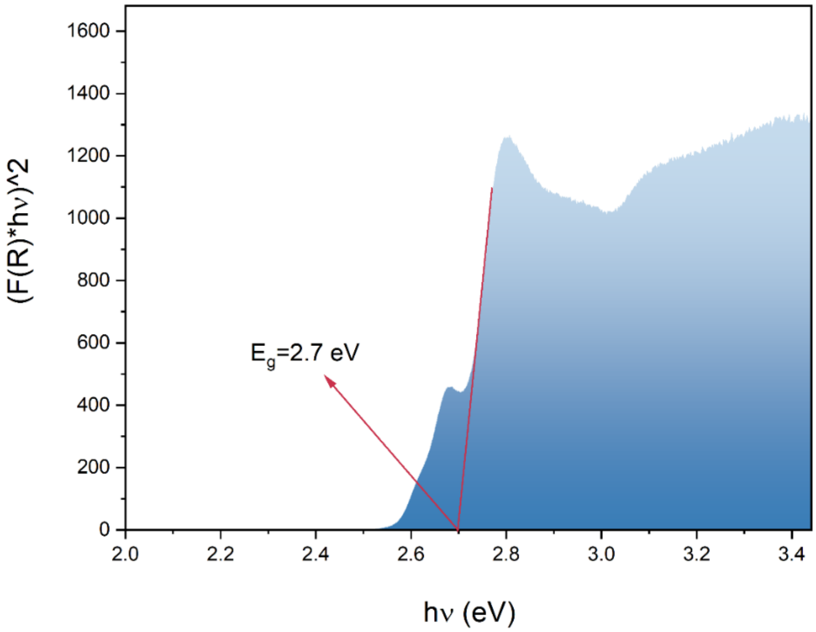

Figure S24. Energy bang gap of $IPA_2MA_2Pb_3Br_{10}$ estimated by x-axis intersection points of the linear fits of the Tauc plots.

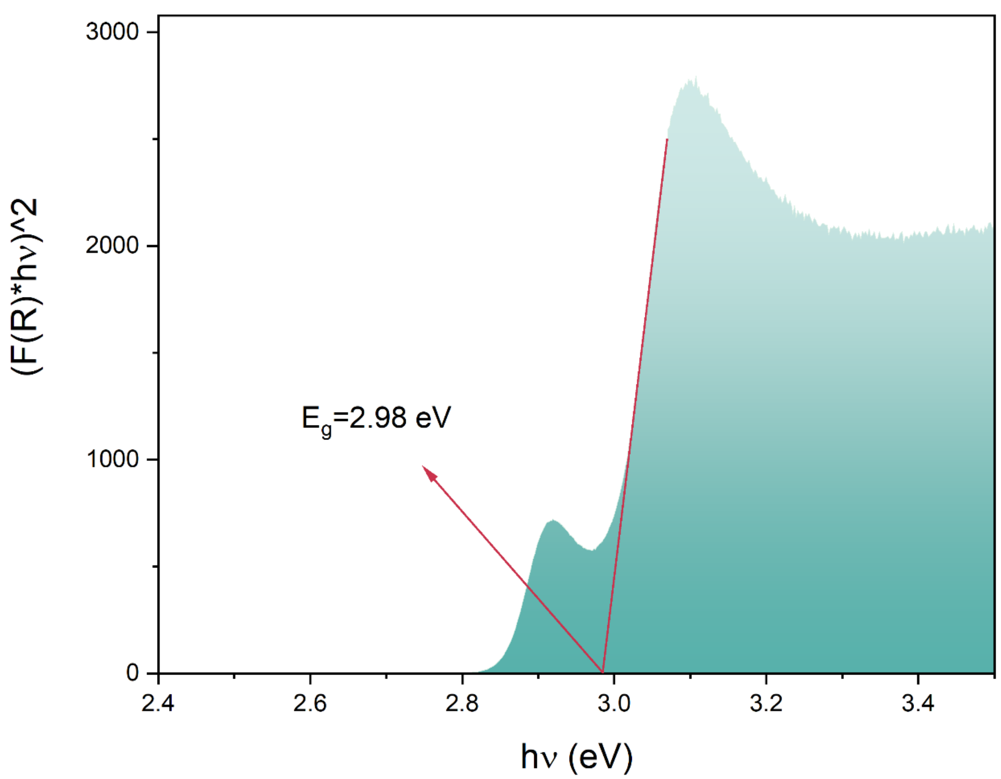

Figure S25. Energy bang gap of $IPA_2DMAPb_2Br_7$ estimated by x-axis intersection points of the linear fits of the Tauc plots.

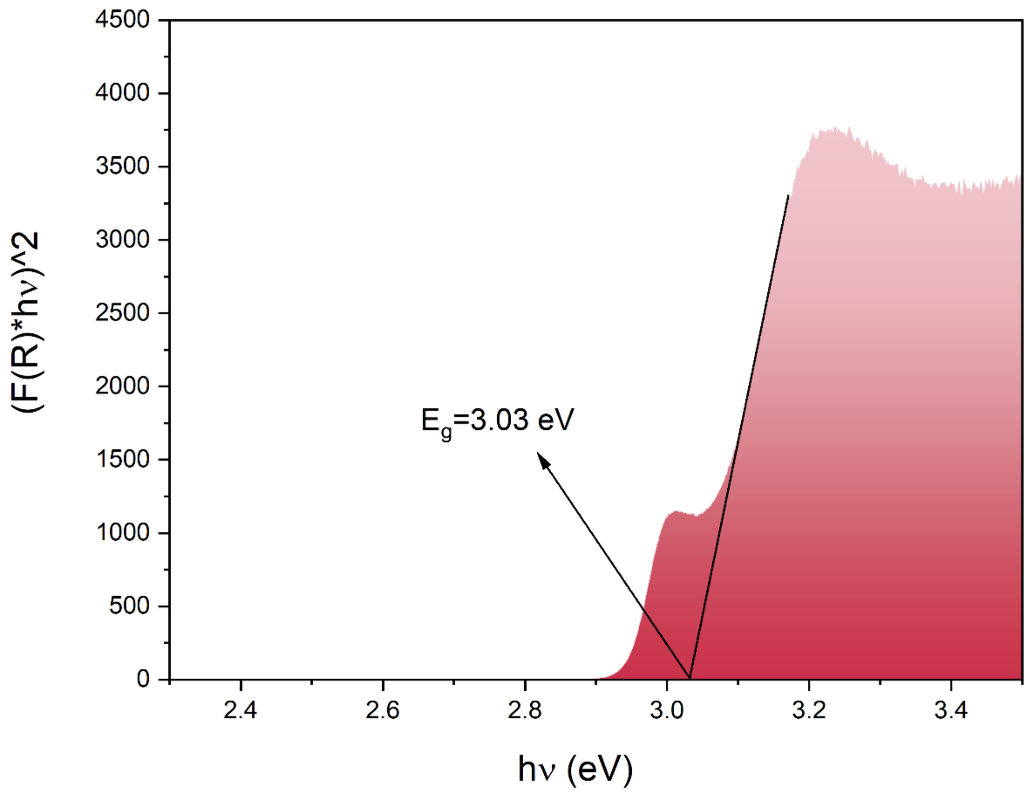

Figure S26. Energy bang gap of $IPA_2(IPA_{0.77}MHy_{0.33})Pb_2Br_7$ estimated by x-axis intersection points of the linear fits of the Tauc plots.

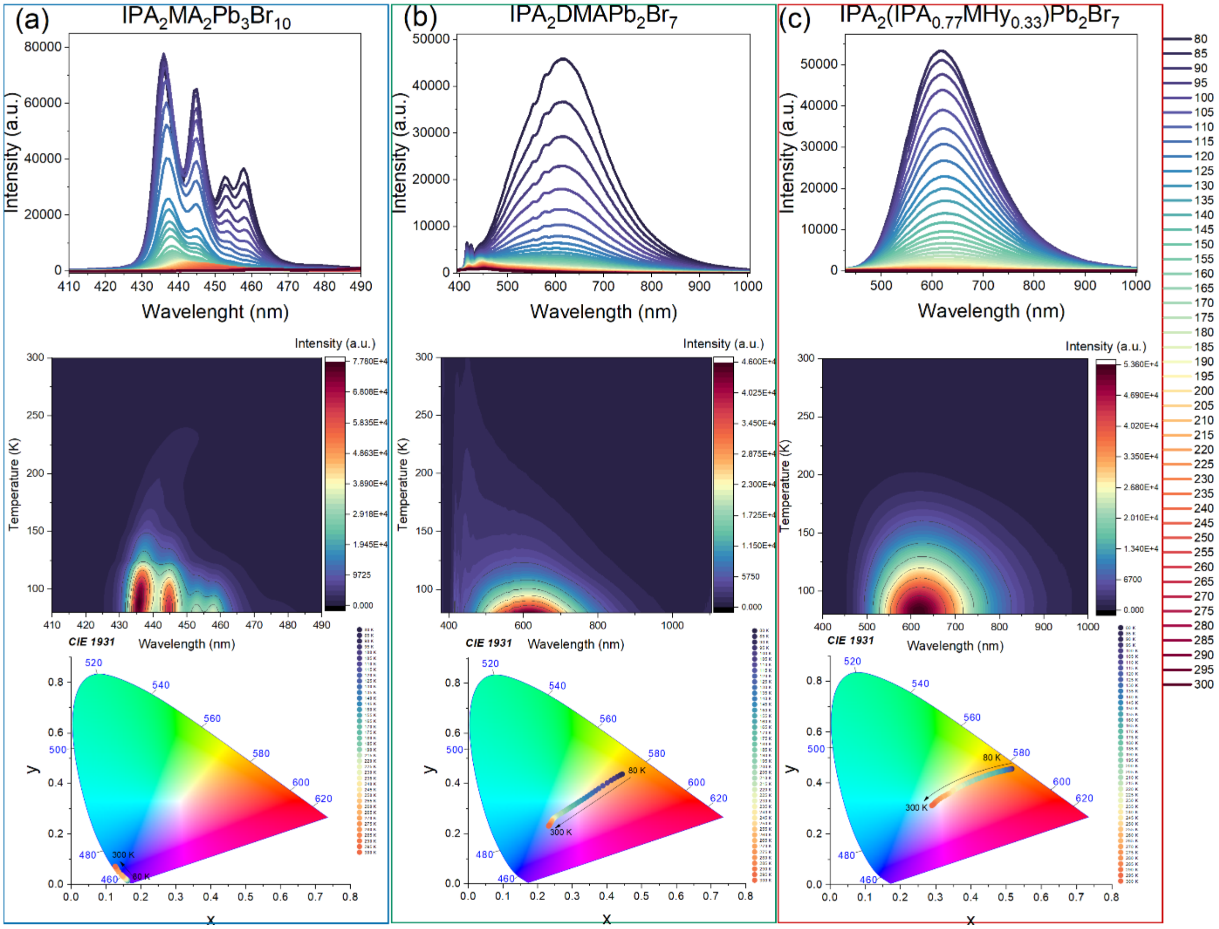

Figure S27. PL spectra registered from 80 K to 300 K every 5 K, PL intensity contour maps, and temperature dependence of CIE chromaticity for (a) $IPA_2MA_2Pb_3Br_{10}$, (b) $IPA_2DMAPb_2Br_7$, and (c) $IPA_2(IPA_{0.77}MHy_{0.33})Pb_2Br_7$.

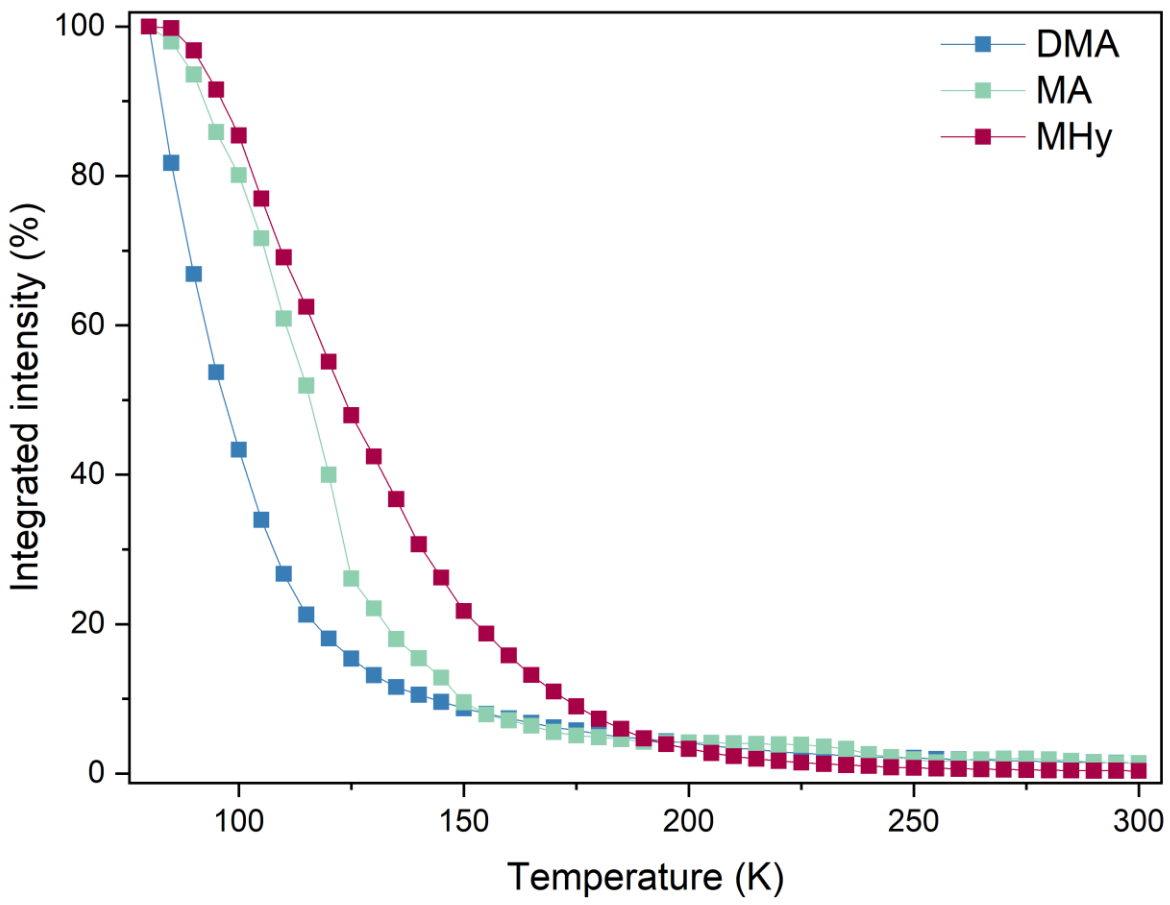

Figure S28. Changes of PL intensity in the function of the temperature for $IPA_2MA_2Pb_3Br_{10}$, $IPA_2DMAPb_2Br_7$, and $IPA_2(IPA_{0.77}MHy_{0.33})Pb_2Br_7$ perovskites.

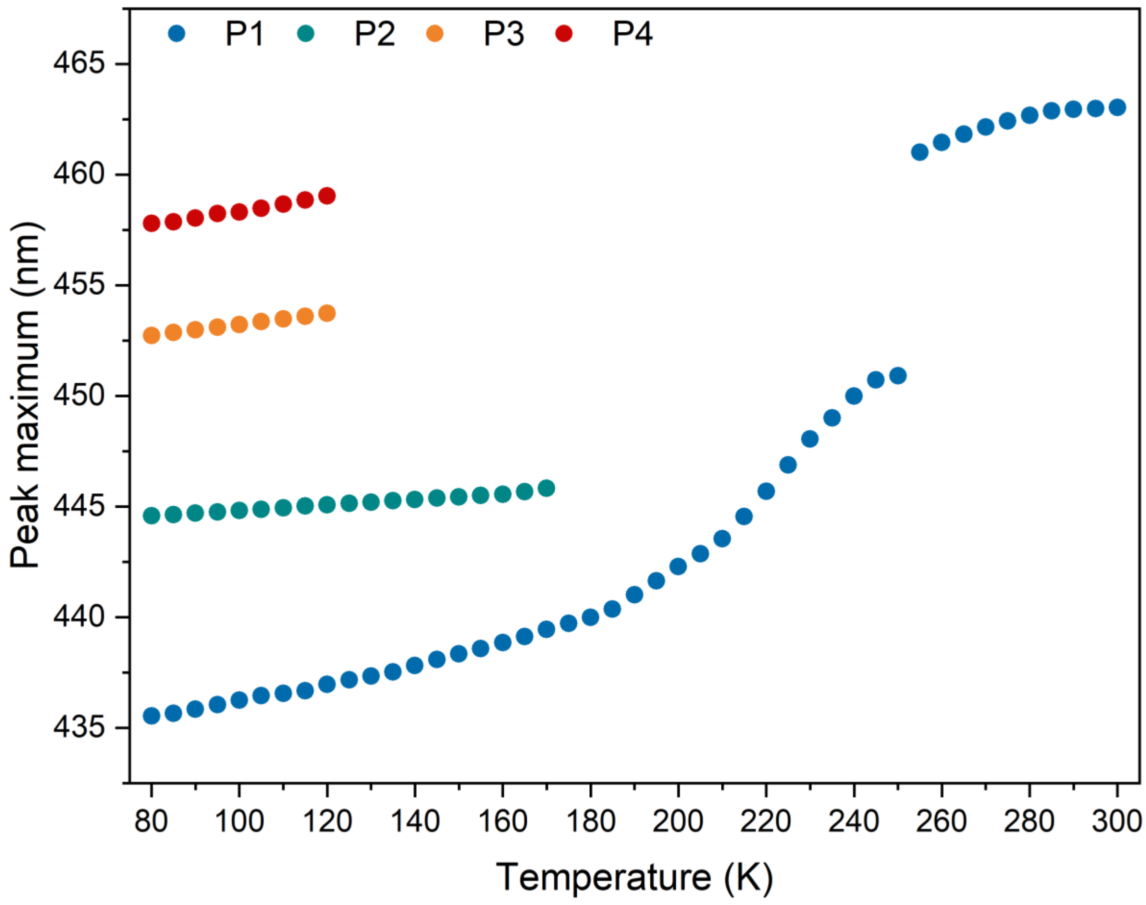

Figure S29. Changes of the PL maximum band position of $IPA_2MA_2Pb_3Br_{10}$ with temperature.

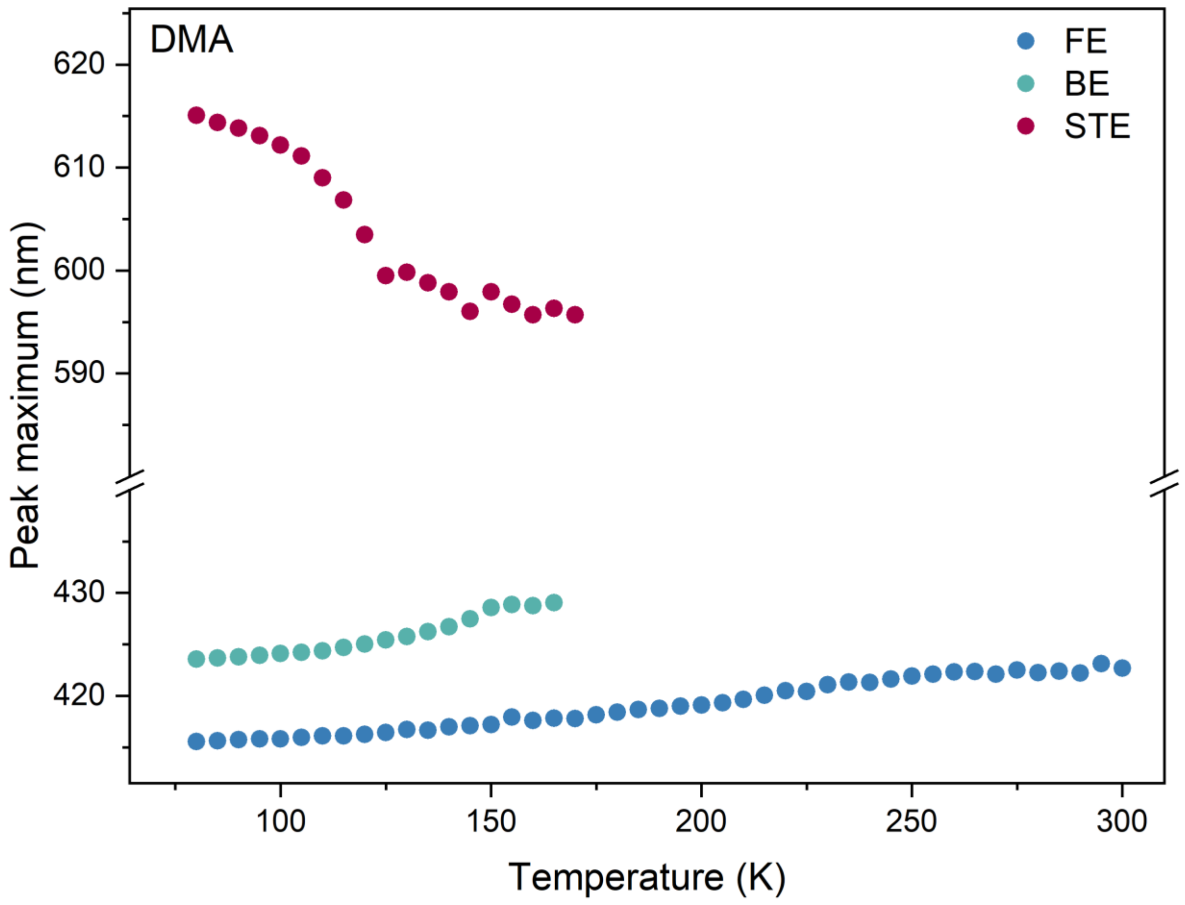

Figure S30. Changes of the PL maximum band position of $IPA_2DMAPb_2Br_7$ with temperature.

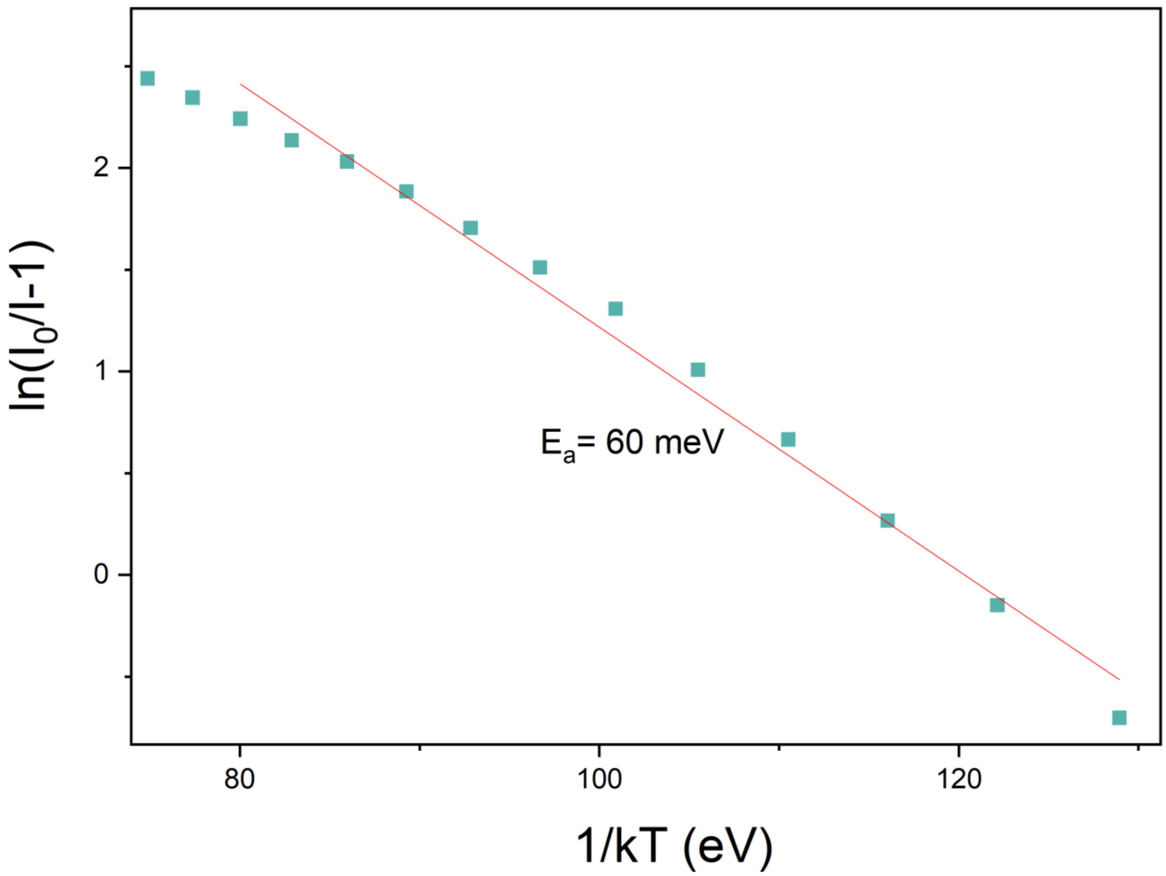

Figure S31. Energy activation of thermal quenching of PL of $IPA_2DMAPb_2Br_7$.

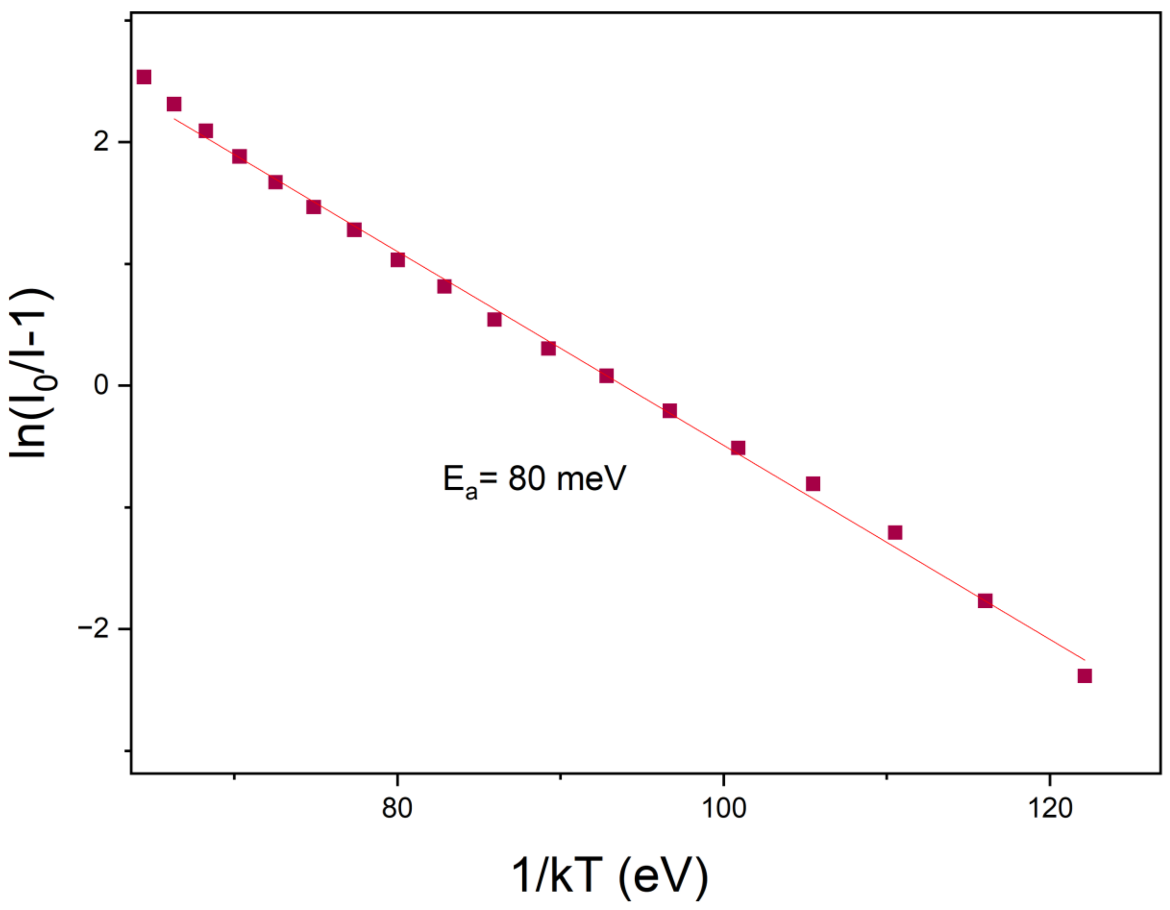

Figure S32. Energy activation of thermal quenching of PL of $IPA_2(IPA_{0.77}MHy_{0.33})Pb_2Br_7$.

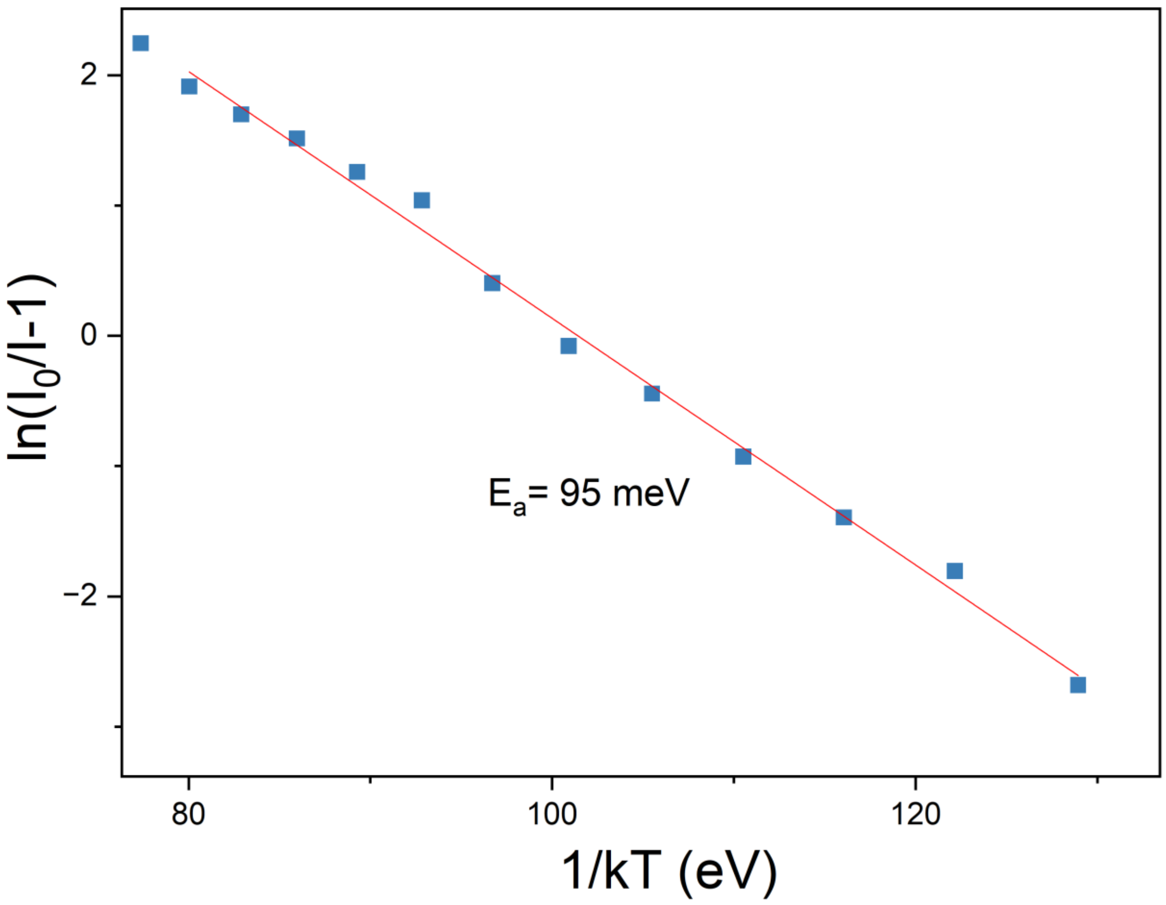

Figure S33. Energy activation of thermal quenching of PL of $IPA_2MA_2Pb_3Br_{10}$.
